# Engineering Free Volume within Frontal Ring‐Opening Metathesis Polymerization via Pendant Plasticization

**DOI:** 10.1002/adma.202519676

**Published:** 2025-12-23

**Authors:** Kevin A. Stewart, Francesca J. Lombardi, Sky D. Cao, Claire M. Massouh, Jacob J. Lessard

**Affiliations:** ^1^ Department of Chemistry University of Utah Salt Lake City Utah USA

**Keywords:** entanglement, frontal polymerization, frontal ring‐opening metathesis polymerization, material manufacturing, reactive manufacturing

## Abstract

Frontal ring‐opening metathesis polymerization (FROMP) enables rapid, energy‐efficient access to high‐performance thermosets and thermoplastics, but the range of accessible properties remains constrained by the rigidity of norbornene‐type backbones. Here we introduce a side‐chain plasticization strategy for FROMP, wherein norbornene esters bearing *n*‐alkyl groups of varying length (*n* = 8, 12, 16) are copolymerized with dicyclopentadiene (DCPD) or hydrogenated DCPD (DCPD‐H_2_). Systematic incorporation of these pendants tunes free volume, resulting in predictable reductions in glass transition temperature (*T*_g_), decreased moduli, and a transition from rigid thermosets to elastomers exceeding 800% elongation at break. Free‐volume analysis via dynamic mechanical analysis and solvent swelling ratios confirms pendant length and distribution as key parameters governing network porosity and mobility. Moreover, high‐alkyl‐content formulations exhibit nonlinear front propagation (spin modes) and strain‐induced whitening—features that highlight opportunities for spatial patterning and cooperative molecular alignment under load. Collectively, these results establish side‐chain engineering as a versatile design principle for expanding FROMP into elastomeric regimes, providing a scalable pathway to soft, tunable, and structurally programmable materials.

## Graphical abstract

Norbornene‐based copolymers with tunable n‐alkyl pendant length provide control over matrix free volume, enabling elastomeric materials via frontal ring‐opening metathesis polymerization (FROMP).

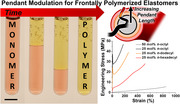

## 1 Introduction

**FIGURE 1:**
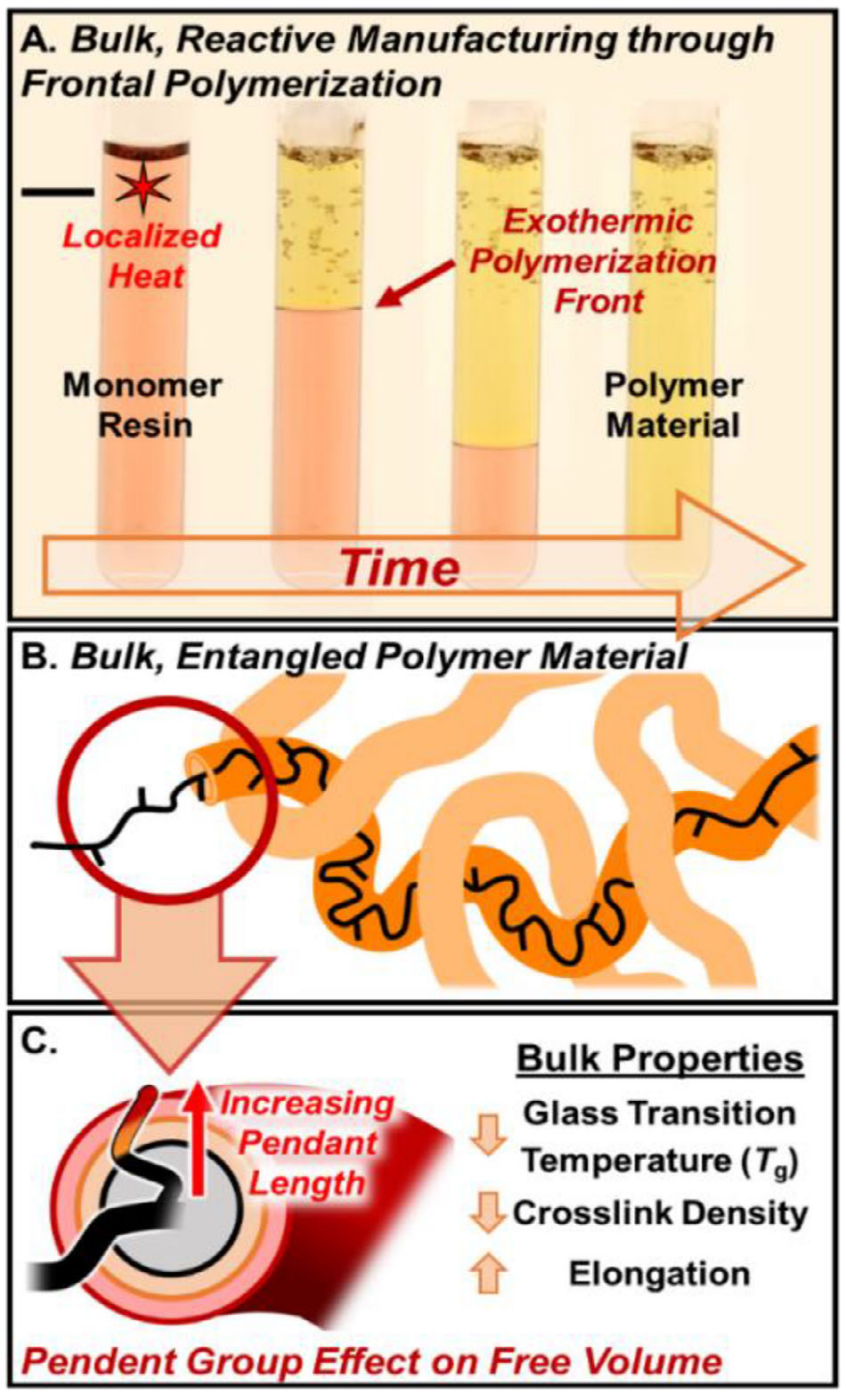
A) Depiction of reactive manufacturing of a polymer monolith through frontal ring‐opening metathesis polymerization (FROMP). B) Cartoon depiction of bulk, entangled polymer chains in a reptation model simplified as a virtual tube. C) This work, wherein the bulk properties of a polymeric material prepared by FROMP are tuned by engineering the free volume of the reptation tube, achieved through systematic increase of (co)monomer pendant length.

Frontal ring‐opening metathesis polymerization (FROMP) offers a compelling alternative for synthesizing advanced polymeric materials, uniquely combining rapid processing with minimal external energy input (Figure 1A) [1, 2, 3, 4, 5, 6, 7, 8, 9, 10]. Driven by a self‐sustaining exothermic front arising from the release of ring strain in cyclic olefins, FROMP enables solvent‐free polymerizations under thermally autonomous conditions—features that align well with current efforts toward more sustainable polymer manufacturing. This unique method has permitted novel approaches to additive manufacturing and the fabrication of composites in a host of material applications [11, 12, 13, 14, 15, 16]. Moreover, recent work has demonstrated that FROMP can be leveraged to synthesize polymers in a well‐controlled manner, enabling targetable primary chain lengths and low molecular weight distributions without necessitating a well‐regulated environment (i.e., dilute conditions and removal of oxygen) [17, 18].

Despite this progress, the monomer scope of FROMP remains somewhat narrow, limiting the range of material properties that are readily accessed through FROMP vs other chain‐growth polymerization methods (e.g., radical polymerization of vinyl monomers) [19]. In particular, the generation of soft materials possessing flexibility and elasticity at or below room temperature via FROMP generally remains underexplored [20], owing to intrinsic rigidity of the norbornene backbones typically employed. Here, we address this limitation by introducing side‐chain plasticization into norbornene‐based monomers, enabling free‐volume engineering as a strategy to access elastomeric FROMP materials with tunable glass transition temperatures (*T*_g_) and viscoelasticity—without modifying the polymer backbone.

In 2020, Moore and Sottos reported that robust elastomers could be rapidly prepared by FROMP of 1,5‐cycloocatadiene (COD) and dicyclopentadiene (DCPD) by systematic modulation of COD loading in the mixed monomer resins [21]. However, the inclusion of COD in FROMP formulations can cause embrittlement of the final material due to crystallization of the COD segments. Furthermore, COD is susceptible to chain‐transfer events, complicating the synthesis and characterization of well‐defined polymer backbones [22]. Thus, expanding the available monomer scope to include inherently plasticizing comonomers capable of reducing the *T*_g_ without alteration of the backbone structure offers a promising strategy for fabricating elastomeric FROMP‐based materials. This approach enables adaptable thermomechanical properties while maintaining the unique advantages of frontal polymerization.

A foundational structure‐property relationship in polymeric materials is side‐chain engineering, where systematic variation of pendant length and mobility enables precise control over thermomechanical and viscoelastic behavior (Figure 1B) [23, 24, 25, 26]. Exemplified in poly(meth)acrylates, increasing side‐chain size from methyl to butyl disrupts interchain packing and enhances segmental mobility, resulting in marked reductions in *T*_g_ from ∼100°C in poly(methyl methacrylate) to near room temperature in poly(butyl methacrylate) [27, 28, 29]. Such structural modifications not only lower *T*_g_ but profoundly influence bulk rheology and mechanical properties, yielding materials with lower moduli and increased elasticity.

Inspired by these design principles, we report a strategy to access elastomeric thermosets and thermoplastics via FROMP of norbornene‐based monomers by modulating the free volume of the polymer matrix (Figure 1C). By copolymerizing DCPD or hydrogenated DCPD (DCPD‐H_2_) with norbornene esters bearing *n*‐alkyl side chains of increasing length (*n* = 8, 12, 16), we achieve rapid, solvent‐free synthesis of soft materials with tunable thermomechanical properties—all while preserving the advantageous kinetics and scalability of frontal polymerization. Interestingly, certain high‐free‐volume formulations exhibit nonlinear front propagation, or spin‐mode instabilities—an emerging feature in FROMP systems. Our findings contribute to the growing understanding of how monomer structure and resin composition influence front behavior, offering potential avenues for spatial control in patterned materials. Ultimately, this work establishes that thermomechanical properties in FROMP‐derived materials can be systematically tuned through side‐chain engineering, presenting an effective alternative to traditional strategies that rely on modification of the backbone microstructure.

## 2 Results and Discussion

To manipulate free volume and access elastomeric behavior, while maintaining a fully norbornene‐based backbone, we synthesized DCPD‐H_2_ [30] and norbornene esters possessing octyl (NBE8), dodecyl (NBE12), and hexadecyl (NBE16) pendants via Stieglitz esterification of 5‐norbornene‐2‐carboxylic acid. The norbornene monomers were prepared on a >30 g scale and isolated in moderate to good yields (62–75%) with excellent purity (Figures S1–S18). With monomers in hand, we began our investigations by studying the copolymerization of the NBE monomers with DCPD‐H_2_ to generate linear copolymers with target degrees of polymerization (DP) ranging from 200 to 4000 (Figure 2A). To establish an upper limit in NBE loading, we initially copolymerized 50 mol% NBE8 with DCPD‐H_2_, given that NBE8 has the lowest molecular weight and thereby the highest heat density of the NBE series. FROMP resins of 50 mol% NBE8 were prepared at monomer:initiator:inhibitor loadings of 200:1:1, 500:1:1, 1000:1:1, and 2000:1:1. In the 200, 500, and 1000:1:1 loadings, the FROMP of 50 mol% NBE8 resulted in distinctive non‐linear front propagation (i.e., spin modes) (Figures S19–S24 and Video S1). We attribute this phenomenon to the significantly elevated average monomer molar mass with 50 mol% incorporation of NBE8 (192.30 g/mol) which leads to perturbations in thermal transport resulting from low heat density of the monomer mixture. At 2000:1:1, the non‐linear front propagation was no longer sustainable and resulted in quenching of the FROMP. Therefore, we identified 25 mol% loading of NBE8, 12, and 16 as an idealized feed in the FROMP resins to ensure a sustained, linear front propagation across formulations due to the increasing molar masses of NBE12 and NBE16. At 25 mol% incorporation, both NBE8 and NBE12 maintained linear FROMP at all target DPs (Figure 2B; Figures S25–S44). Interestingly, the FROMP of 25 mol% NBE16 again gave rise to spin modes at 500, 1000, 2000, and 4000:1:1 (Figures S45–S52 and Video S2). However, the patterned front quenched at a loading of 200:1:1 which we attribute to a combination of the elevated inhibitor concentration and low heat of reaction (*H*_r_) resulting from nearly identical average monomer molar mass (191.32 g/mol) as the 50 mol% NBE8 formulation. As such, cure kinetics from differential scanning calorimetry (DSC) confirm that a decrease in *H*_r_ is concomitant with the increasing average monomer molecular weight of the resin mixtures (Figure 2C; Figures S53–S73, and Table S2). We also observed that the front velocity (*v*_f_) of the NBE copolymerizations decreased as the overall incorporation of NBE *n*‐alkyl content increased, attributable to the attentuation of heat generation with increasing NBE molar mass (Figure 2D and Table S1). Nevertheless, the final polymer monoliths across the formulations displayed minimal internal defects at loadings above 200:1:1, indicating that a range of molecular weights can be effectively implemented in their reactive manufacturing (Figures S74–S90).

**FIGURE 2:**
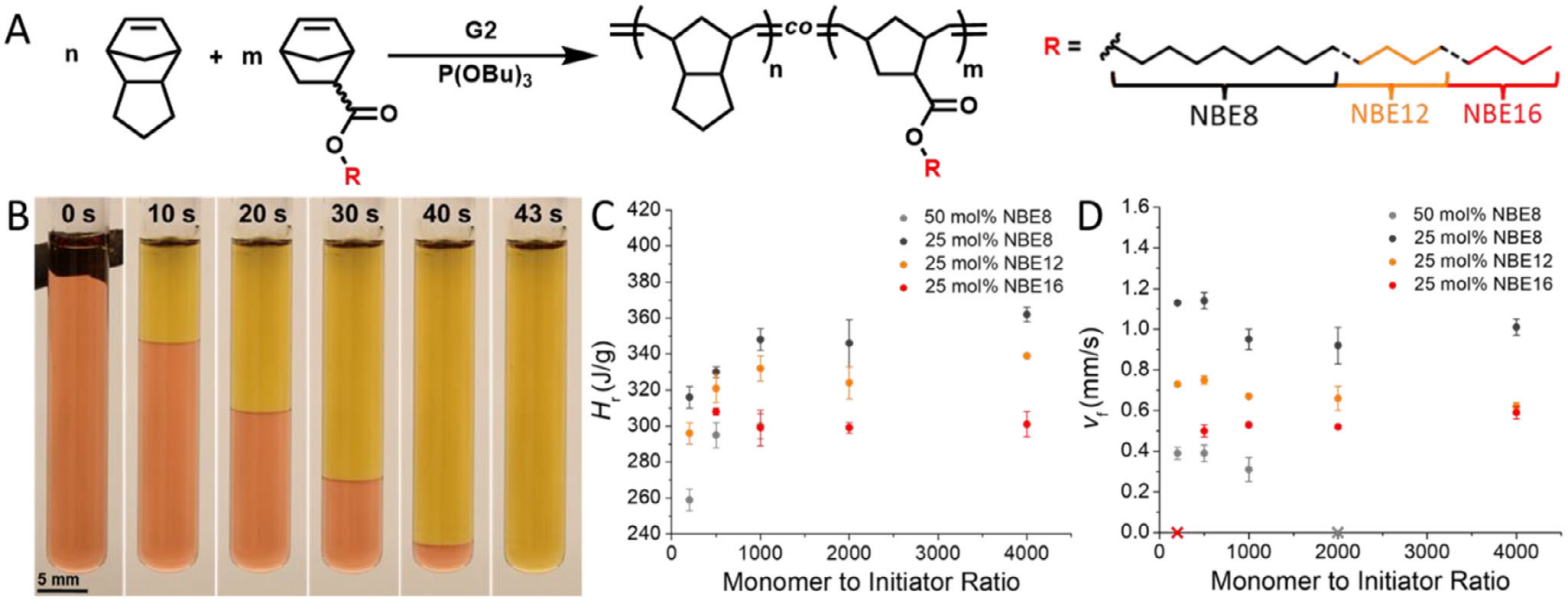
Frontal copolymerization of norbornene ester derivatives (NBE) with hydrogenated dicyclopentadiene (DCPD‐H_2_) for the preparation of linear polymers with varying degrees of polymerization. A) General reaction scheme for the copolymerization of NBE of varying pendant length and DCPD‐H_2_ with Grubbs 2^nd^ generation initiator (G2) and exogenous tributyl phosphite inhibitor (P(OBu)_3_; TBP). B) Representative timelapse of 25 mol% dodecyl‐norbornene ester (NBE12) in DCPD‐H_2_ at a loading of 1000:1:1 (monomer:G2:TBP). C) Heat of reaction (*H*_r_) from cure kinetics of NBE frontal copolymerizations with DCPD‐H_2_ at various monomer to initiator ratios. D) Front velocities (*v*_f_) of NBE frontal copolymerizations with DCPD‐H_2_ at various monomer to initiator ratios.

**FIGURE 3:**
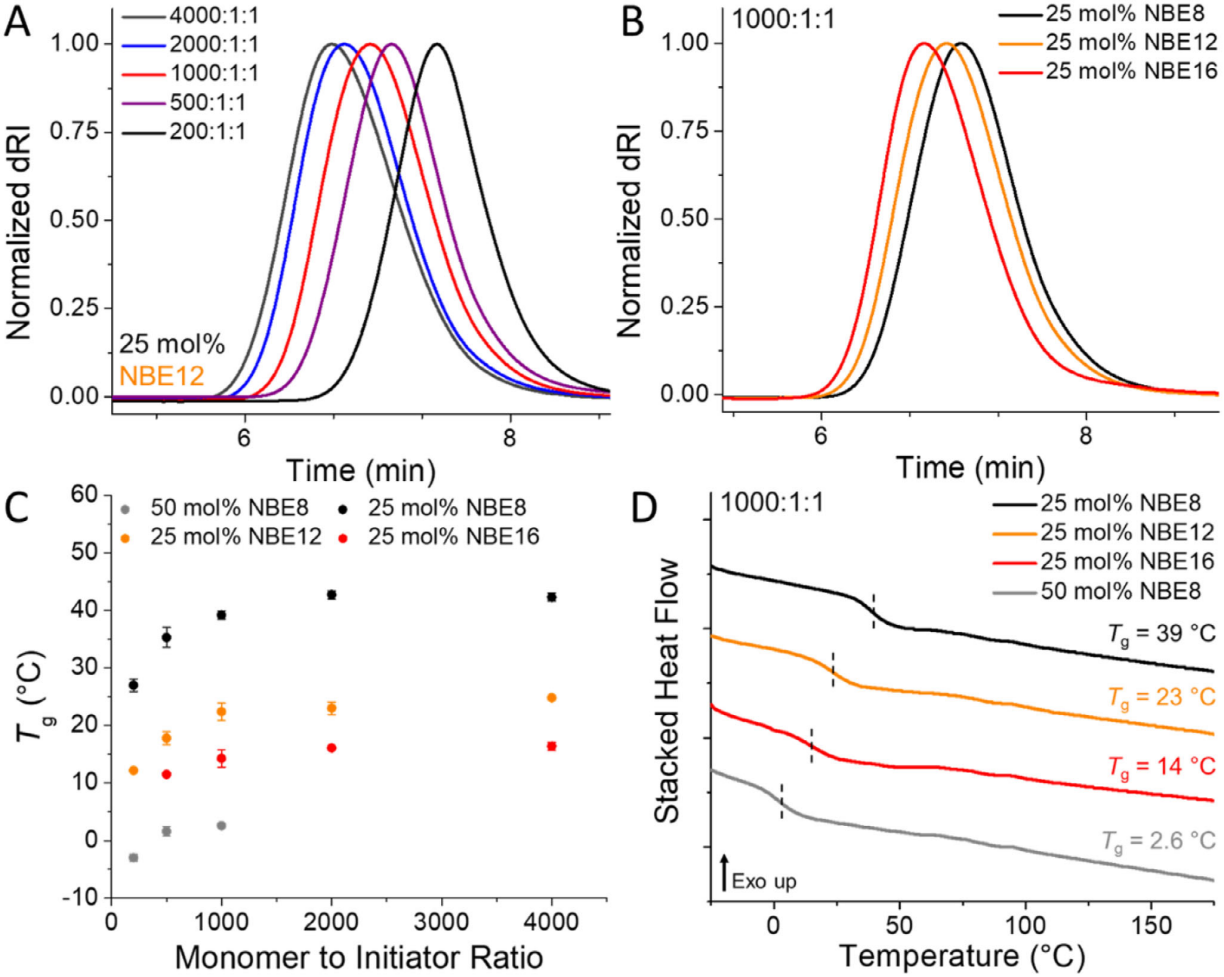
Post‐cure analysis of linear copolymers prepared by frontal copolymerization of norbornene ester derivatives and hydrogenated dicyclopentadiene (DCPD‐H_2_). A) Size‐exclusion chromatography (SEC) analysis of copolymers of norbornene dodecyl ester (NBE12) and DCPD‐H_2_ of increasing target degrees of polymerization (monomer:initiator:inhibitor). B) Representative SEC eluograms of copolymers prepared from 25 mol% norbornene ester (NBE) in DCPD‐H_2_ at a loading of 1000:1:1 (monomer:initiator:inhibitor). C) Glass transition temperature (*T*_g_) values of NBE copolymers with DCPD‐H_2_ across monomer to initiator ratios showing a decrease in *T*_g_ below a loading of 1000:1:1 (monomer:initiator:inhibitor) D) Differential scanning calorimetry (DSC) thermograms of NBE copolymers with DCPD‐H_2_ at a loading of 1000:1:1 (monomer:initiator:inhibitor) demonstrating the relationship between pendant length and distribution and polymer plasticity.

The cured FROMP samples were removed and cut into cross sections for analysis by size‐exclusion chromatography (SEC), DSC, and ^1^H NMR spectroscopy. SEC analysis indicated that all NBE frontal copolymerizations allowed for general primary chain length control dependent on target DP (Figure 3A; Figures S91–S94); however, the experimental molecular weights were lower than theoretical values at loadings of 1000:1:1 and higher, while consistently maintaining high dispersity (*Ð* = 1.35–1.66) that increased in higher target DP resins (Figures S95 and S96 and Table S3). These findings are consistent with previous reports on controlled FROMP wherein equimolar loading of initiator and inhibitor gives rise to poor chain length control and high *Ð* values due to the poor initiation efficiency of G2 [18]. We therefore identified 1000:1:1 FROMP loadings as the optimal monomer to initiator ratio (Figure 3B) due to diminishing returns in achievable primary chain length above 1000:1:1 and a characteristic decrease in glass transition temperatures (*T*_g_) below 1000:1:1 (Figure 3C). This decrease may be attributable to either plasticization arising from increased inhibitor concentration or loss of primary chain entanglement. Regardless, the thermal properties of the final FROMP‐based thermoplastics displayed a dramatic decrease in the *T*_g_ values at 1000:1:1 as compared to materials comprised of poly(DCPD‐H_2_) and previously reported copolymers, with values ranging from 39 ± 1 to 2.6 ± 0.3°C for 25 and 50 mol% NBE8, respectively (Figure 3D; Figures S97–S113, and Table S4). Post‐cure analysis by DSC indicated that residual monomers may be contributing to the remarkably low *T*_g_ of the 50 mol% NBE8 copolymer (85% conversion from *H*_r, residual_); however, all three 25 mol% NBE copolymers achieved >95% conversion by DSC (Table S5). Finally, analysis by ^1^H NMR spectroscopy confirmed statistical incorporation of the NBE monomers with DCPD‐H_2_, with excellent agreement of theoretical and experimental molar incorporation (Figures S114–S130 and Table S6). Taken together, the data confirms that side chain plasticization is a potent strategy for achieving room temperature flexibility in FROMP‐based materials without compromising primary chain microstructure.

**FIGURE 4:**
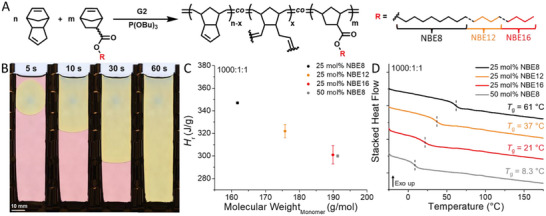
Frontal copolymerization of norbornene ester derivatives (NBE) with dicyclopentadiene (DCPD) for the preparation of thermoset polymers. A) General reaction scheme for the copolymerization of NBE of varying pendant length and DCPD with Grubbs 2^nd^ generation initiator (G2) and exogenous tributyl phosphite inhibitor (P(OBu)_3_; TBP). B) Representative timelapse of 25 mol% dodecyl‐norbornene ester (NBE12) in DCPD at a loading of 1000:1:1 (monomer:G2:TBP) in 3 mm U‐molds conducted at 40°C. C) Heat of reaction (*H*_r_) from cure kinetics of NBE frontal copolymerizations with DCPD at a loading of 1000:1:1 (monomer:G2:TBP). D) Differential scanning calorimetry (DSC) thermograms of NBE copolymers with DCPD at a loading of 1000:1:1 (monomer:initiator:inhibitor) demonstrating free volume control within crosslinked systems.

**FIGURE 5:**
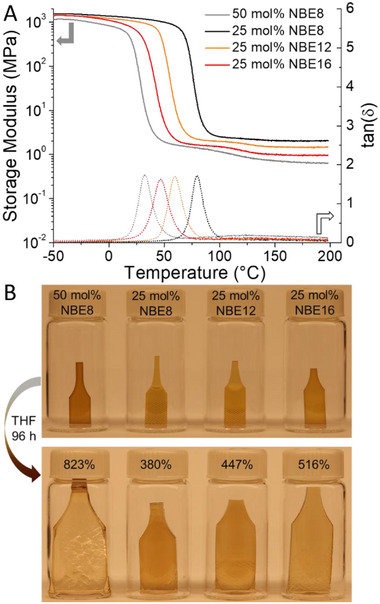
A) Dynamic mechanical analysis of network polymers prepared by frontal copolymerization of norbornene ester derivatives (NBE) with dicyclopentadiene (DCPD) showing tunable free volume depending on pendant length. B) Swelling ratios of crosslinked NBE copolymers with DCPD in tetrahydrofuran following 96 h of incubation of solvent at room temperature further confirming tunable network free volume.

Having achieved room temperature elasticity in FROMP‐based linear copolymers, we then applied this strategy to prepare crosslinked elastomers by frontal copolymerization of the NBE monomers with DCPD, the latter bearing a second metathesis site enabling network formation. The FROMP resins were maintained at our optimized loading of 1000:1:1 (Figure 4A). Gratifyingly, the NBE monomers readily solubilized DCPD, which typically necessitates the addition of low wt.% ethylidene norbornene as an additive to suppress the melting temperature of the waxy monomer [16]. Furthermore, we conducted the subsequent FROMP of the NBE resin mixtures at 40°C, given the decreased thickness of the U‐mold geometry as compared to the glass tubes (3 mm vs 6 mm, respectively) and to ensure that non‐linear front propagation was avoided (Figure 4B), as patterned FROMP materials have been shown to elicit dissimilar mechanical properties compared to their non‐patterned counterparts [31]. The elevated environmental temperature successfully eliminated spin modes in the 50 mol% NBE8 and 25 mol% NBE16 resins, and all FROMP formulations enabled the rapid fabrication of defect‐free monoliths within 2 min, with *v*_f_ ranging from 1.02 ± 0.02 to 0.47 ± 0.01 mm/s (Figures S131–S140 and Table S7). Cure kinetics of the DCPD‐NBE resins agreed well with the comparable DCPD‐H_2_ formulations (vide supra), with *H*_r_ values (347 ± 1 to 300 ± 1 J/g) decreasing as the average monomer molar mass increased owing to reduction of resin heat density (Figure 4C; Figures S141–S144, and Table S8). The monoliths were then cut into dogbone geometries and rectangular specimens for dynamic mechanical analysis (DMA). Post‐cure samples were cut from remaining fragments and analyzed by DSC, revealing elevated *T*_g_ values compared to their linear counterparts (Figure 4D and Figure S145–S148). This is attributable to the significantly higher *endo*‐DCPD content, whereas stereochemical inversion occurs during the synthesis of DCPD‐H_2_ yielding nearly exclusive *exo* monomer orientation [17, 32, 33], which has been shown to result in lower *T*_g_ of pDCPD materials [18]. However, the inclusion of static crosslinks and subsequent segmental restriction is an additional contributing factor. Regardless, the resultant materials possessed *T*_g_ values ranging from 61 ± 1 to 8.3 ± 0.3°C, decreasing monotonically with increasing pendant content (Table S9). Post‐cure analysis also revealed that the elevated environmental temperature resulted in greater monomer conversion in the 50 mol% NBE8 resin, achieving a conversion of 93 ± 1% as determined by residual *H*_r_ values (Table S10). Finally, we confirmed our ability to control polymer matrix free volume through DMA temperature sweeps and swelling behavior of the networks in tetrahydrofuran (THF). DMA experiments clearly evidenced a percolated network structure, with rubbery plateau moduli (*E*’_rp_) evidenced over 100°C past their *T*_g, DMA_ values (determined at the peak of the dampening factor, or tan (δ maximum) (Figure 5A; Figures S149–S152, and Table S11). Moreover, the relationship between *n*‐alkyl content and *T*_g, DMA_ values agreed well with DSC studies. We further verified the network architecture by preparing linear controls of 50 mol% NBE8 and 25 mol% NBE16 given their comparable alkyl content and room temperature elasticity. DMA thermograms of the linear copolymers displayed a clear and consistent decrease in their rubbery plateau until the specimens ultimately yielded due to viscoelastic flow at elevated temperatures (Figures S153 and S154). We then quantified the free volume of the networks using Equation S1 to calculate their relative molecular weight between crosslinks (*M*_x_) by extrapolation of *E*’_rp_ at 180°C, assuming a Poisson's ratio of 0.4, and using the measured densities of the materials as determined through the Archimedes principle (Figure S155 and Table S12). Interestingly, the *M*_x_ values of the networks show a strong linear correlation across the 25 mol% NBE formulations, with *M*_x_ values of 5310 ± 140, 7690 ± 470, and 10600 ± 360 g/mol for 25 mol% NBE8, NBE12, and NBE16, respectively (Figure S156). However, *M*_x_ increases significantly to 17120 ± 1230 g/mol for 50 mol% NBE8, indicating that a higher distribution of plasticizing pendants of shorter length has a marked effect on free volume, as 25 mol% NBE16 has comparable global *n*‐alkyl content albeit at lower density along the primary chain. These findings were reinforced by the swelling ratios of the NBE networks following immersion in THF for 96 h at room temperature, with ratios of 380 ± 8, 447 ± 17, 516 ± 17% for 25 mol% NBE8, NBE12, and NBE16, respectively (Figure 5B; Table S13). In agreement with the trend evinced in DMA experiments and the *M*_x_ values, 50 mol% NBE displayed a dramatic increase in swelling capability with a ratio of 823 ± 60%. In this way, we verified that the porosity (free volume) of the networks scales both with increasing pendant length and distribution, enabling larger uptake of solvent molecules. Collectively, the data demonstrates that free volume modification can be leveraged to access readily tunable thermomechanical and solvochemical properties in frontally polymerized DCPD‐based thermosets by systematic inclusion of *n*‐alkyl pendants of various lengths and backbone distribution.

**FIGURE 6:**
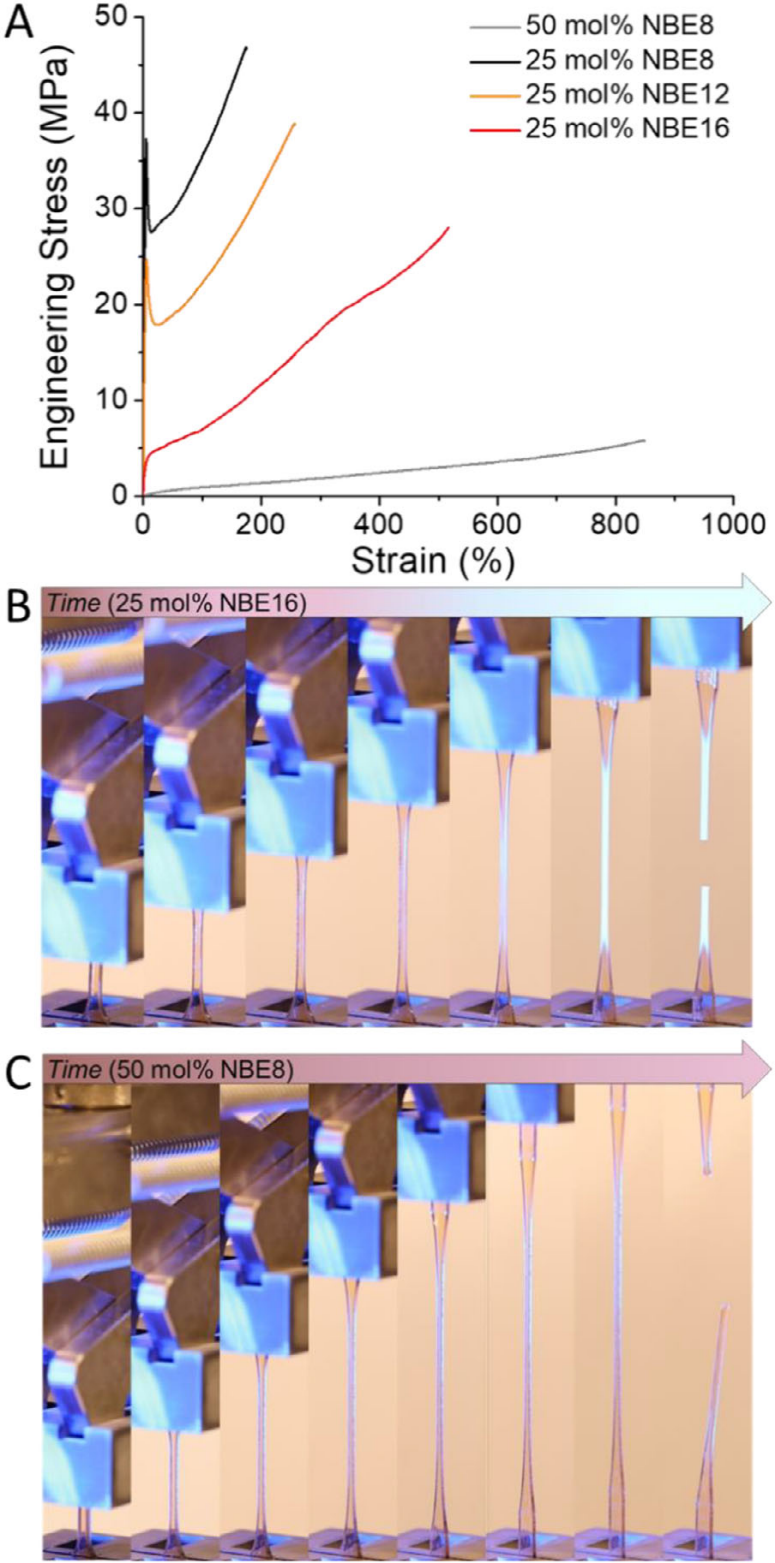
A) Stress‐strain curves from tensile testing of network polymers prepared by frontal copolymerization of norbornene ester derivatives (NBE) with dicyclopentadiene (DCPD) showing tunable mechanical properties depending on the extent of pendant plasticization. B) Representative timelapse of 25 mol% NBE16 in DCPD tensile test showing strain‐whitening prior to failure. C) Representative timelapse of 50 mol% NBE8 in DCPD tensile test showing lack of strain‐whitening prior to failure.

This method of controlling the free volume and polymer plasticity (i.e., *T*_g_) allows for a broad range of mechanical properties to be accessed via FROMP without alteration of the primary chain microstructure. Tensile testing revealed exceptional stiffness and toughness in the 25 mol% NBE8 and NBE12 networks (*T*_g_ > room temperature), with Young's moduli of 1.19 ± 0.03 and 1.00 ± 0.07 GPa, respectively. Furthermore, these rigidified materials boasted yield strengths of 36.3 ± 2.5 and 25.5 ± 2.2 MPa followed by plastic deformation until reaching maximum elongation at failure of 178 ± 16 and 244 ± 38% for 25 mol% NBE8 and NBE12, respectively (Figure 6A; Table S14). Furthermore, the materials demonstrated clear cold drawing and strain hardening behavior (Figures S157–S160), achieving ultimate tensile strengths of 46.0 ± 2.5 and 42.2 ± 2.6 MPa for 25 mol% NBE8 and NBE12, respectively. These properties reveal that despite significantly lower *T*_g_ values (>100°C) compared to typical pDCPD prepared by FROMP, comparable stiffness can be achieved, while significantly augmenting achievable deformations, due to their plasticizing pendants providing increased mobility of the polymer backbones under load. This principle is further reinforced in the 25 mol% NBE16 and 50 mol% NBE8 networks (*T*_g_ < room temperature), which present elastomeric properties, indicating a critical *n*‐alkyl pendant length or global content required to achieve plasticity at ambient temperatures. As such, their tensile behavior exhibited exceptional extensibility, with maximum elongation at failure of 519 ± 40 and 842 ± 28% and ultimate tensile strengths of 25.9 ± 1.4 and 5.32 ± 0.37 MPa for 25 mol% NBE16 and 50 mol% NBE8, respectively (Figures S161–S164). To the best of our knowledge, these materials are the most elastomeric norbornene‐based materials prepared by FROMP to date. Furthermore, in the 25 mol% NBE16 network, a unique phenomenon arose at strains exceeding 300%—the emergence of strain‐whitening (Figure 6B). We believe this may be attributable to increased ordering of the polymer matrix induced by strain‐induced alignment of the *n*‐alkyl side chains or primary chain alignment [26, 27, 34, 35], which has only been demonstrated previously in FROMP materials derived from COD through strain alignment of the semi‐crystalline backbones [21]. Unlike the COD‐derived systems, the strain‐induced whitening was rapidly reversible once the applied deformation was removed, either by failure or unloading (Figure S165), currently limiting further characterization (e.g., X‐Ray scattering techniques). However, hysteresis tests confirm reversibility of this whitening. In addition to strain hardening behavior and subsequent whitening, the development of opacity was demonstrated to be strain rate independent, suggesting that crazing is unlikely the operative mechanism (Figures S166–S174). Nevertheless, this behavior was unique to the 25 mol% NBE16 network, as the strain‐whitening did not emerge in the 50 mol% NBE network despite the similar global *n*‐alkyl content, suggesting that a critical *n*‐alkyl pendant length is required to induce the hypothesized molecular alignment (Figure 6C). Furthermore, tensile testing of the linear analogues of 25 mol% NBE16 and 50 mol% NBE8 generally supported this behavior, with significant strain‐whitening emerging in the linear 25 mol% NBE16 copolymer (Figures S175 and S176). However, unlike its crosslinked counterpart, linearized 50 mol% NBE8 began to demonstrate a minor amount of strain‐whitening just before material failure (Figures S177 and S178 and Table S14). This disparity between the network and linear 50 mol% NBE8 may be attributable to a lack of segmental fixity, allowing for more constructive segmental alignment.

## 3 Conclusions

Establishing molecular design principles that enable refined control over thermomechanical properties and processing behavior is critical for advancing FROMP‐based materials across structural and functional applications. The results presented here establish that side‐chain plasticization via systematic incorporation of *n*‐alkyl‐substituted norbornene esters offers a robust strategy to modulate polymer free volume, *T*_g_, and viscoelasticity—without altering the backbone microstructure or sacrificing the advantages of frontal processing. Increasing pendant length and loading leads to predictable alteration of thermomechanical properties, decreased front velocities and heat densities, and enhanced elasticity in both linear and crosslinked materials—establishing free‐volume engineering as a generalizable lever for tailoring FROMP‐derived polymers. Rheology and swelling studies confirm that pendant distribution and size govern network free‐volume and mobility, while tensile testing reveals a clear transition from rigid thermosets to highly extensible elastomers. Finally, the emergence of spin‐mode instabilities in high‐alkyl‐content formulations indicate a broader toolbox for permitting controlled patterning within FROMP‐derived materials. Building on prior demonstrations of spin mode–driven anisotropy [31], our findings further establish the necessary molecular and processing conditions for exploiting this phenomenon in conjunction with segmental ordering under strain. In particular, the emergence of strain‐induced whitening in pendant‐rich networks lays the groundwork for “spatioselective” strain‐induced crystallization—a concept wherein patterned front propagation and molecular alignment could be used cooperatively to encode anisotropic force responsiveness and visual strain sensors within a single monolith. These insights not only demonstrate a scalable approach for tuning polymer properties via free volume engineering but also suggest a path toward structurally programmed materials manufactured entirely through low‐energy, reactive processing.

## Conflicts of Interest

The authors declare no conflicts of interest.

## Supporting information




**Supporting File**: adma71882‐sup‐0001‐SuppMat.pdf

**Supporting File**: adma71882‐sup‐0002‐VideoS1.mp4

**Supporting File**: adma71882‐sup‐0002‐VideoS2.mp4

## Data Availability

The data that support the findings of this study are available in the supplementary material of this article.
