# Supplementary material for: Engineering Free Volume within Frontal Ring‐Opening Metathesis Polymerization via Pendant Plasticization
Source: Adv Mater. 2025 Dec 23;38(10):e19676. doi: 10.1002/adma.202519676 (PMC12910533; doi:10.1002/adma.202519676)
Supplement: Supplementary file 1 — Supporting File: adma71882‐sup‐0001‐SuppMat.pdf [file ADMA-38-e19676-s002.pdf]

Engineering Free Volume within Frontal Ring-Opening Metathesis Polymerization via Pendant Plasticization

Kevin A. Stewart, Francesca J. Lombardi, Sky D. Cao, Claire M. Massouh, and Jacob J. Lessard*

Department of Chemistry, University of Utah, Salt Lake City, Utah 84112, United States of America

E-mail: Jacob.lessard@utah.edu

Supporting Information:

Materials and Instrumentation
Experimental Procedures
FROMP of NBE Monomers and DCPD-H_2
FROMP of NBE Monomers and DCPD
Dynamic Mechanical Analysis
Swelling Studies and Density Measurements
Tensile Testing
References

Materials and Instrumentation SI 3

Experimental Procedures SI 4

Nuclear Magnetic Resonance – SI 5

FROMP of NBE Monomers and DCPD-H_2 SI 17

Front Velocity and Timelapses – SI 20

Differential Scanning Calorimetry (DSC) Cure Kinetics – SI 38

Sample Images – SI 49

Size-Exclusion Chromatography (SEC) Post-Cure – SI 57

DSC Post-Cure – SI 61

NMR Spectroscopy Post-Cure – SI 71

FROMP of NBE and DCPD SI 80

Front Velocity, Timelapses, and Sample Images – SI 81

DSC Cure Kinetics – SI 87

DSC Post-Cure – SI 90

Dynamic Mechanical Analysis SI 93

Density Measurements SI 97

Swelling Studies SI 98

Tensile Testing SI 99

Hysteresis – SI 104

Tensile Testing with Increased Rate – SI 106

Tensile Testing of Samples Containing DCPD-H_2 – SI 108

References SI 112

Materials:

Dicyclopentadiene (DCPD, 95%, Sigma), hydrobromic acid (48%, Sigma), 5-Norbornene-2-carboxylic acid (98%, TCI), 1-octanol, 1-dodecanol, 1-hexadecanol (MilliporeSigma), (3-Dimethylamino-propyl)-ethyl-carbodiimide hydrochloride (EDC•HCl, 98%, Santa Cruz Biotechnology) Grubbs' 2nd generation catalyst (G2, ChemScene), palladium on carbon (10% Pd, 50% Water, Accela) and tributyl phosphite (TBP, TCI chemicals, stored under N_2), diethyl ether (anhydrous, Fisher), hexane (ACS, Fisher), tetrahydrofuran (THF, HPLC, OmniSolv), acetone (ACS, Fisher), methanol (ACS, Fisher), ethanol (200 proof, Decon), and all other reagents were purchased commercially and used as received unless otherwise stated. Test tubes for frontal polymerizations were Fisherbrand 6x50 mm (inner diameter of ~4.75 mm, outer diameter ~6 mm) flint glass culture tubes and U-molds were prepared from 3 mm thick rubber gaskets using plain glass slides (25 x 75 x 1 mm).

Instrumentation:

Nuclear Magnetic Resonance (NMR):

Nuclear Magnetic Resonance (NMR) was performed on a Bruker 500 MHz using $CDCl_3$ as a solvent. NMR spectra were analyzed using MestreNova software.

Front Velocity Measurements:

Frontal polymerization was captured using a DSLR canon EOS R5 camera. The open-source physics (OSP) software package Tracker® was used to track the front location and calculate the average velocity.

Differential Scanning Calorimetry (DSC):

Differential scanning calorimetry (DSC) experiments were performed on a TA instruments Discovery DSC 250 instrument under nitrogen and analyzed using TA instruments Trios software. Cure kinetics were performed using 2-4 µL of reaction solution in sealed aluminum hermetic pans from 0 °C to 250 °C at a ramp rate of 10 °C min^{-1} and enthalpy was calculated by integrating area under the curve between 25 °C to 200 °C to include both exothermic peaks. The exotherm values were obtained from integrating the peaks. Post cure experiments were run using 5-15 mg of sample in sealed aluminum pans from 0 °C to 250 °C at a ramp rate of 10 °C min^{-1}. Once at 250 °C, a 1 min isotherm was performed before the sample was cooled at 10 °C min^{-1} to 0 °C. The heating and cooling cycles were performed a total of 2 times. Glass transition temperatures were obtained as the mid-point in the depression and averaged over three separate samples.

Size-Exclusion Chromatography (SEC):

Size-exclusion chromatography (SEC) was performed on a Tosoh EcoSEC Elite equipped with temperature controlled dual pumps, 100 vial auto sampler, automated purge unit and degasser, column oven, 4.6x3.5 cm TSKgel guard column, 6x15 cm TSKgel SuperH-RC reference column, and 7.8x30 cm TSKgel GMH_{hr}-M in THF at 40 °C and a flow rate of 1 mL min^{-1} and 0.5 mL min^{-1} THF for the sample column and reference column, respectively. The system is equipped with temperature controlled refractive index, UV-8420 Detector, and LenS3 Multi-Angle Light Scattering Detector with measurement angles fixed at 10°, 90°, and 170°. Absolute molecular weight and dispersity (*Đ*) values were determined by multiangle light scattering from purified polymers refractive index increment (dn/dc) values assuming 100% mass recovery.

Density Measurements:

Density of polymer materials were determined using Mettler Toledo XPR105DR analytical balance coupled with a Mettler Toledo Archimedes kit using ethanol (**Figure S155**).

Dynamic Mechanical Analysis (DMA):

Dynamic mechanical analysis (DMA) was used to evaluate the thermomechanical properties of post-FROMP specimens. DMA experiments were performed on a TA Instruments Discovery DMA850 with a -90 °C chiller using supplied tensile grips under an inert atmosphere. Temperature ramp experiments were performed from 0 to 180 °C at a rate of 3 °C min^{-1} with a frequency of 1 Hz, an oscillating strain of 0.05%, and a preload force of 0.01 N. All experiments were analyzed using TA instruments Trios software.

Tensile Testing:

Tensile testing was performed to evaluate the mechanical properties of the post-FROMP specimens. Tensile experiments were performed on a ZwickRoelle Retroline load frame equipped with a 5 kN XforceP load cell, videoXtens 2-150 HP video extensometer, and 5 kN over-wedge grips (type 9304.03). All samples were prepared in a gasket U-mold with a 3 mm thickness at 40 °C in a vacuum oven. Dogbone geometries were punched out using a Qualitest die (D-638-5-IMP-ASTM). Specimens were run with a starting preload force of 0.01 MPa and 5 mm/min at room temperature (~20 °C). Hysteresis testing was run with a starting preload force of 5 N and 5 mm/min at room temperature to 400% strain and cycled at the same rate for six total cycles followed by strain to failure on cycle 7. Increased strain rate experiments were run with a starting preload force of 0.01 MPa and 50 or 200 mm/min at room temperature until failure. Recording of tensile experiments was performed using a DSLR canon EOS R5 camera on a camera stand.

Experimental Procedures:

Synthesis of Hydrogenated Dicyclopentadiene (DCPD-H_2): Procedure Adapted from literature.[1-3]

Hydrobromination of Dicyclopentadiene (Norbornene Protection):

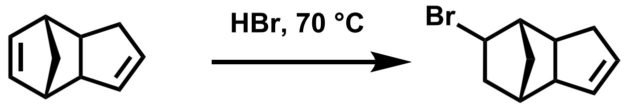

Dicyclopentadiene (DCPD; 326 g, 247 mmol, 1.00 equiv) was added to a 2L round-bottomed flask. 500 mL of hydrobromic acid (HBr; 48% w/w; 518 mmol, 2.10 equiv) was added to the reaction vessel, a stir bar equipped, and the flask was sealed. The reaction flask was purged with N_2 for approximately 10 min. Then, the flask was placed in a 70 °C preheated oil bath and stirred for 22 h. The reaction mixture was then diluted with 1 L of deionized water and added to a 2 L separatory funnel. The aqueous mixture washed with diethyl ether (4 x 250 mL), and the combined organic layers were subsequently washed with saturated sodium bicarbonate (3 x 100 mL) and brine (2 x 100 mL). The organic layer was collected, dried with sodium sulfate, and concentrated via rotary evaporation. The crude oil was allowed to dry overnight under reduced pressure. The crude product was then vacuum distilled (600 mtorr, 125 °C) yielding a red, clear oil (476 g, 90.5% yield). **Preliminary optimization for subsequent steps were performed on the initial orange colored*

DCPD-Br; however, a silica plug (100% hexanes mobile phase) should be performed on the distilled product prior to catalytic hydrogenation of DCPD-Br.

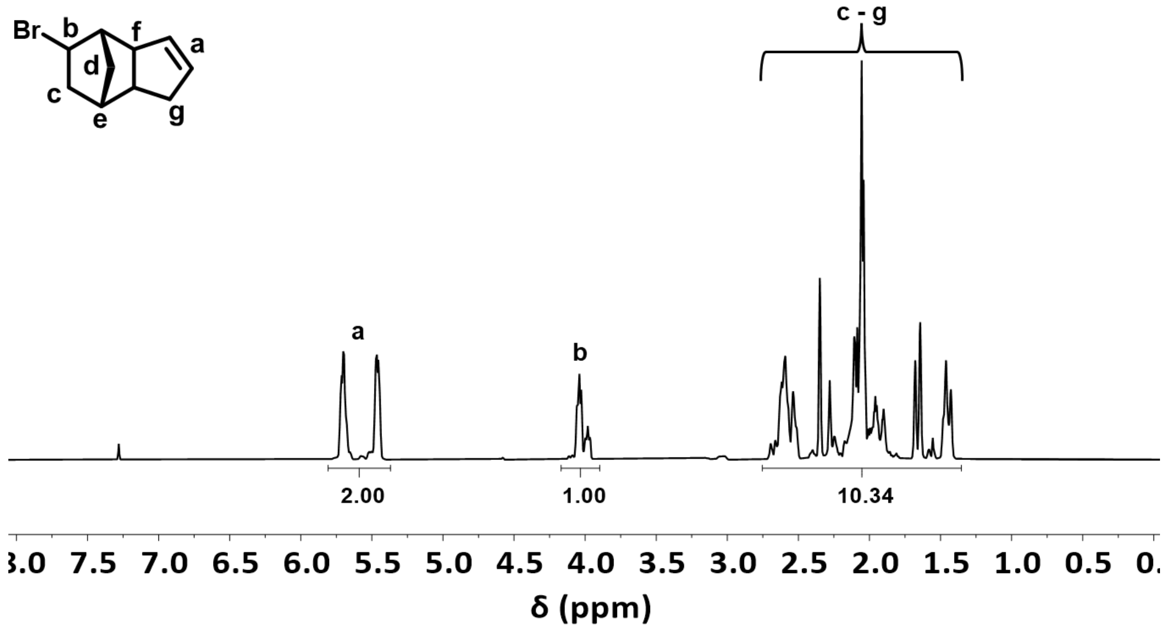

Figure S1: ^{1}H-NMR of bromo dicyclopentadiene (Br-DCPD).

Hydrogenation of Bromo-dicyclopentadiene (Br-DCPD):

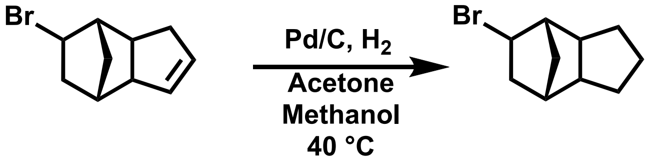

[300 g scale] Hydrogenation of Br-DCPD in 2:1 acetone/methanol (**with** silica purification)

Br-DCPD (315 g, 1.48 mol) was added to a 500 mL round-bottomed flask and dispersed in 375 mL of 2:1 acetone/methanol solvent mixture. 10% Pd/C (6.94 g) was added to the Br-DCPD solution, a stir bar was equipped, and the reaction vessel was sealed. While stirring, the reaction vessel was purged with N_2 for 1 min followed by vacuum until the solvent began to bubble (repeated 5 times). Next, four triple-wrapped balloons filled with H_2 were added and the reaction vessel was added to a 40 °C preheated oil bath for 96 h with H_2 balloons backfilled every 12 h. After 96 h, the slurry was vacuum eluted through a celite/silica plug and the plug was washed with acetone (8 x 50 mL). The filtrate was collected, transferred to a round-bottomed flask, and concentrated via rotary evaporation. The crude product was then purified with a silica plug (100% hexanes mobile phase). The eluent was concentrated via rotary evaporation and dried overnight under reduced pressure. ^{1}H NMR analysis indicated 99.0% conversion to the reduced product following one round of hydrogenation (**Figure S7**). The isolated Br-DCPD-H_2 was resubjected to a second round of catalytic hydrogenation using the same volume of 2:1 acetone/methanol but a reduced amount of 10% Pd/C (3.210 g). Four triple-wrapped balloons filled with H_2 were added and the reaction vessel was added to a 40 °C preheated oil bath for 60 h with H_2 balloons

backfilled every 12 h. Following and identical work-up and purification, ^{1}H NMR analysis indicated 100% conversion to the reduced product following the second round of hydrogenation (302 g, 95.1% yield) (**Figure S2**).

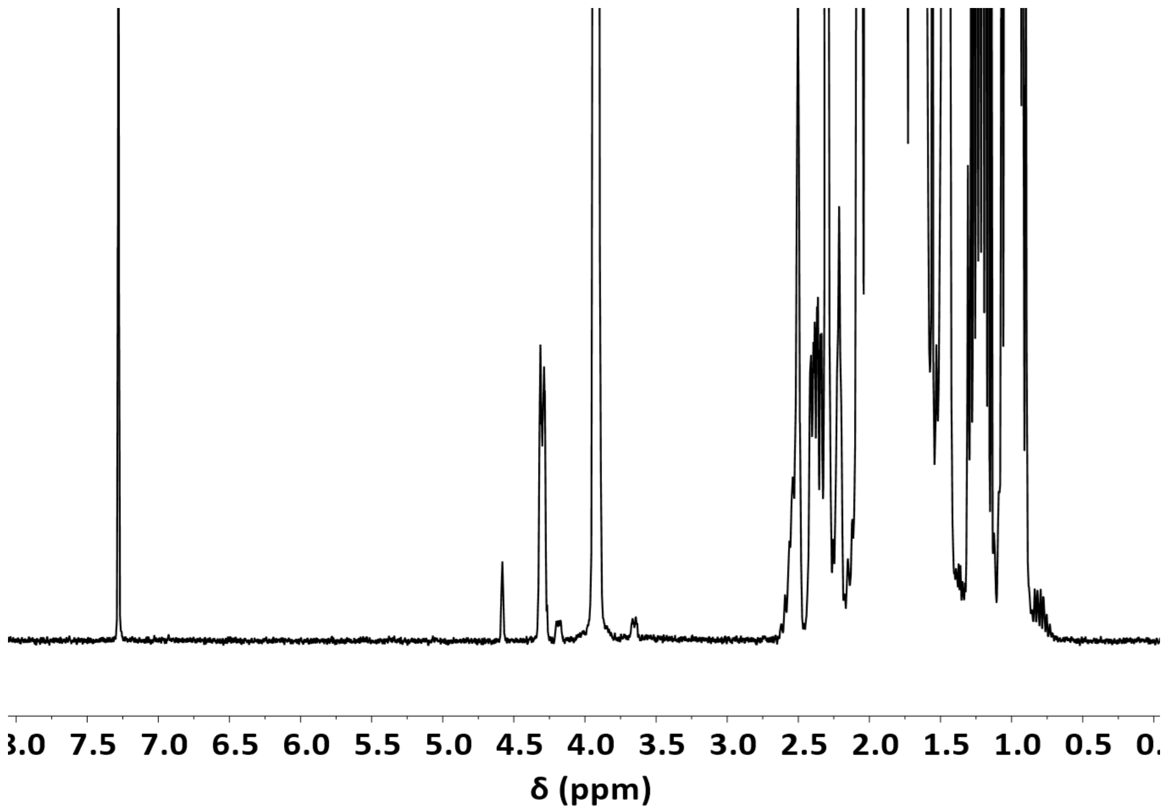

Figure S2: ^{1}H-NMR of quantitative hydrogenation of bromo-dicyclopentadiene (Br-DCPD-H_2) performed at a 300 g scale following a second round of catalytic hydrogenation in 2:1 acetone/methanol and prior silica plug purification.

Elimination of Hydrogenated Bromo-dicyclopentadiene (Br-DCPD-H_2):

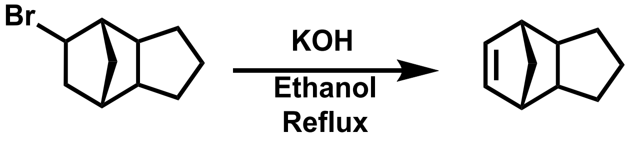

Hydrogenated bromo-dicyclopentadiene (Br-DCPD-H_2; 302 g, 1.41 mol, 1.00 equiv) in a 2 L round-bottomed flask was dispersed in 900 mL of absolute (200 proof) ethanol. Next, potassium hydroxide pellets (206 g, 3.65 mol, 2.60 equiv) was added to the Br-DCPD-H_2 solution. A stir bar and reflux condenser was equipped, and the reaction mixture was stirred in a 100 °C preheated oil bath for 36 h. The reaction vessel was then allowed to cool to room temperature and was diluted with 1 L of deionized water. In two separate batches, the diluted solution was added to a 2 L separatory funnel and extracted with diethyl ether (3 x 300 mL). The two separate organic washes were combined, added to the separatory funnel, and washed with deionized water (2 x 500 mL) and brine (2 x 500 mL). The organic layer was collected, dried with sodium sulfate,

concentrated via rotary evaporation, and dried overnight under reduced pressure to give a clear, bright yellow oil. This crude product was vacuum distilled (40 °C, 600 mtorr) yielding a clear, colorless oil (158 g, 84.0% yield).

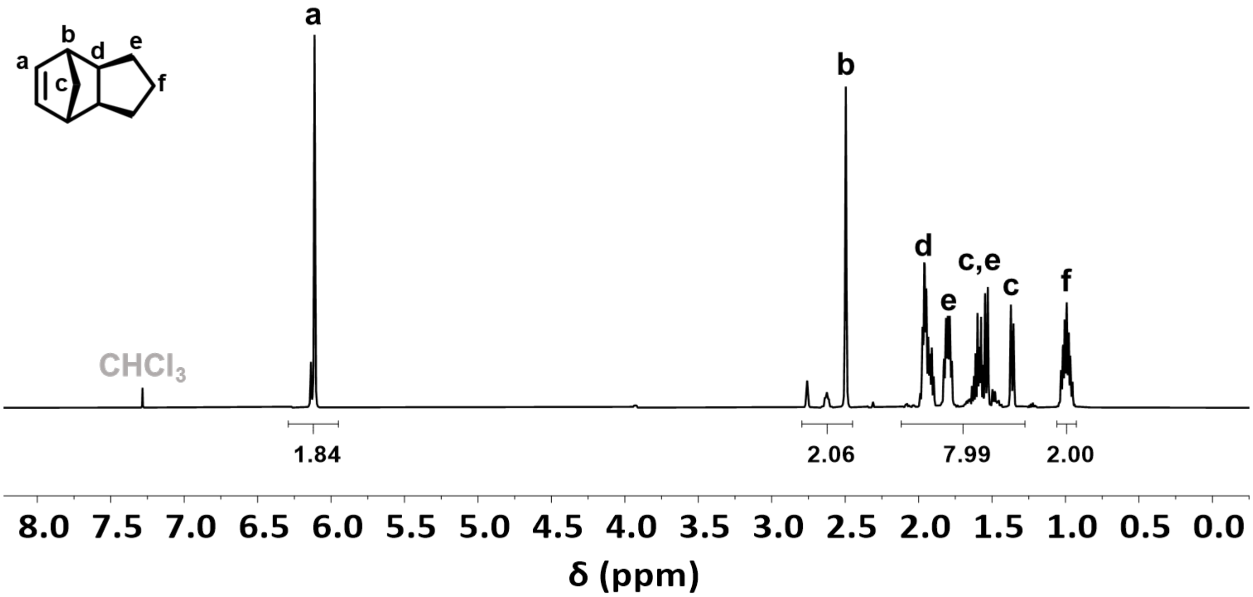

Figure S3: ^{1}H-NMR of hydrogenated dicyclopentadiene (DCPD-H_2).

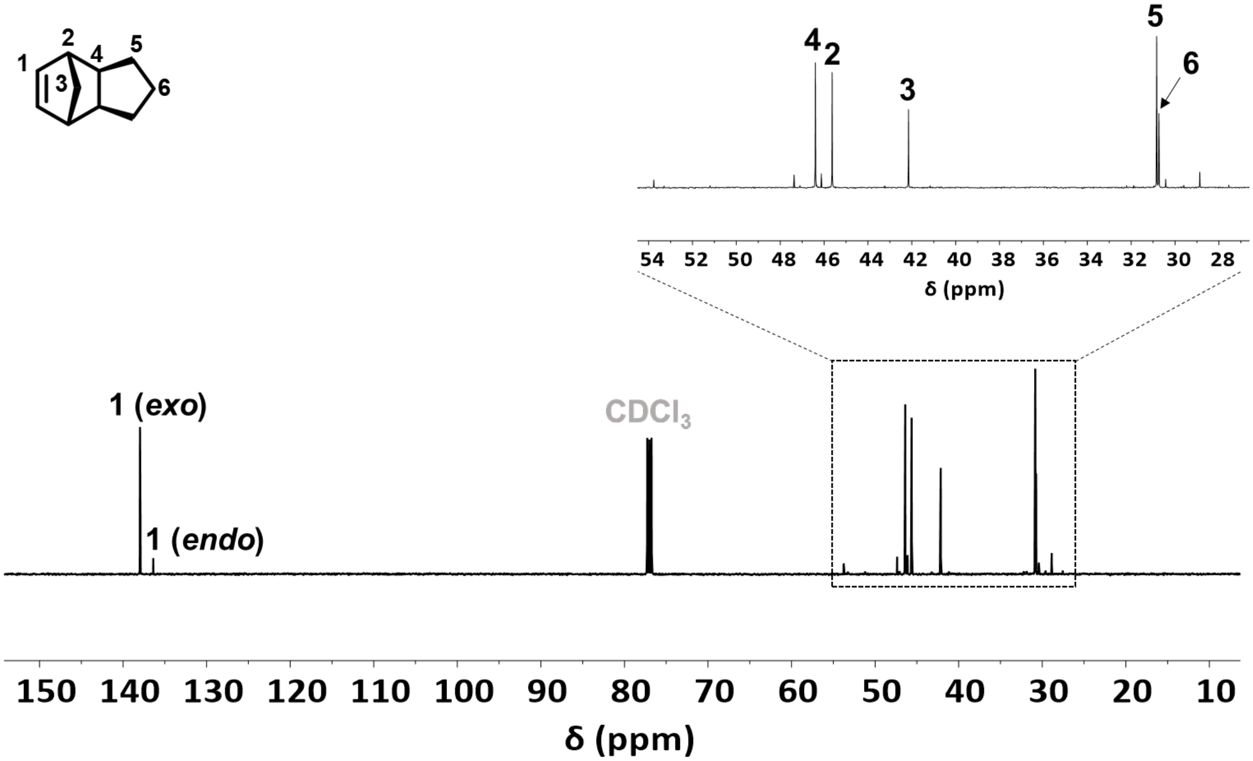

Figure S4: ^{13}C-NMR of hydrogenated dicyclopentadiene (DCPD-H_2).

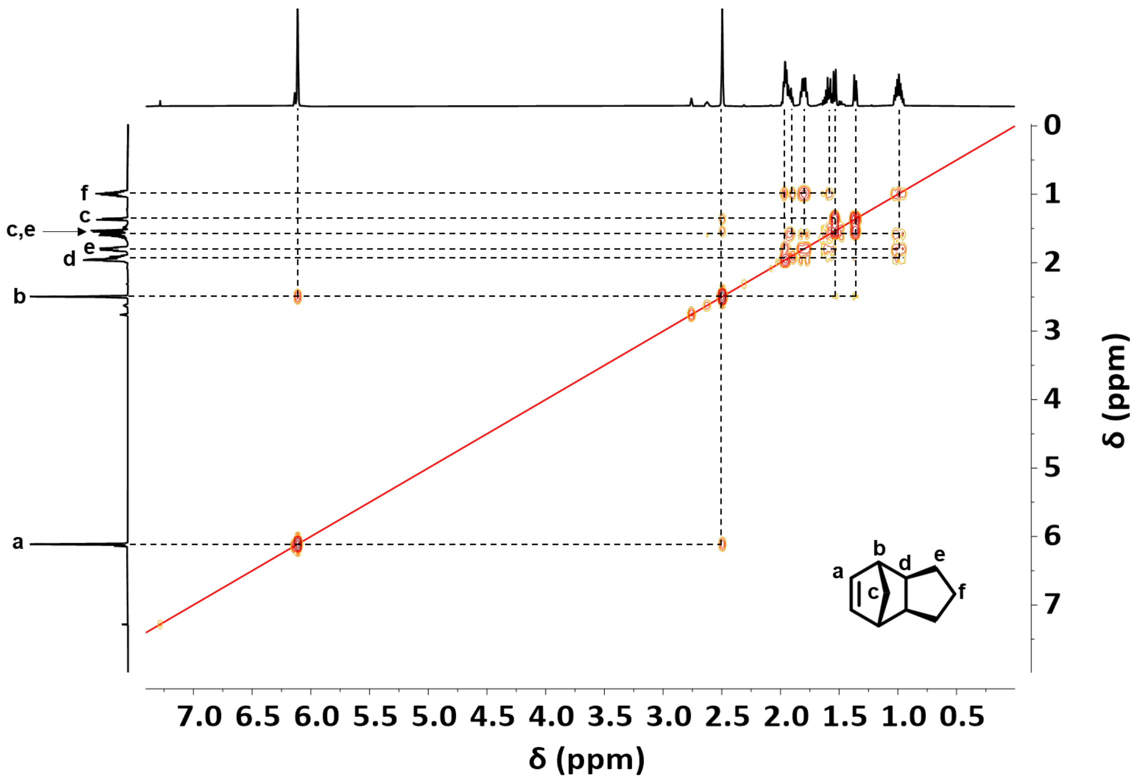

Figure S5: COSY NMR of hydrogenated dicyclopentadiene (DCPD-H_2).

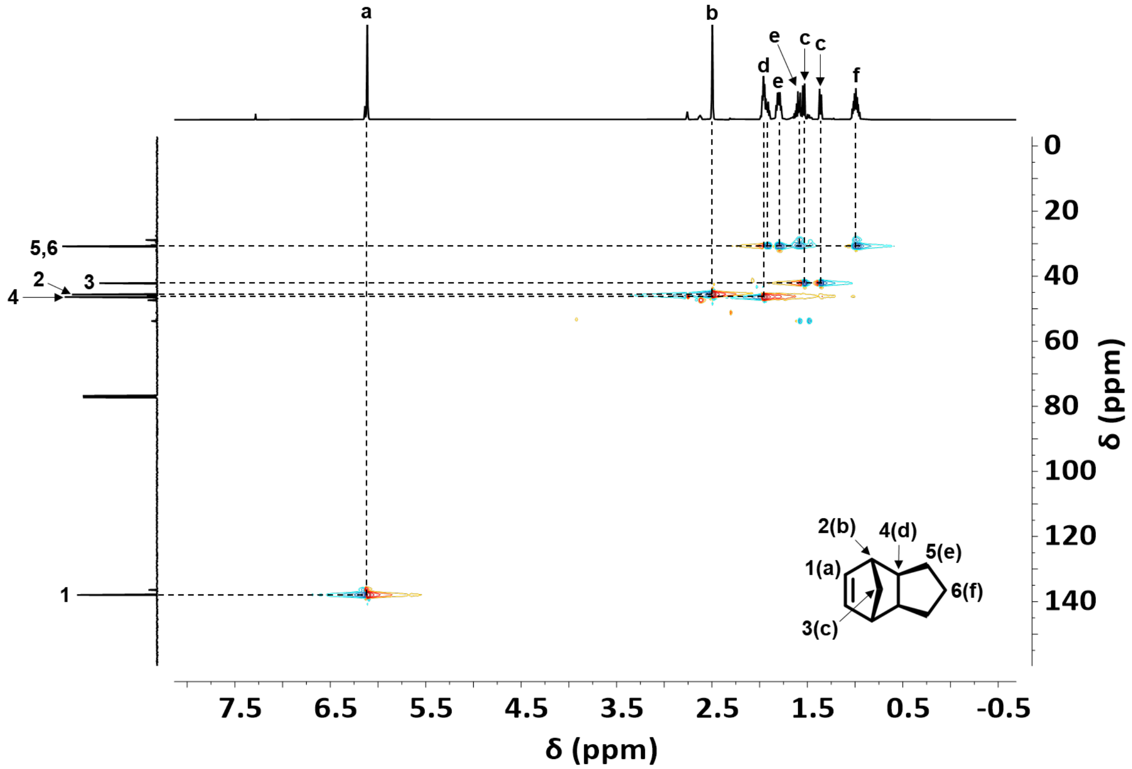

Figure S6: HSQC NMR of hydrogenated dicyclopentadiene (DCPD-H_2).

Steiglitz Esterification of 5-Norbornene-2-carboxylic acid to 5-Norbornene-2-octylester (NBE8):

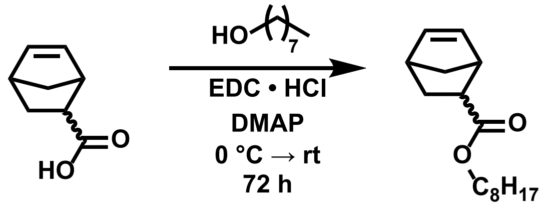

1-Octanol (31.4 mL, 199 mmol, 1.10 equiv) was added to an oven dried 500 mL round-bottomed flask and dispersed in 180 mL of anhydrous dichloromethane. Next, 5-norbornene-2-carboxylic acid (NB-COOH, 25.0 g, 181 mmol, 1.00 equiv) was massed in two portions of approximately 12.5 g in separate 20 mL scintillation vials. Then, 5 mL of anhydrous dichloromethane (DCM) was used to dissolve the NB-COOH, and the solution was added to the 500 mL reaction vessel. The vials were washed with an additional 5 mL of DCM and added to the reaction vessel. Next, dimethylaminopyridine (2.21 g, 18.1 mmol, 0.100 equiv) was added to the reaction vessel. Under an N_2 blanket and stirring on ice, (3-Dimethylamino-propyl)-ethyl-carbodiimide hydrochloride (EDC•HCl, 38.2 g, 199 mmol, 1.10 equiv) was added portionwise over approximately 15 minutes. Following full addition of EDC•HCl, the vessel was sealed with a rubber septum and purged with N_2 for 5 minutes. The vessel was then removed from ice and allowed to stir at room temperature for 72 h. After 72 h, the DCM was removed via rotary evaporation leaving an orange-yellow biphasic residue. The crude heterogenous mixture was placed in a -20 °C freezer overnight. Next, the top layer (crude product) was decanted from the bottom layer (EDC•HCl byproduct). The remaining residue was washed with approximately 50 mL of hexanes, followed by decantation of the hexanes solution. The crude residue and hexanes wash were added to a silica plug and the product was eluted with a 100% hexanes mobile phase. The eluent was collected until minimal product evolution was observed by thin-layer chromatography. The eluent was concentrated via rotary evaporation and dried overnight under reduced pressure, yielding a clear, colorless oil (34.1 g, 75.2% yield).

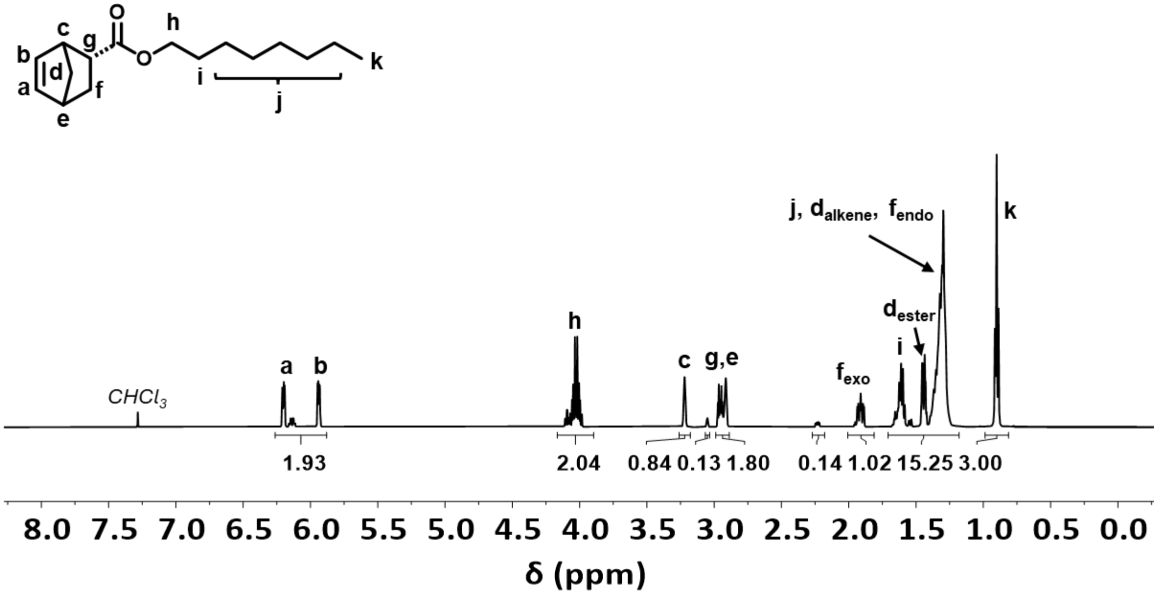

Figure S7: ^{1}H NMR of 5-Norbornene-2-octylester (NBE8).

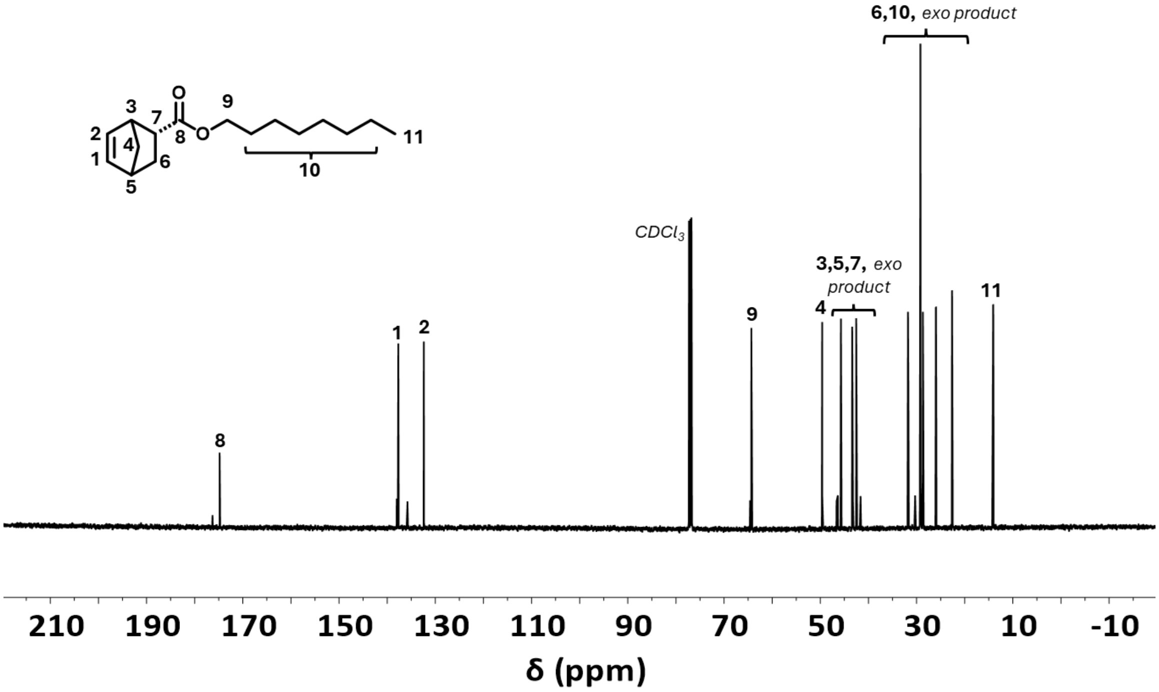

Figure S8: ^{13}C NMR of 5-Norbornene-2-octylester (NBE8).

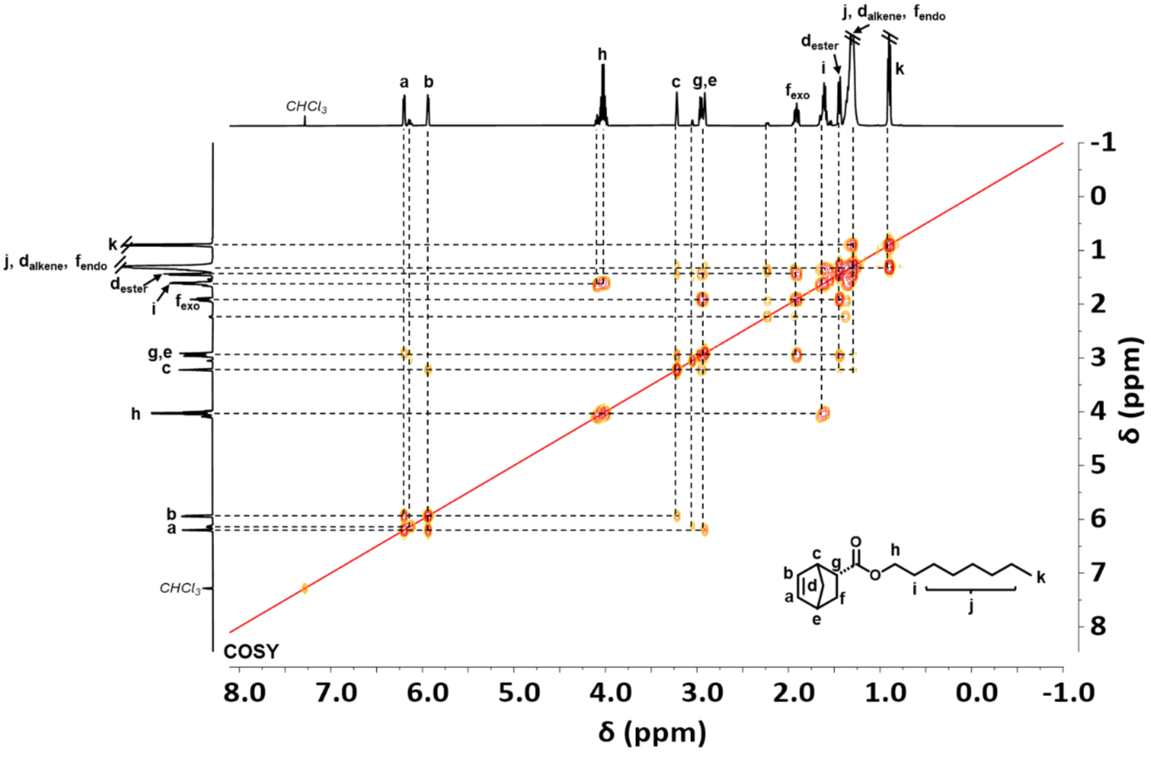

Figure S9: COSY NMR of 5-Norbornene-2-octylester (NBE8).

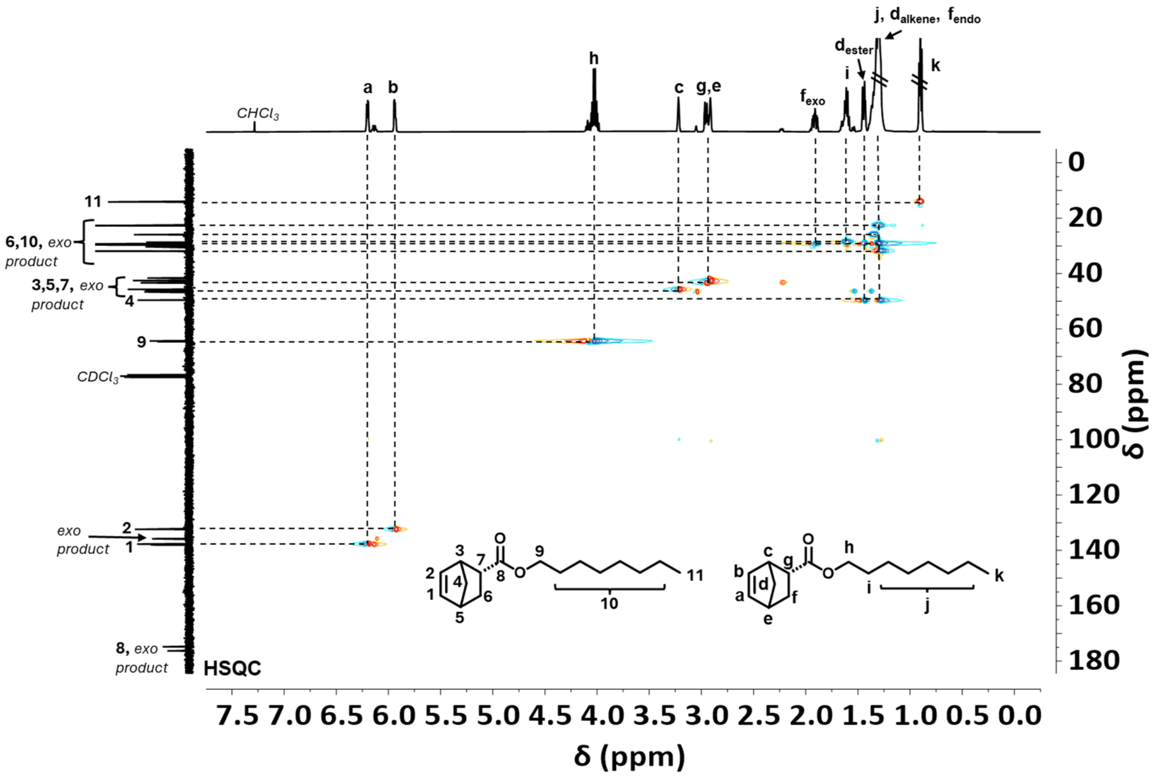

Figure S10: HSQC NMR of 5-Norbornene-2-octylester (NBE8).

<u>5-Norbornene-2-dodecylester (NBE12):</u>

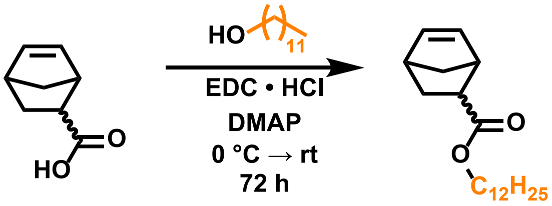

1-Dodecanol (37.4 mL, 167 mmol, 1.10 equiv) was added to an oven dried 500 mL round-bottomed flask and dispersed in 180 mL of anhydrous dichloromethane (DCM). Next, 5-norbornene-2-carboxylic acid (NB-COOH, 21.0 g, 152 mmol, 1.00 equiv) was massed in two portions of approximately 10.5 g in separate 20 mL scintillation vials. Then, 5 mL of anhydrous DCM was used to dissolve the NB-COOH, and the solution was added to the 500 mL reaction vessel. The vials were washed with an additional 5 mL of DCM and added to the reaction vessel. Next, dimethylaminopyridine (1.86 g, 15.2 mmol, 0.100 equiv) was added to the reaction vessel. Under an N_2 blanket and stirring on ice, (3-Dimethylamino-propyl)-ethyl-carbodiimide hydrochloride (EDC•HCl, 32.1 g, 167 mmol, 1.10 equiv) was added portionwise over approximately 15 minutes. Following full addition of EDC•HCl, the vessel was sealed with a rubber septum and purged with N_2 for 5 minutes. The vessel was then removed from ice and allowed to stir at room temperature for 72 h. After 72 h, the DCM was removed via rotary evaporation leaving an orange-yellow biphasic residue. The crude heterogenous mixture was placed in a -20 °C freezer overnight. Next, the top layer (crude product) was allowed to melt and decanted from the bottom layer (EDC•HCl byproduct). The remaining residue was washed with approximately 50

mL of hexanes, followed by decantation of the hexanes solution. The crude residue and hexanes wash were added to a silica plug and the product was eluted with a 100% hexanes mobile phase. The eluent was then collected until minimal product evolution was observed by thin-layer chromatography. The eluent was concentrated via rotary evaporation and dried overnight under reduced pressure, yielding a clear, colorless oil (28.8 g, 61.7% yield).

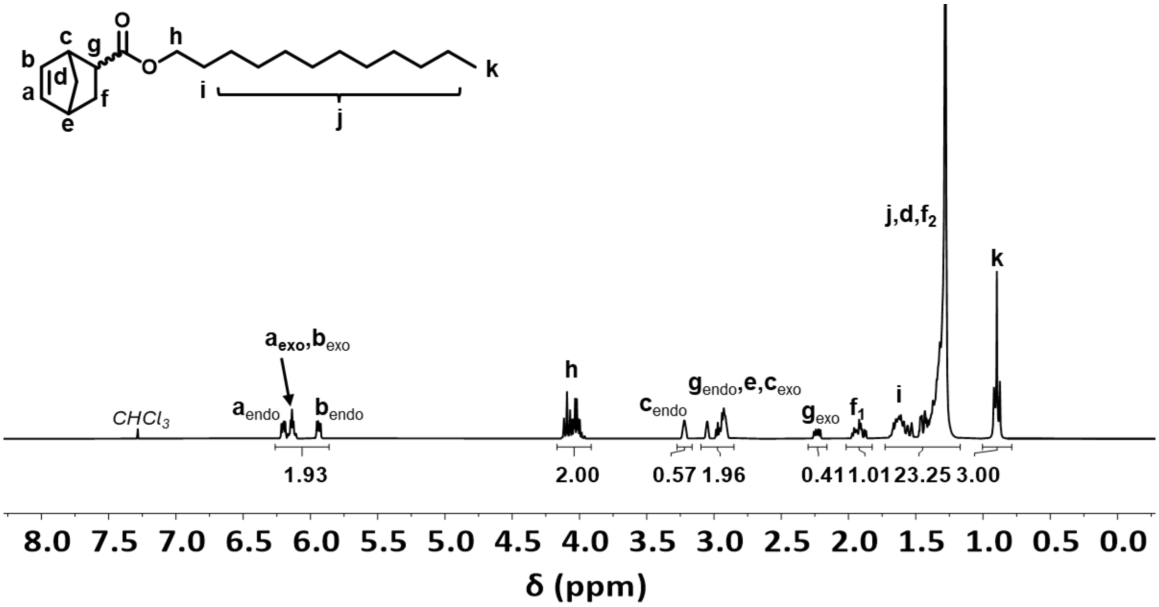

Figure S11: ^{1}H NMR of 5-Norbornene-2-dodecylester (NBE12).

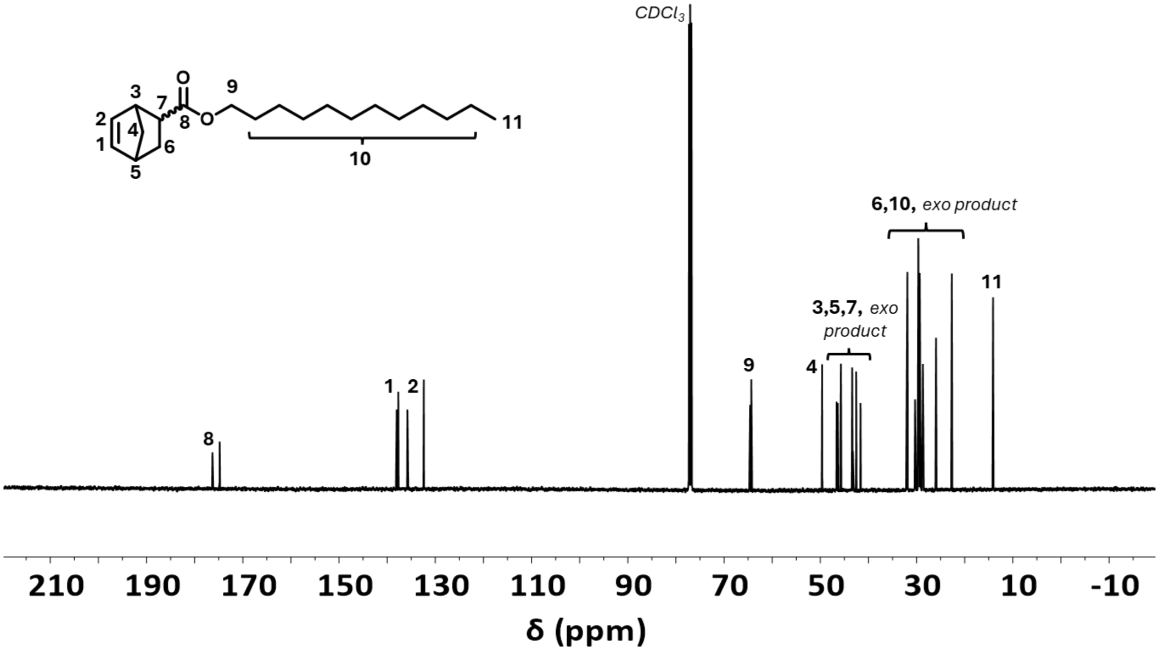

Figure S12: ^{13}C NMR of 5-Norbornene-2-dodecylester (NBE12).

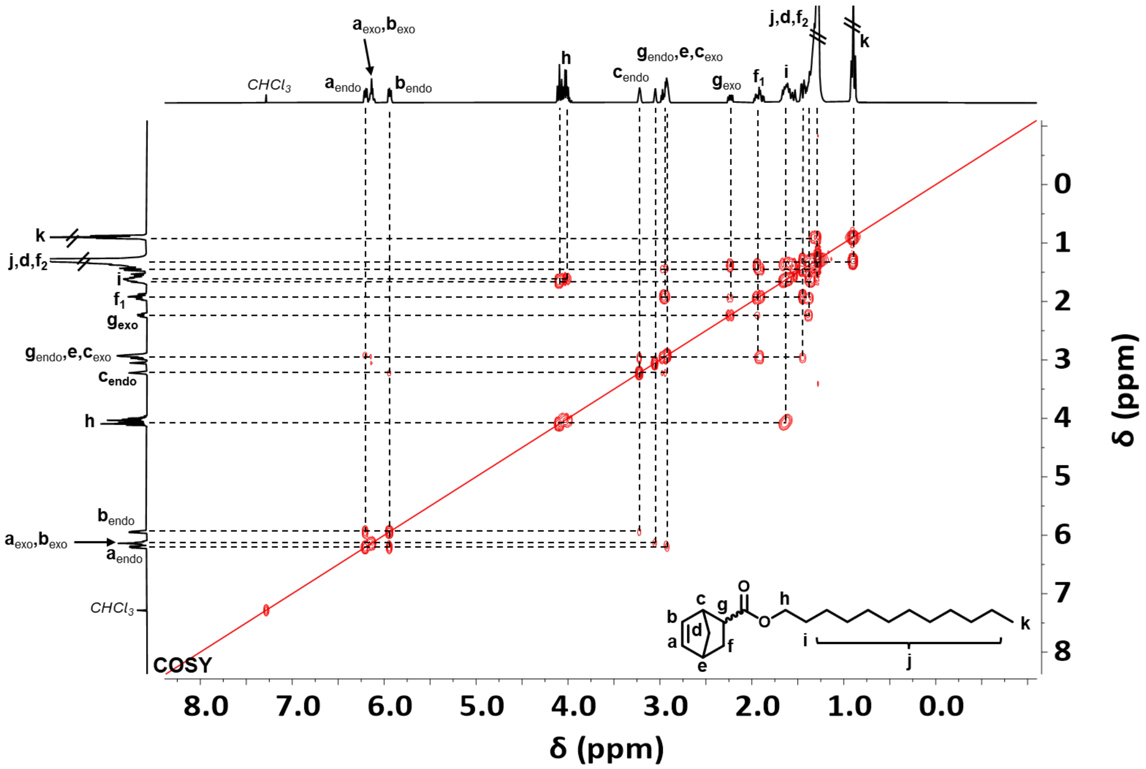

Figure S13: COSY NMR of 5-Norbornene-2-dodecylester (NBE12).

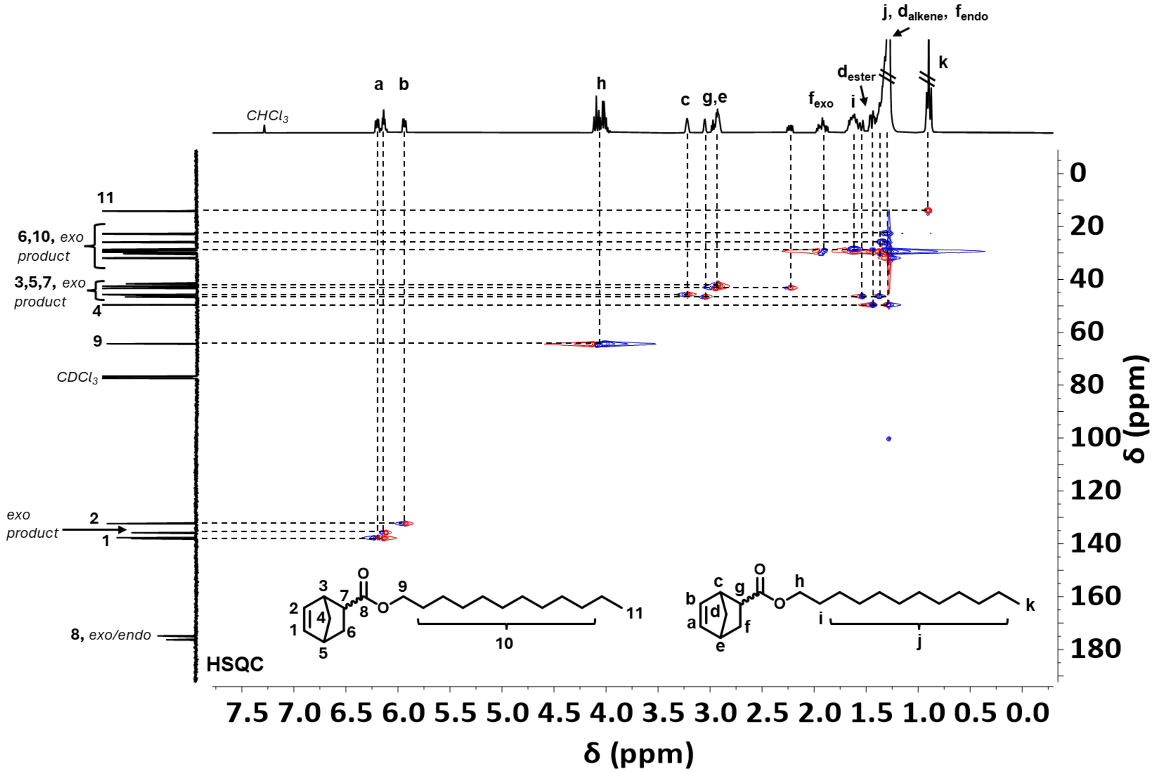

Figure S14: HSQC NMR of 5-Norbornene-2-dodecylester (NBE12).

5-Norbornene-2-hexadecylester (NBE16):

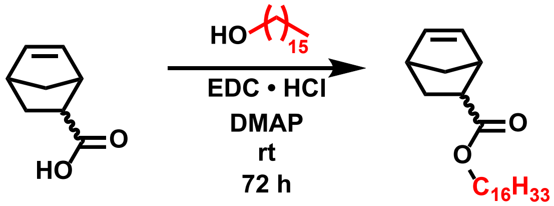

1-Hexadecanol (24.0 g, 99.0 mmol, 1.10 equiv) was added to a oven dried 500 mL round-bottomed flask and dispersed in 230 mL of anhydrous dichloromethane. Next, 5-norbornene-2-carboxylic acid (NB-COOH, 15.1 g, 109 mmol, 1.00 equiv) was massed in a 20 mL scintillation vial. Then, 5 mL of anhydrous dichloromethane (DCM) was used to dissolve the NB-COOH, and the solution was added to the 500 mL reaction vessel. The scintillation vials were washed with an additional 5 mL of DCM and added to the reaction vessel. Next, dimethylaminopyridine (1.33 g, 10.9 mmol, 0.100 equiv) was added to the reaction vessel. Under an N_2 blanket and stirring at room temperature [**1-hexadecanol will begin to recrystallize in an ice bath**], (3-Dimethylamino-propyl)-ethyl-carbodiimide hydrochloride (EDC•HCl, 20.9 g, 109 mmol, 1.10 equiv) was added portionwise over approximately 15 minutes. Following full addition of EDC•HCl, the vessel was sealed with a rubber septum and purged with N_2 for 5 minutes. The vessel was allowed to stir at room temperature for 72 h. After 72 h, the DCM was removed via rotary evaporation leaving an orange-yellow biphasic residue. The crude heterogenous mixture was placed in a -20 °C overnight. Next, the mixture was allowed to come to room temperature to allow melting of the crude, crystalline top layer. Upon melting, the top layer (crude product) was decanted from the bottom layer (EDC•HCl byproduct). The remaining residue was washed with approximately 50 mL of hexanes, followed by decantation of the hexanes solution. The crude residue and hexanes wash were added to a silica plug and the product was eluted with a 100% hexanes mobile phase. The eluent was then collected until minimal product evolution was observed thin-layer chromatography. The eluent was concentrated via rotary evaporation and dried over two nights under reduced pressure and light heating (40 °C), yielding a clear, colorless oil (27.9 g, 70.6% yield).

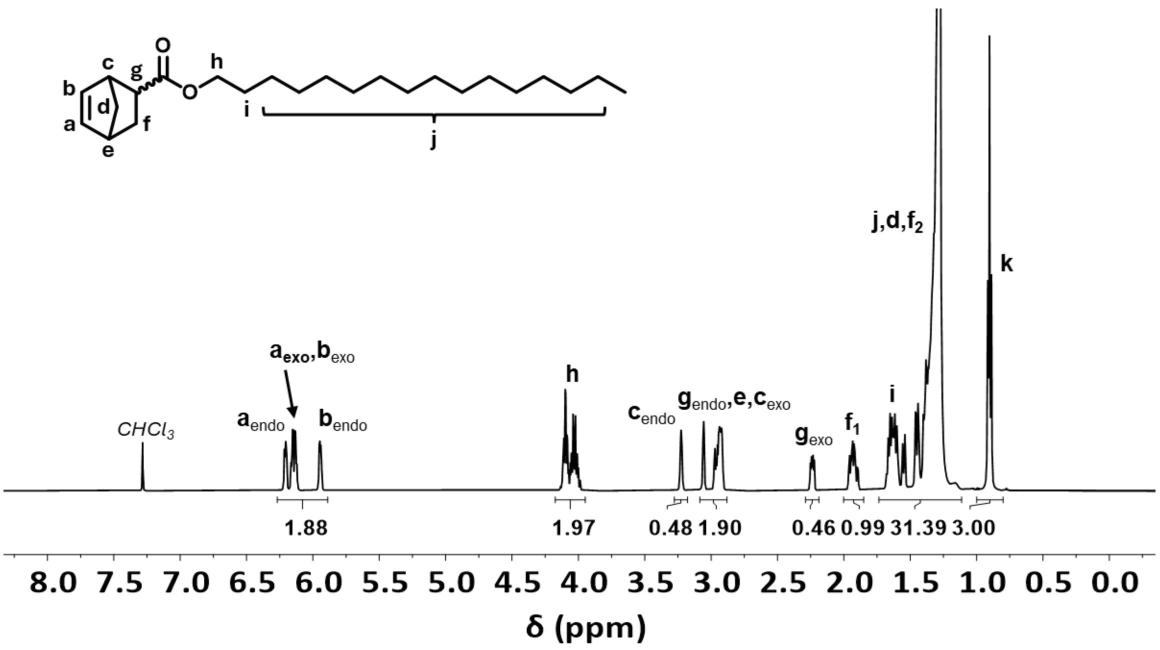

Figure S15: ^{1}H NMR of 5-Norbornene-2-hexadecylester (NBE16).

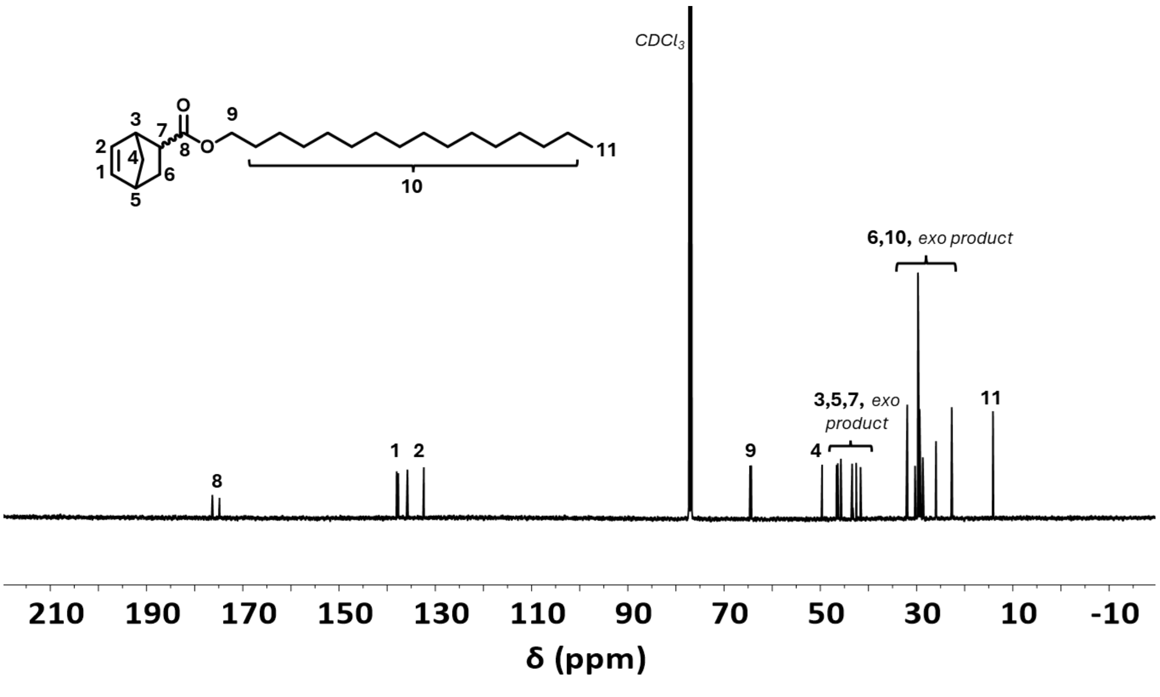

Figure S16: ^{13}C NMR of 5-Norbornene-2-hexadecylester (NBE16).

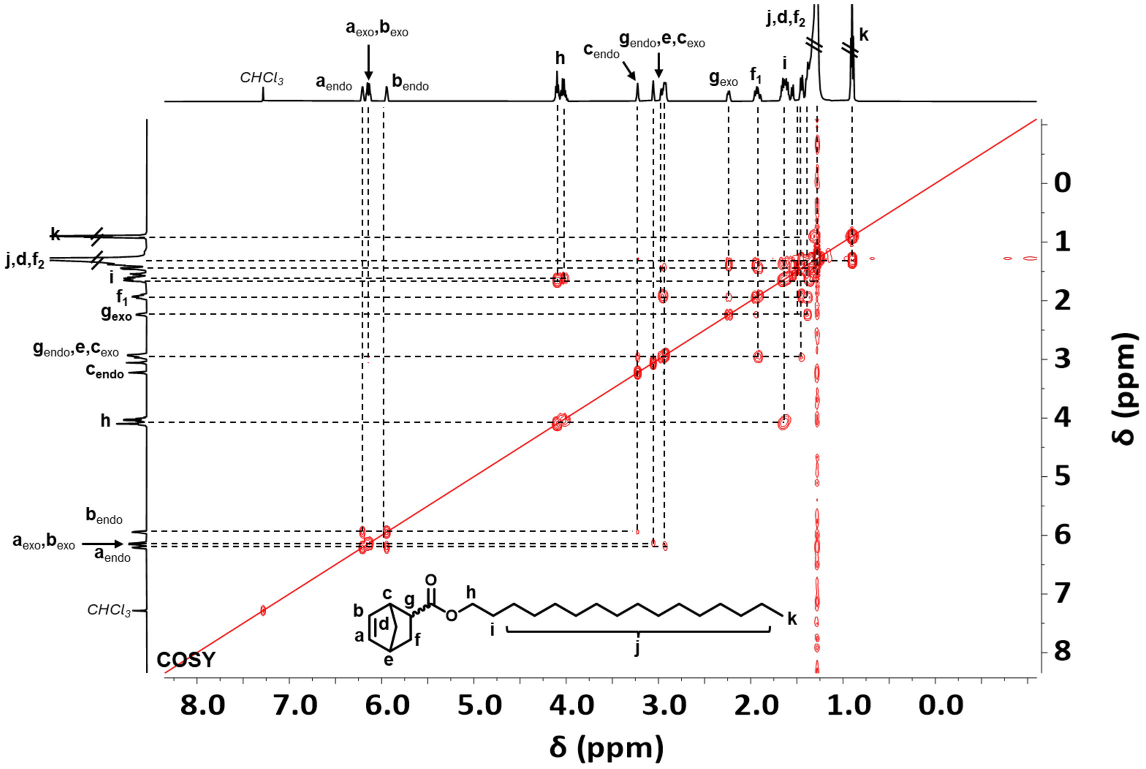

Figure S17: COSY NMR of 5-Norbornene-2-hexadecylester (NBE16).

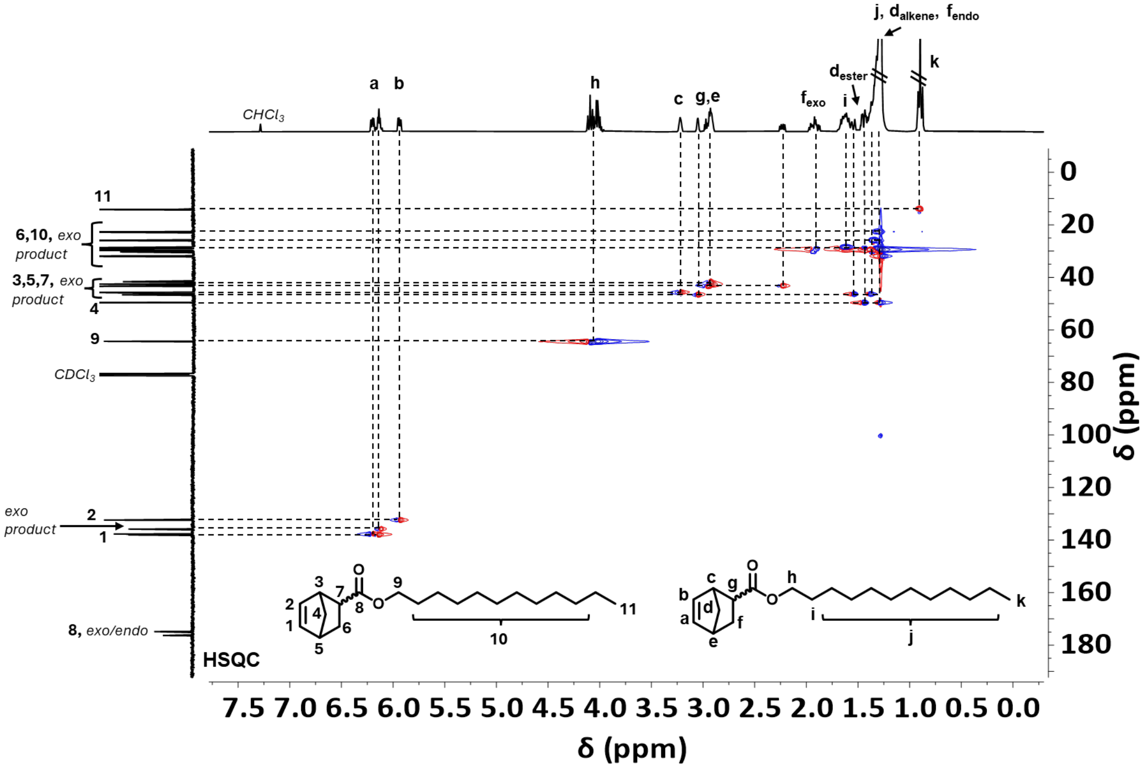

Figure S18: HSQC NMR of 5-Norbornene-2-hexadecylester (NBE16).

General Procedure for NBE and DCPD-H_2 Resin Preparation:

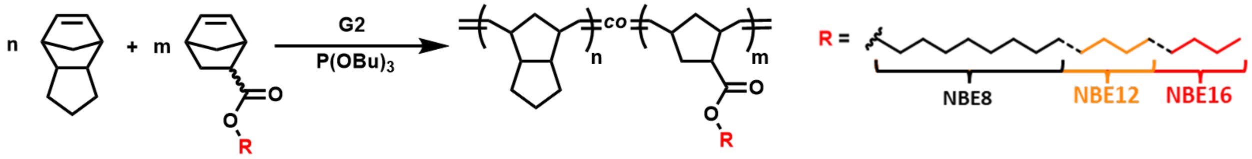

Grubbs' 2nd generation initiator was massed (G2, **w** mg, 1.00 equiv) in a 5 mL vial prior to the addition of tributyl phosphite (TBP, **x** μL, **y** equiv). The mixture was dissolved in monomer (*NBE and DCPD-H_2*, 500 mg, **z** equiv) and sonicated for up to 5 minutes. The resulting solution was transferred into a test tube. Test tube samples were initiated at the top of the resin using a preheated soldering iron creating a descending front.

<u>50 mol% NBE8</u>

<u>1000:1:1 Monomer:Initiator:Inhibitor</u>

G2: **w** = 2.21 mg

TBP: **x** = 0.70 μL, **y** = 1.00 equiv

NBE8 and DCPD-H_2: **z** = 500 and 500 equiv, respectively

<u>500:1:1 Monomer:Initiator:Inhibitor</u>

G2: **w** = 4.41 mg

TBP: **x** = 1.40 μL, **y** = 1.00 equiv

NBE8 and DCPD-H_2: **z** = 250 and 250 equiv, respectively

<u>200:1:1 Monomer:Initiator:Inhibitor</u>

G2: **w** = 11.04 mg

TBP: **x** = 3.55 μL, **y** = 1.00 equiv

NBE8 and DCPD-H_2: **z** = 100 and 100 equiv, respectively

<u>25 mol% NBE8</u>

<u>4000:1:1 Monomer:Initiator:Inhibitor</u>

G2: **w** = 0.65 mg

TBP: **x** = 0.20 μL, **y** = 1.00 equiv

NBE8 and DCPD-H_2: **z** = 1000 and 3000 equiv, respectively

<u>2000:1:1 Monomer:Initiator:Inhibitor</u>

G2: **w** = 1.30 mg

TBP: **x** = 0.40 μL, **y** = 1.00 equiv

NBE8 and DCPD-H_2: **z** = 500 and 1500 equiv, respectively

1000:1:1 Monomer:Initiator:Inhibitor

G2: **w** = 2.60 mg

TBP: **x** = 0.80 µL, **y** = 1.00 equiv

NBE8 and DCPD-H_2: **z** = 250 and 750 equiv, respectively

500:1:1 Monomer:Initiator:Inhibitor

G2: **w** = 5.20 mg

TBP: **x** = 1.60 µL, **y** = 1.00 equiv

NBE8 and DCPD-H_2: **z** = 125 and 375 equiv, respectively

200:1:1 Monomer:Initiator:Inhibitor

G2: **w** = 13.00 mg

TBP: **x** = 4.20 µL, **y** = 1.00 equiv

NBE8 and DCPD-H_2: **z** = 50 and 150 equiv, respectively

25 mol% NBE12

4000:1:1 Monomer:Initiator:Inhibitor

G2: **w** = 0.60 mg

TBP: **x** = 0.20 µL, **y** = 1.00 equiv

NBE12 and DCPD-H_2: **z** = 1000 and 3000 equiv, respectively

2000:1:1 Monomer:Initiator:Inhibitor

G2: **w** = 1.20 mg

TBP: **x** = 0.40 µL, **y** = 1.00 equiv

NBE12 and DCPD-H_2: **z** = 500 and 1500 equiv, respectively

1000:1:1 Monomer:Initiator:Inhibitor

G2: **w** = 2.39 mg

TBP: **x** = 0.80 µL, **y** = 1.00 equiv

NBE12 and DCPD-H_2: **z** = 250 and 750 equiv, respectively

500:1:1 Monomer:Initiator:Inhibitor

G2: **w** = 4.79 mg

TBP: **x** = 1.55 µL, **y** = 1.00 equiv

NBE12 and DCPD-H_2: **z** = 125 and 375 equiv, respectively

200:1:1 Monomer:Initiator:Inhibitor

G2: **w** = 11.97 mg

TBP: **x** = 3.85 μL, **y** = 1.00 equiv

NBE12 and DCPD-H_2: **z** = 50 and 150 equiv, respectively

<u>25 mol% NBE16</u>

<u>4000:1:1 Monomer:Initiator:Inhibitor</u>

G2: **w** = 0.55 mg

TBP: **x** = 0.20 μL, **y** = 1.00 equiv

NBE16 and DCPD-H_2: **z** = 1000 and 3000 equiv, respectively

<u>2000:1:1 Monomer:Initiator:Inhibitor</u>

G2: **w** = 1.11 mg

TBP: **x** = 0.35 μL, **y** = 1.00 equiv

NBE16 and DCPD-H_2: **z** = 500 and 1500 equiv, respectively

<u>1000:1:1 Monomer:Initiator:Inhibitor</u>

G2: **w** = 2.22 mg

TBP: **x** = 0.70 μL, **y** = 1.00 equiv

NBE16 and DCPD-H_2: **z** = 250 and 750 equiv, respectively

<u>500:1:1 Monomer:Initiator:Inhibitor</u>

G2: **w** = 4.44 mg

TBP: **x** = 1.40 μL, **y** = 1.00 equiv

NBE16 and DCPD-H_2: **z** = 125 and 375 equiv, respectively

Timelapses of NBE Monomers in DCPD-H_2:

50 mol% NBE8

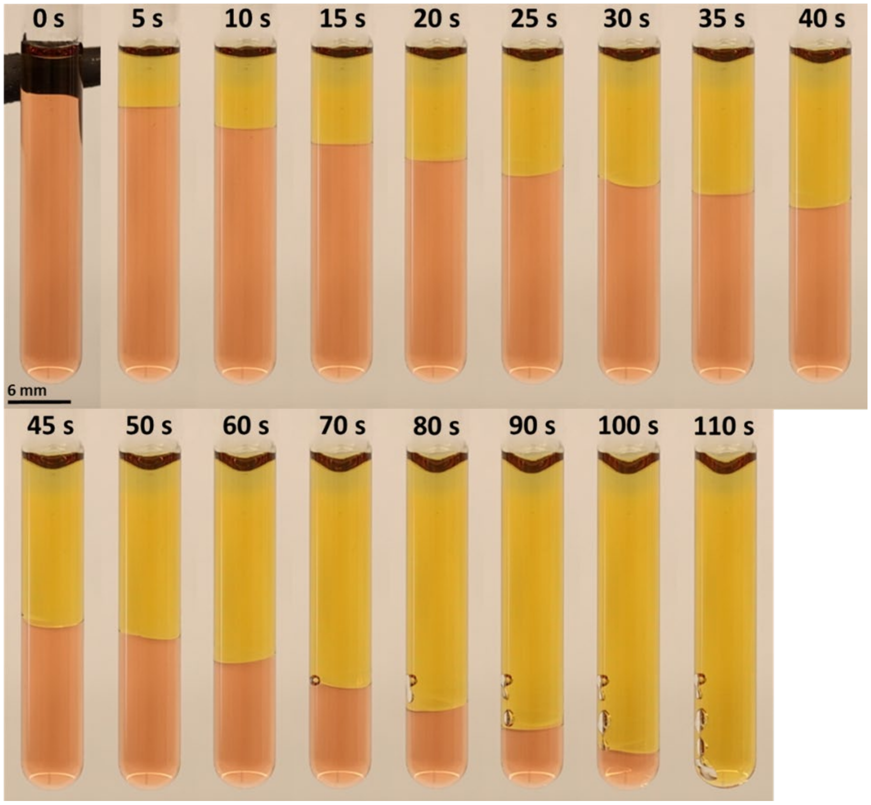

Figure S19: Representative timelapse of 1000:1:1 50 mol% NBE8 in DCPD-H_2:G2:TBP. The scale bar is 5 mm.

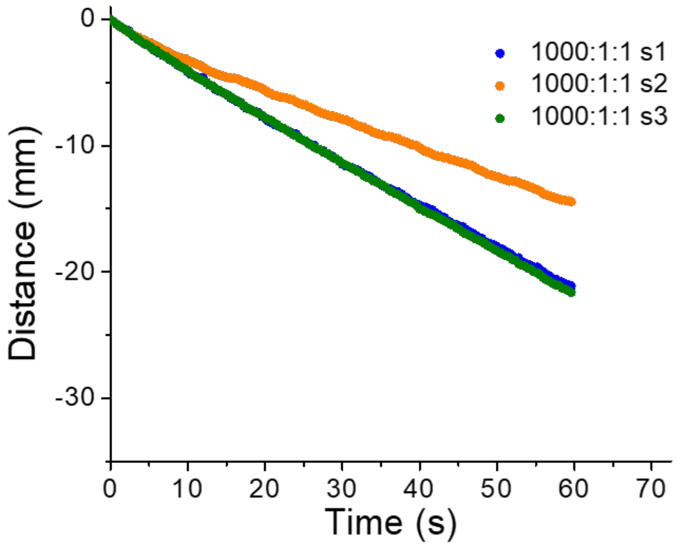

Figure S20: Front tracking of 1000:1:1 50 mol% NBE8 in DCPD-H_2:G2:TBP in triplicate (v_f = 0.31 ± 0.06 mm/s).

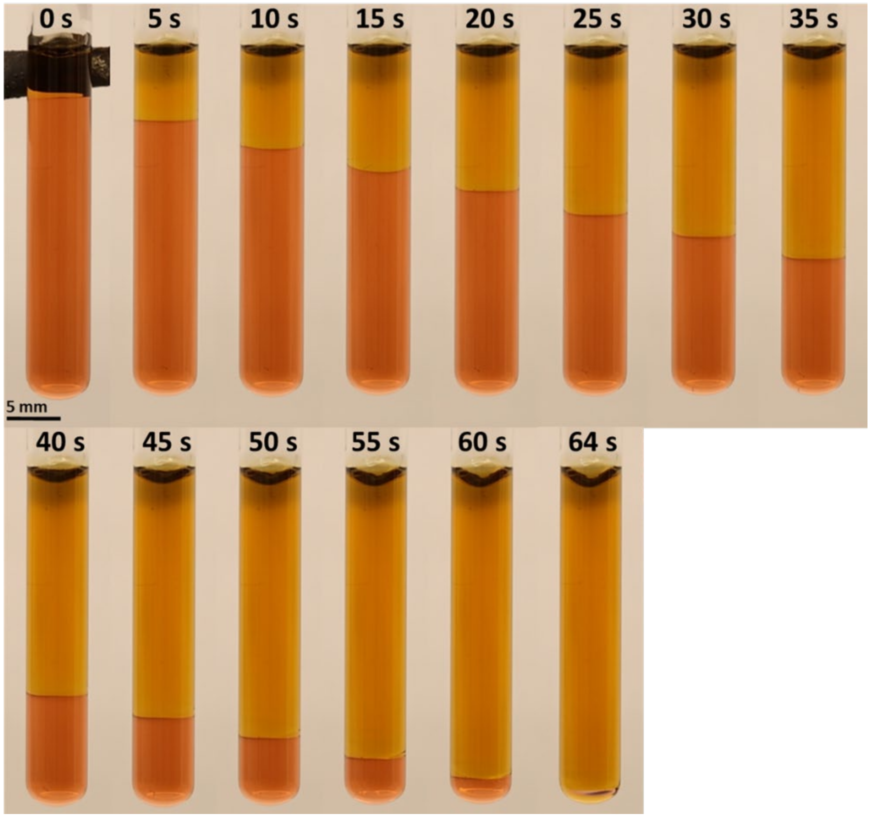

Figure S21: Representative timelapse of 500:1:1 50 mol% NBE8 in DCPD-H_2:G2:TBP. The scale bar is 5 mm.

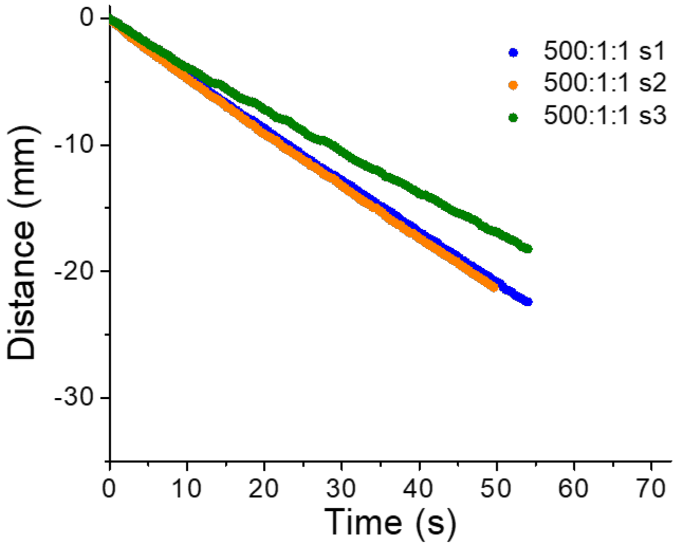

Figure S22: Front tracking of 500:1:1 50 mol% NBE8 in DCPD-H_2:G2:TBP in triplicate (v_f = 0.39 ± 0.04 mm/s).

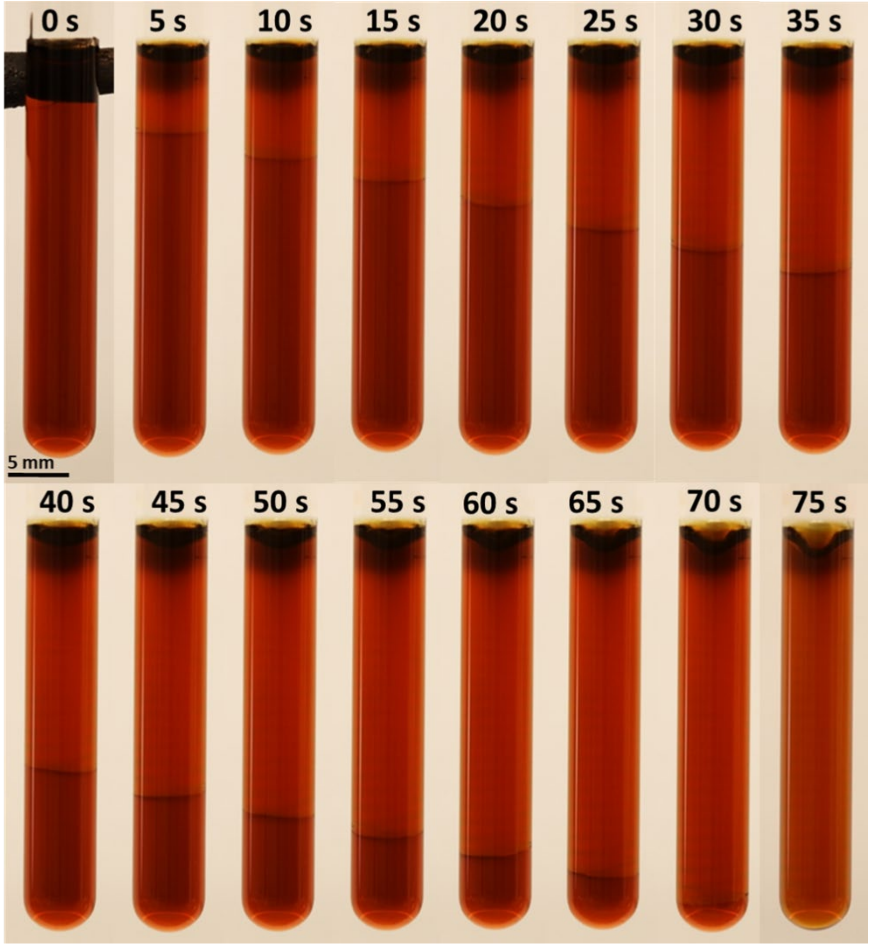

Figure S23: Representative timelapse of 200:1:1 50 mol% NBE8 in DCPD-H_2:G2:TBP. The scale bar is 5 mm.

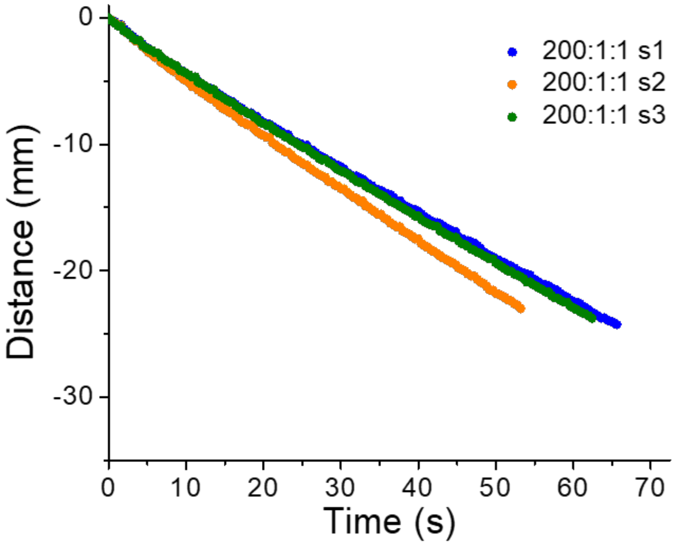

Figure S24: Front tracking of 200:1:1 50 mol% NBE8 in DCPD-H_2:G2:TBP in triplicate (v_f = 0.39 ± 0.03 mm/s).

25 mol% NBE8

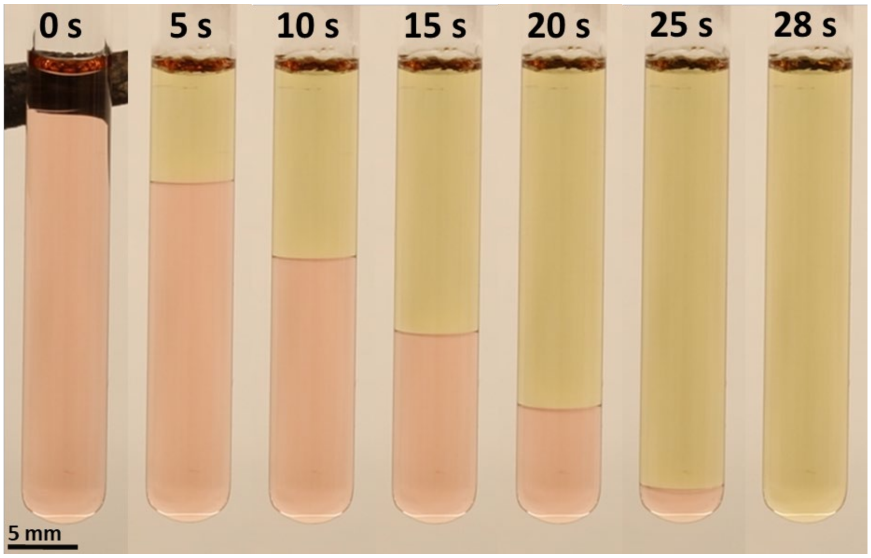

Figure S25: Representative timelapse of 4000:1:1 25 mol% NBE8 in DCPD-H_2:G2:TBP. The scale bar is 5 mm.

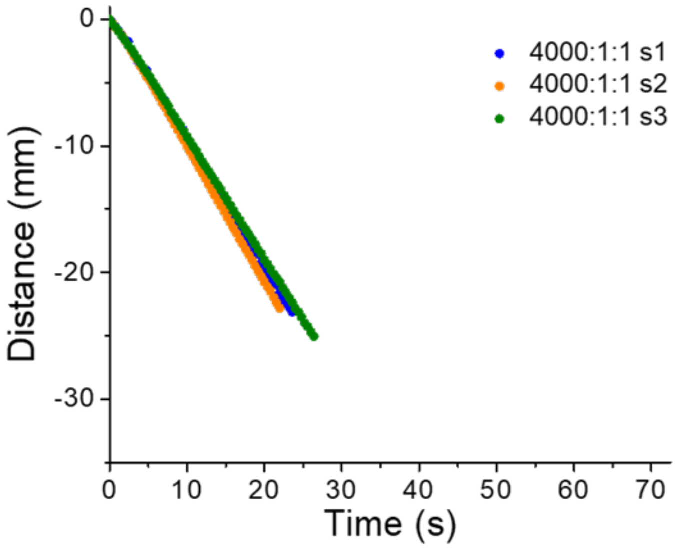

Figure S26: Front tracking of 4000:1:1 25 mol% NBE8 in DCPD-H_2:G2:TBP in triplicate (v_f = 1.01 ± 0.04 mm/s).

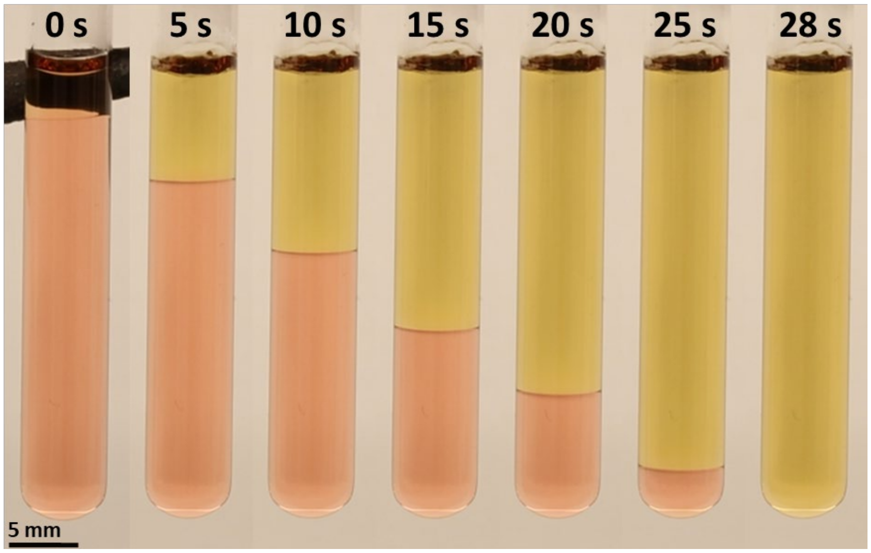

Figure S27: Representative timelapse of 2000:1:1 25 mol% NBE8 in DCPD-H_2:G2:TBP. The scale bar is 5 mm.

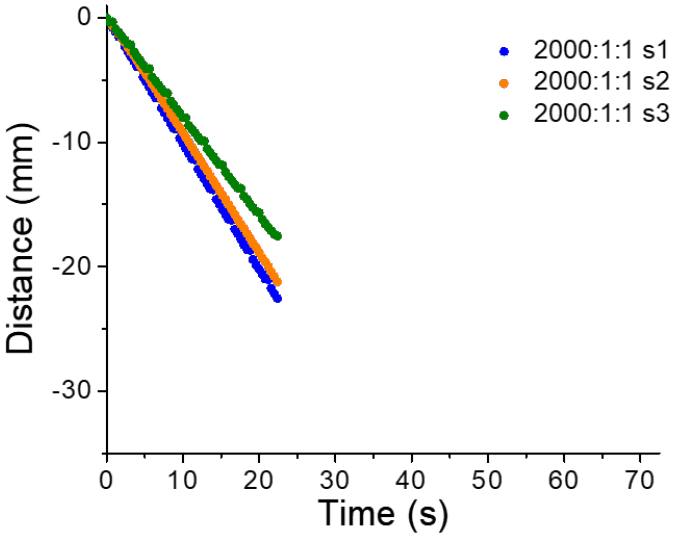

Figure S28: Front tracking of 2000:1:1 25 mol% NBE8 in DCPD-H_2:G2:TBP in triplicate (v_f = 0.92 ± 0.09 mm/s).

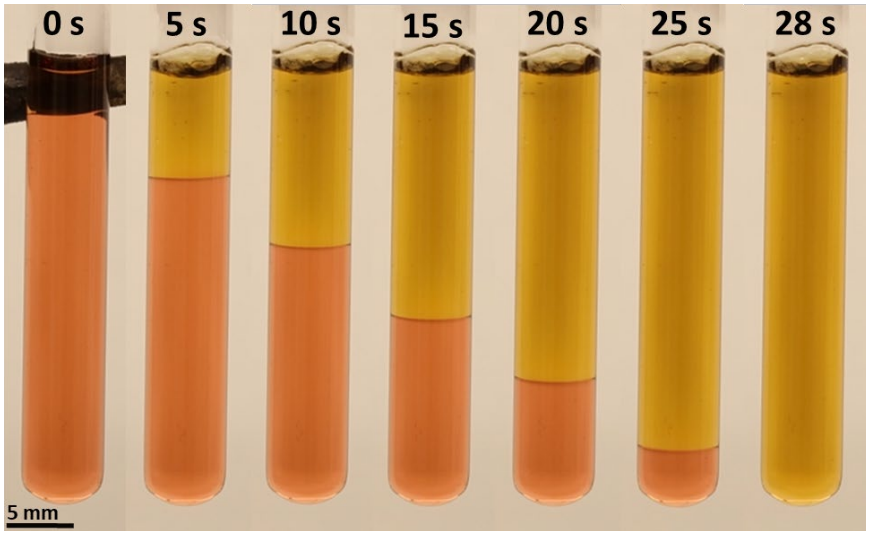

Figure S29: Representative timelapse of 1000:1:1 25 mol% NBE8 in DCPD-H_2:G2:TBP. The scale bar is 5 mm.

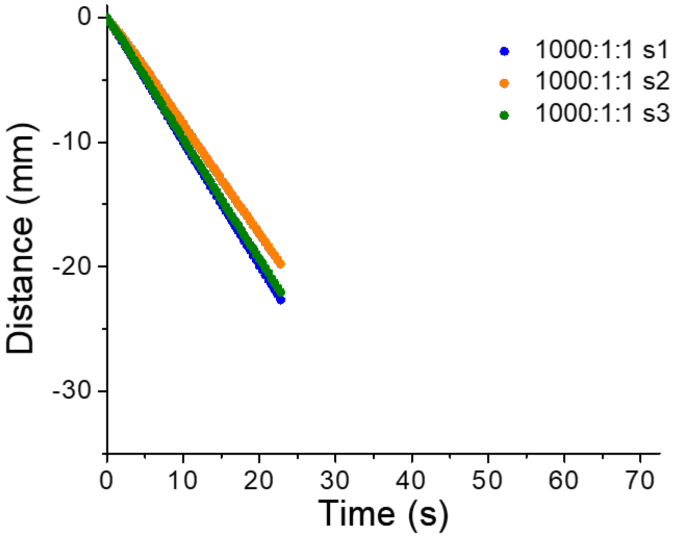

Figure S30: Front tracking of 1000:1:1 25 mol% NBE8 in DCPD-H_2:G2:TBP in triplicate (v_f = 0.95 ± 0.05 mm/s).

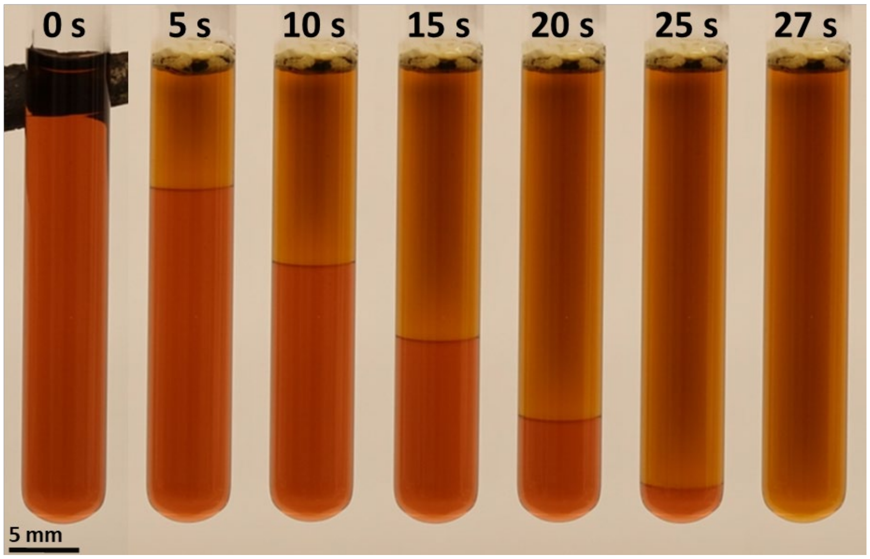

Figure S31: Representative timelapse of 500:1:1 25 mol% NBE8 in DCPD-H_2:G2:TBP. The scale bar is 5 mm.

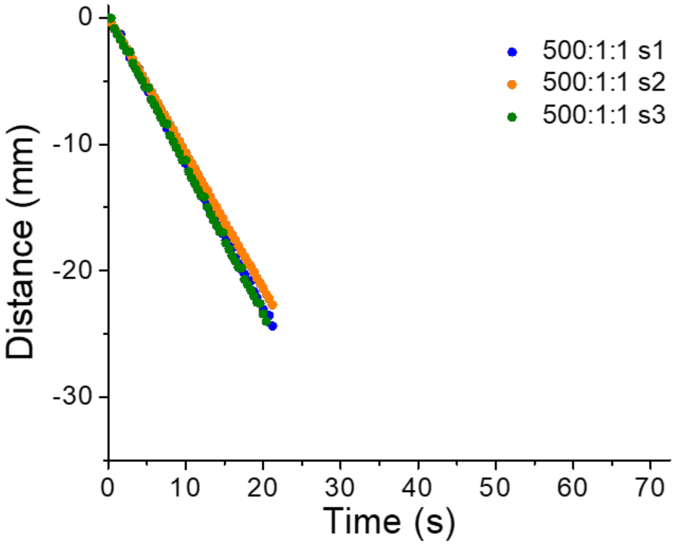

Figure S32: Front tracking of 500:1:1 25 mol% NBE8 in DCPD-H_2:G2:TBP in triplicate (v_f = 1.14 ± 0.04 mm/s).

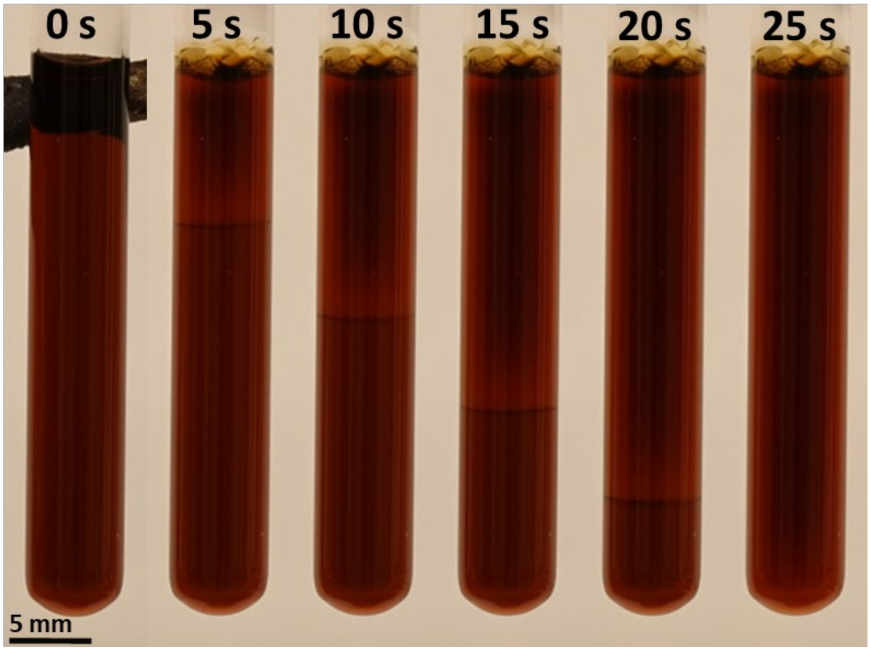

Figure S33: Representative timelapse of 200:1:1 25 mol% NBE8 in DCPD-H_2:G2:TBP. The scale bar is 5 mm.

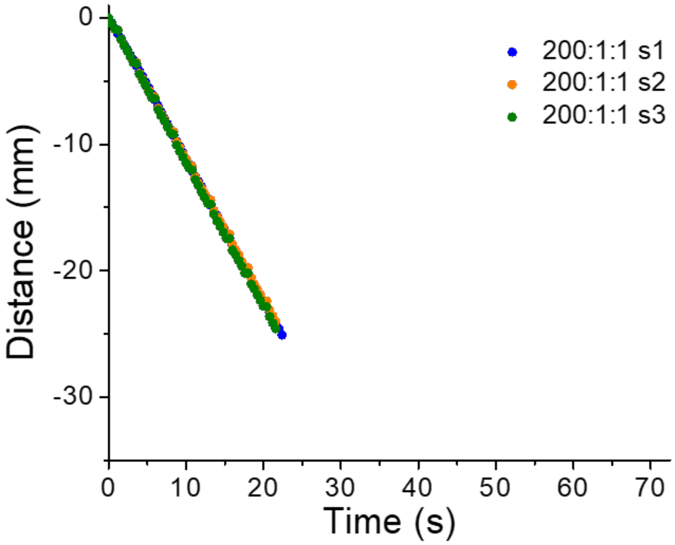

Figure S34: Front tracking of 200:1:1 25 mol% NBE8 in DCPD-H_2:G2:TBP in triplicate (v_f = 1.13 ± 0.01 mm/s).

25 mol% NBE12

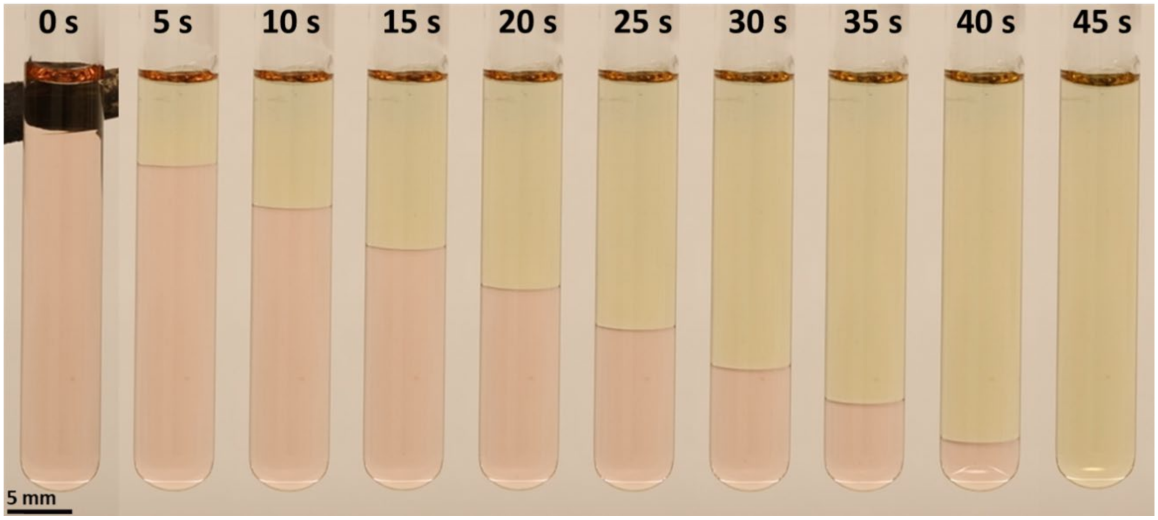

Figure S35: Representative timelapse of 4000:1:1 25 mol% NBE12 in DCPD-H_2:G2:TBP. The scale bar is 5 mm.

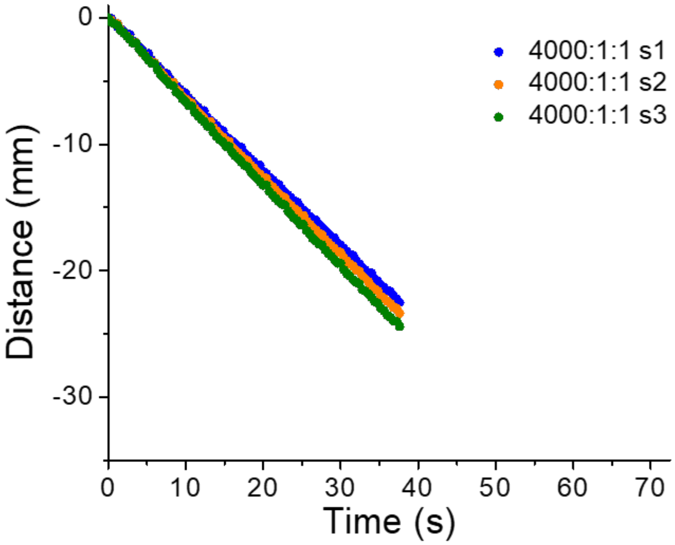

Figure S36: Front tracking of 4000:1:1 25 mol% NBE12 in DCPD-H_2:G2:TBP in triplicate (v_f = 0.62 ± 0.02 mm/s).

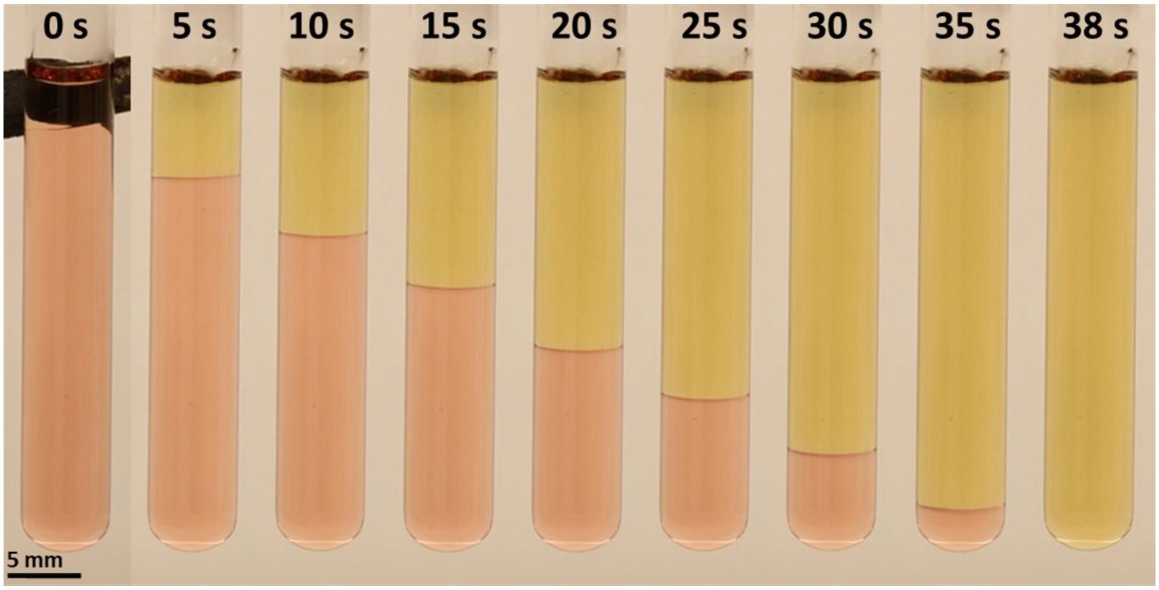

Figure S37: Representative timelapse of 2000:1:1 25 mol% NBE12 in DCPD-H_2:G2:TBP. The scale bar is 5 mm.

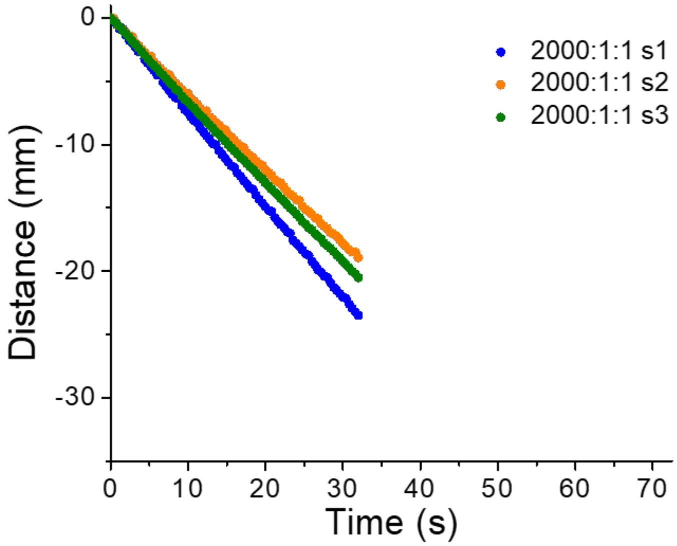

Figure S38: Front tracking of 2000:1:1 25 mol% NBE12 in DCPD-H_2:G2:TBP in triplicate (v_f = 0.66 ± 0.06 mm/s).

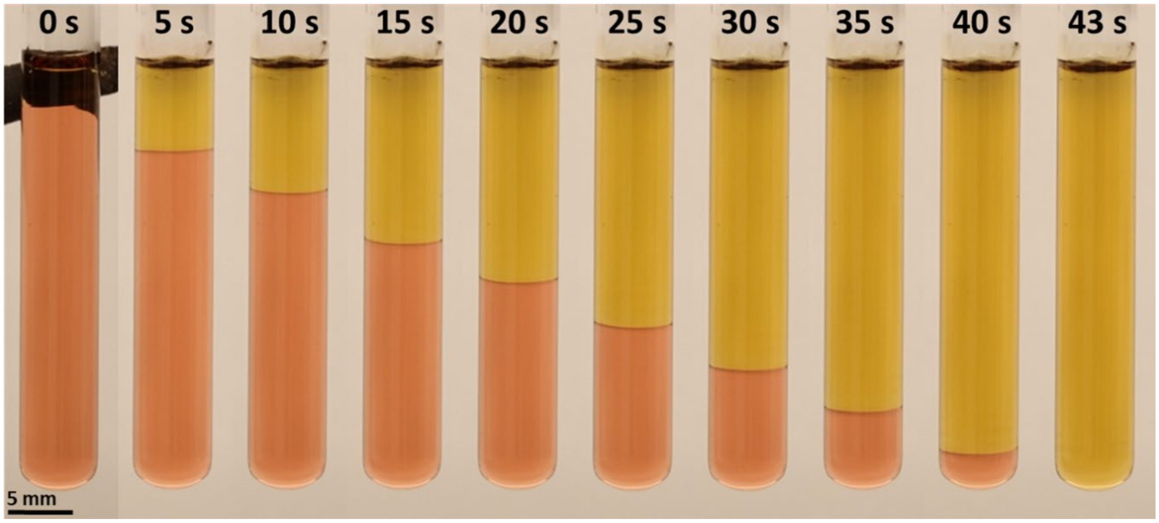

Figure S39: Representative timelapse of 1000:1:1 25 mol% NBE12 in DCPD-H_2:G2:TBP. The scale bar is 5 mm.

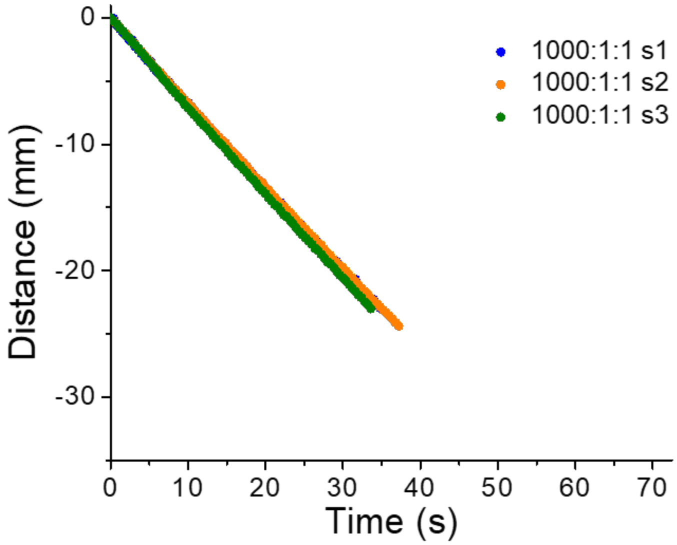

Figure S40: Front tracking of 1000:1:1 25 mol% NBE12 in DCPD-H_2:G2:TBP in triplicate (v_f = 0.67 ± 0.01 mm/s).

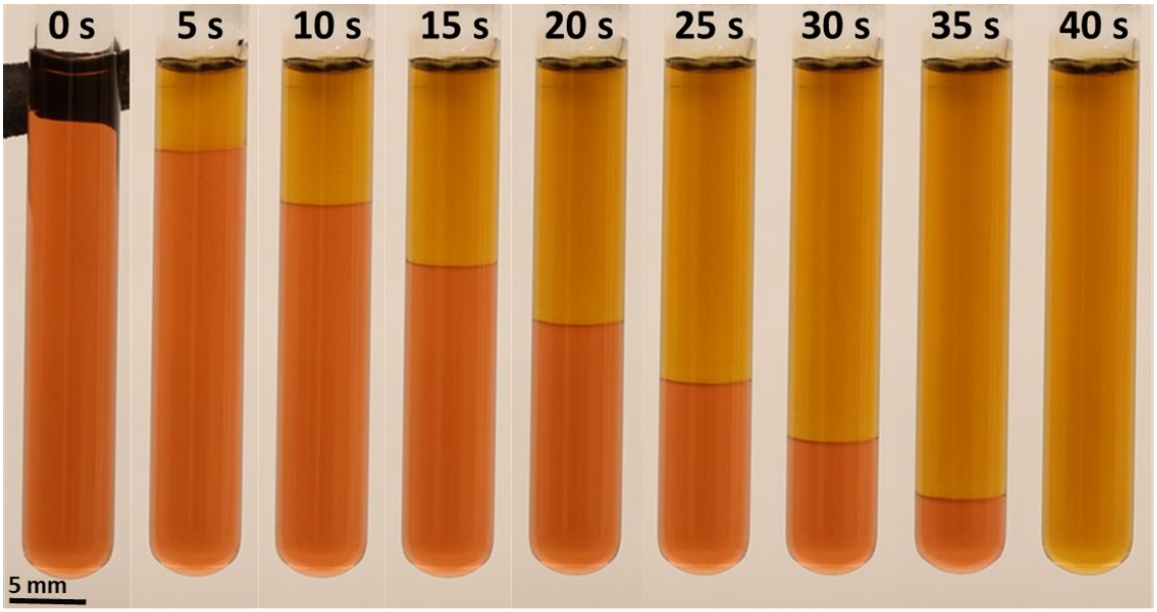

Figure S41: Representative timelapse of 500:1:1 25 mol% NBE12 in DCPD-H_2:G2:TBP. The scale bar is 5 mm.

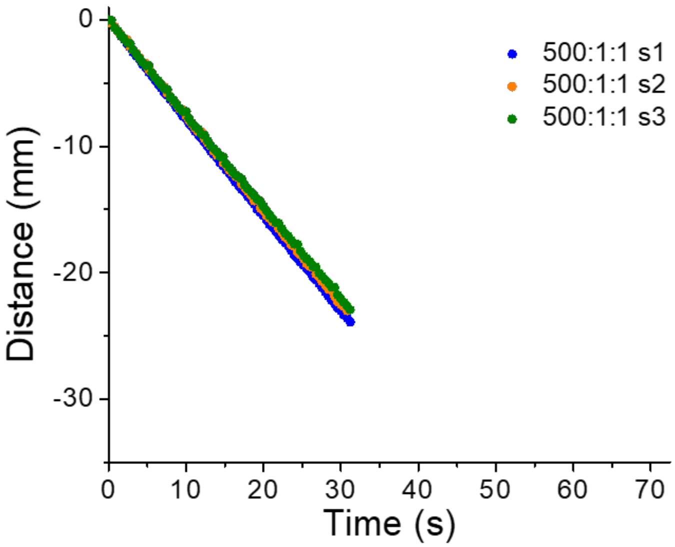

Figure S42: Front tracking of 500:1:1 25 mol% NBE12 in DCPD-H_2:G2:TBP in triplicate (v_f = 0.75 ± 0.02 mm/s).

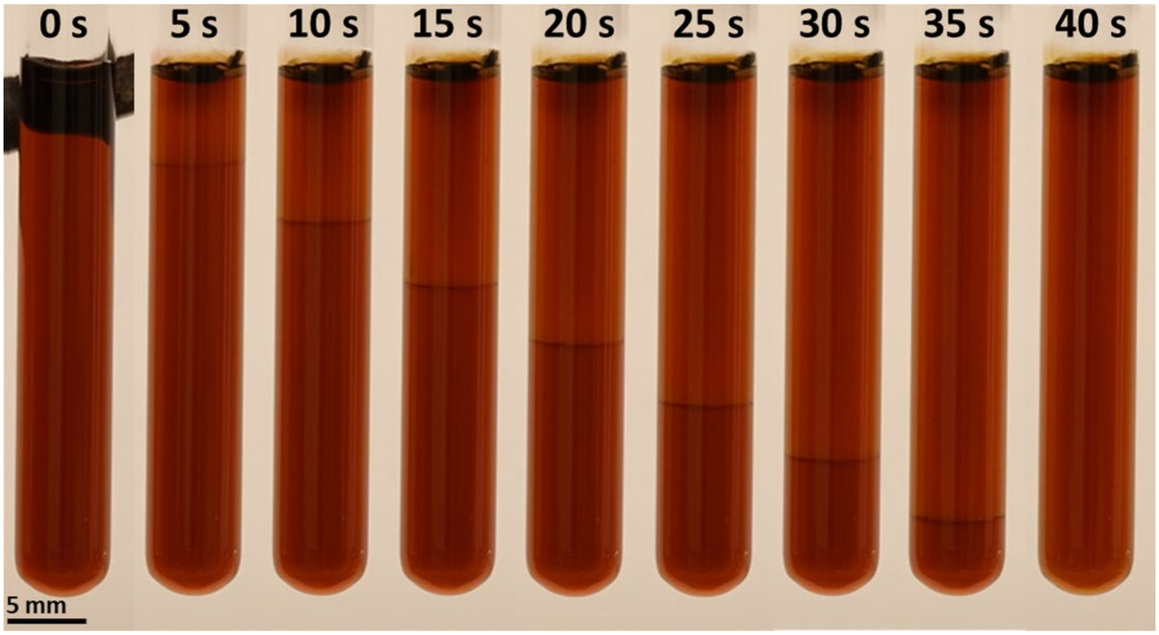

Figure S43: Representative timelapse of 200:1:1 25 mol% NBE12 in DCPD-H_2:G2:TBP. The scale bar is 5 mm.

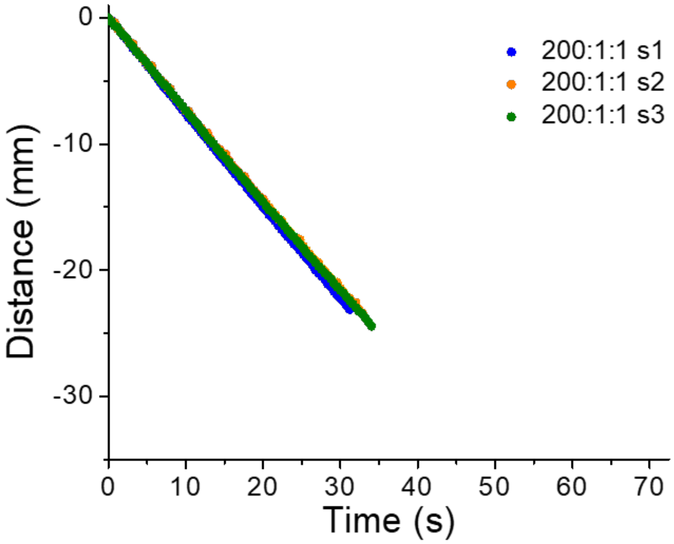

Figure S44: Front tracking of 200:1:1 25 mol% NBE12 in DCPD-H_2:G2:TBP in triplicate (v_f = 0.73 ± 0.01 mm/s).

25 mol% NBE16

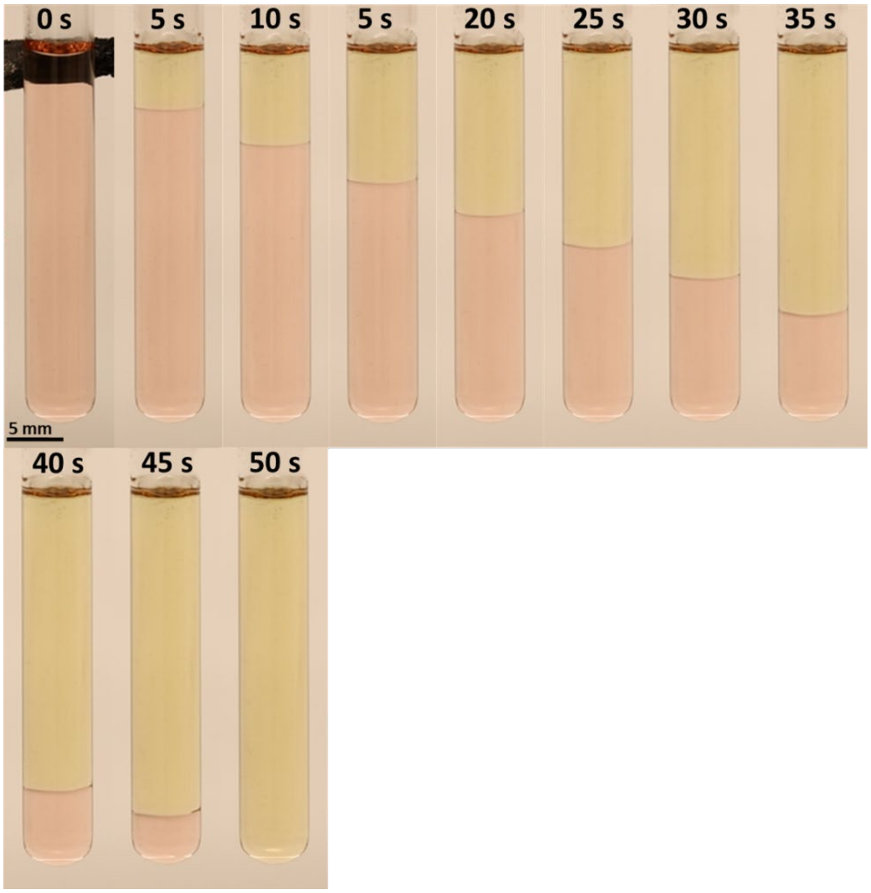

Figure S45: Representative timelapse of 4000:1:1 25 mol% NBE16 in DCPD-H_2:G2:TBP. The scale bar is 5 mm.

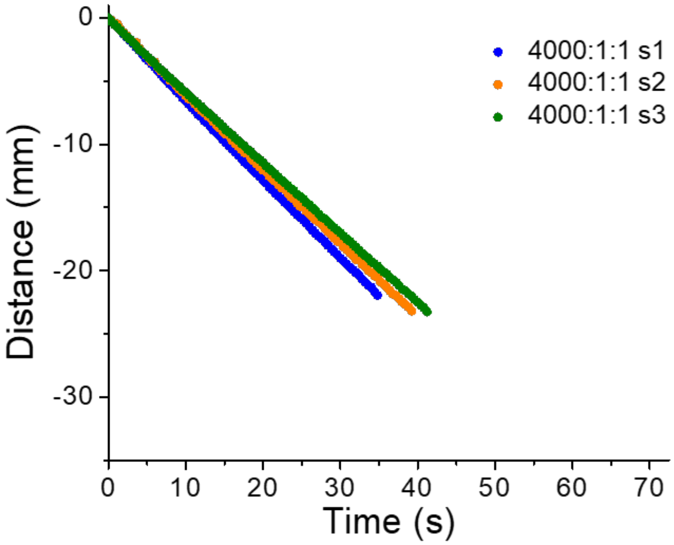

Figure S46: Front tracking of 4000:1:1 25 mol% NBE16 in DCPD-H_2:G2:TBP in triplicate (v_f = 0.59 ± 0.03 mm/s).

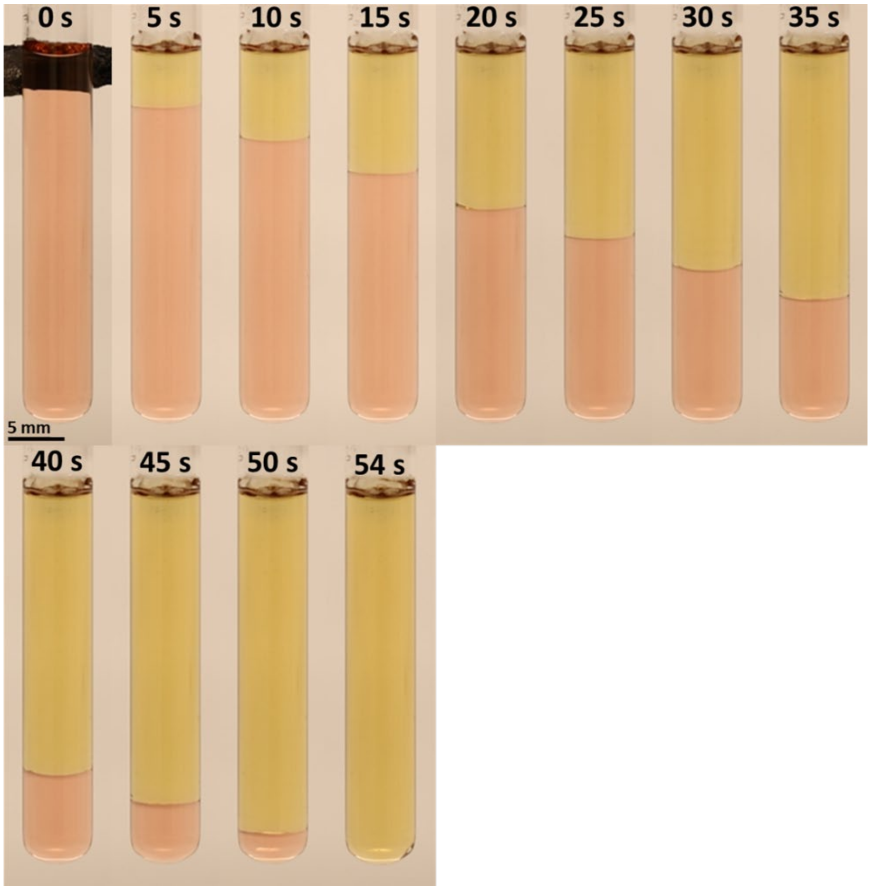

Figure S47: Representative timelapse of 2000:1:1 25 mol% NBE16 in DCPD-H_2:G2:TBP. The scale bar is 5 mm.

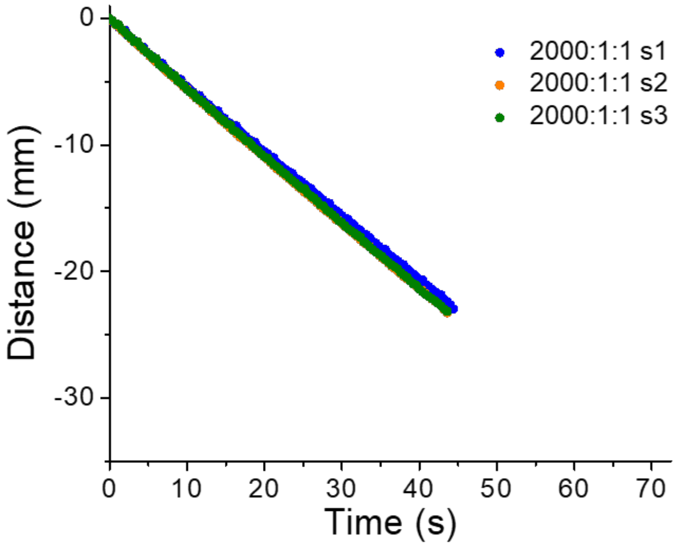

Figure S48: Front tracking of 2000:1:1 25 mol% NBE16 in DCPD-H_2:G2:TBP in triplicate (v_f = 0.52 ± 0.01 mm/s).

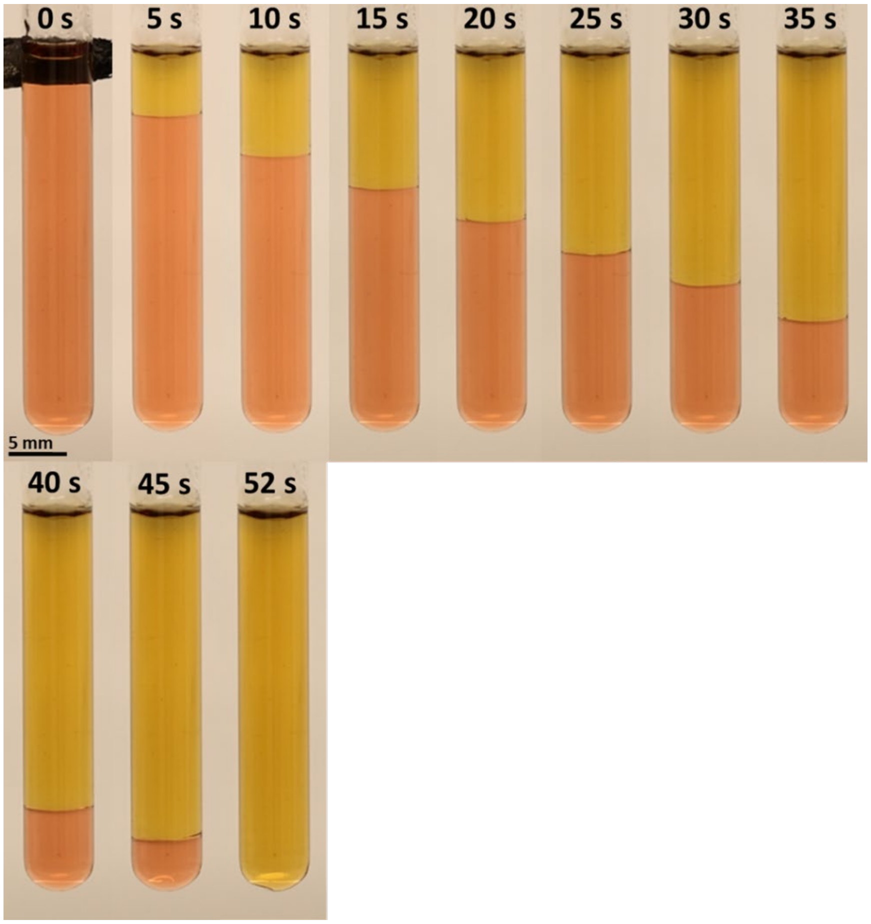

Figure S49: Representative timelapse of 1000:1:1 25 mol% NBE16 in DCPD-H_2:G2:TBP. The scale bar is 5 mm.

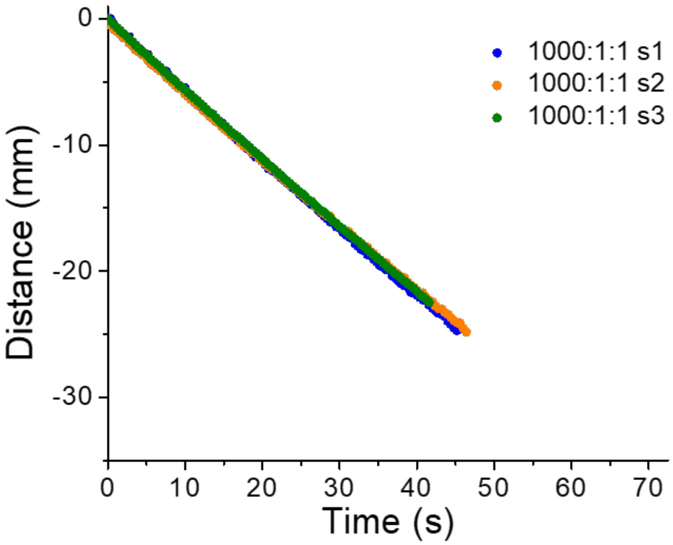

Figure S50: Front tracking of 1000:1:1 25 mol% NBE16 in DCPD-H_2:G2:TBP in triplicate (v_f = 0.53 ± 0.01 mm/s).

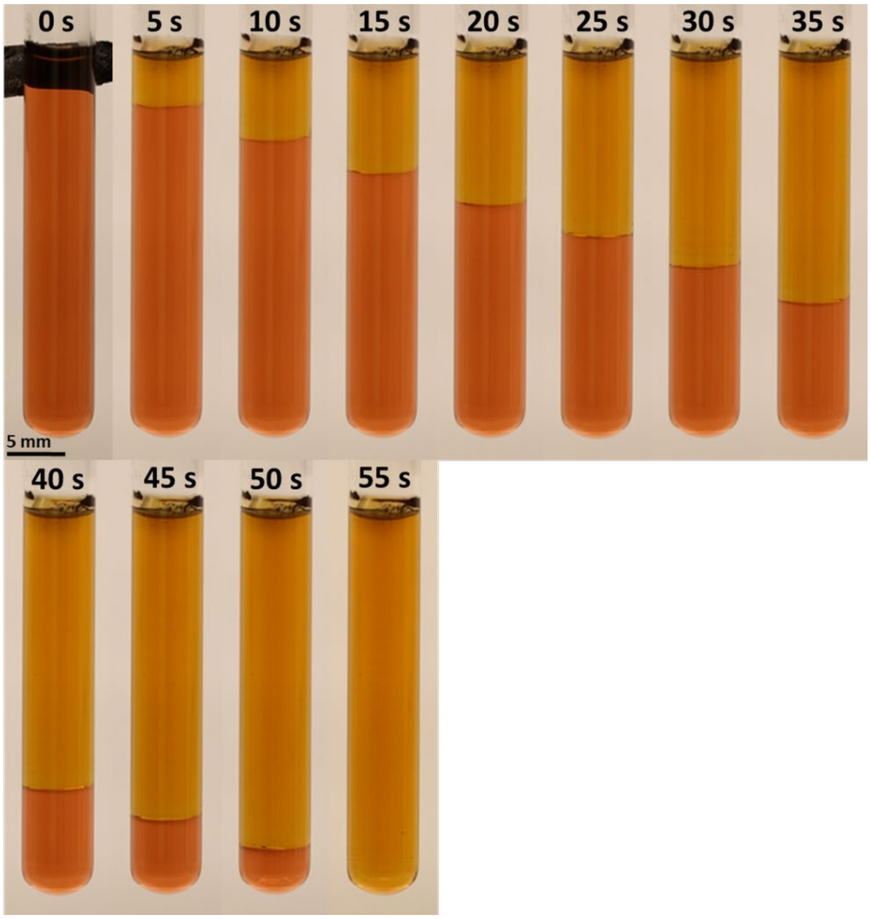

Figure S51: Representative timelapse of 500:1:1 25 mol% NBE16 in DCPD-H_2:G2:TBP. The scale bar is 5 mm.

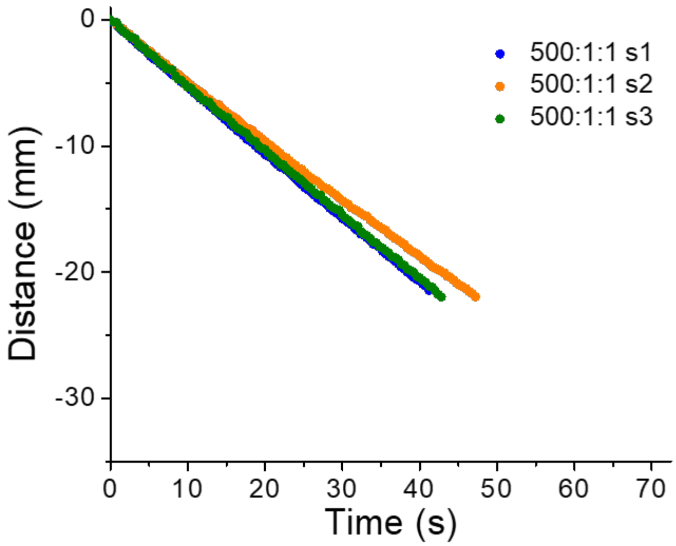

Figure S52: Front tracking of 500:1:1 25 mol% NBE16 in DCPD-H_2:G2:TBP in triplicate (v_f = 0.50 ± 0.03 mm/s).

Table S1: Front velocity (v_f) for copolymerization of NBE monomers in DCPD-H_2 resins at varied loadings.

Monomer (equiv)	Initiator (equiv)	Inhibitor (equiv)	Molar incorporation of NBE monomer (%)	Front velocity (mm/s)	error
NBE8					
1000	1	1	50	0.31	0.06
500	1	1	50	0.39	0.04
200	1	1	50	0.39	0.03
NBE8					
4000	1	1	25	1.01	0.04
2000	1	1	25	0.92	0.09
1000	1	1	25	0.95	0.05
500	1	1	25	1.14	0.04
200	1	1	25	1.13	0.01
NBE12					
4000	1	1	25	0.62	0.02
2000	1	1	25	0.66	0.06
1000	1	1	25	0.67	0.01
500	1	1	25	0.75	0.02
200	1	1	25	0.73	0.01
NBE16					
4000	1	1	25	0.59	0.03
2000	1	1	25	0.52	0.01
1000	1	1	25	0.53	0.01
500	1	1	25	0.50	0.03

DSC (Cure kinetics):

50 mol% NBE8

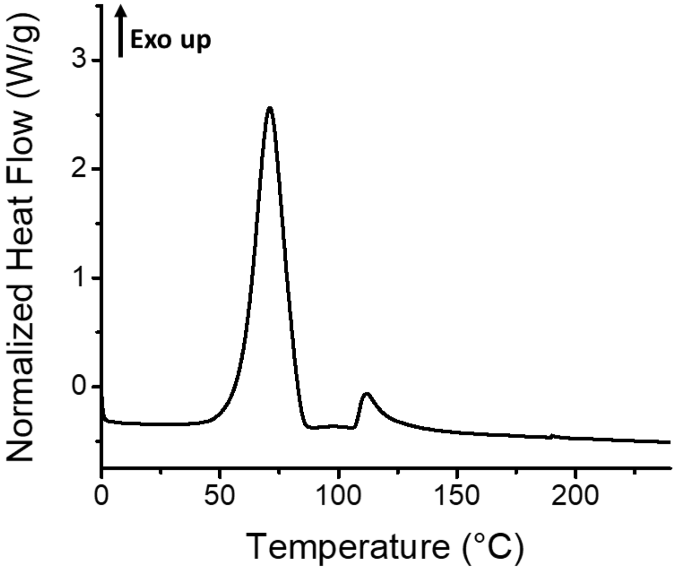

Figure S53: Representative DSC cure kinetic profile of 1000:1:1 50 mol% NBE8 in DCPD-H_2:G2:TBP (H_r = 300 ± 7 J/g, Peak Temp = 72.8 ± 1.6 °C).

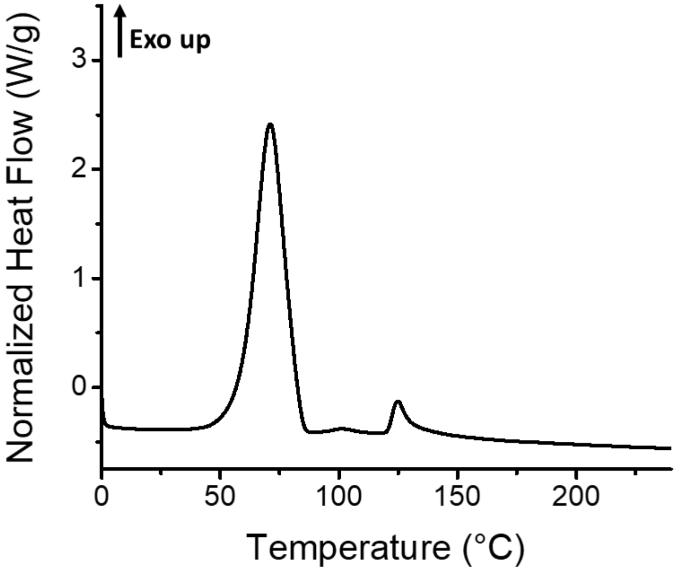

Figure S54: Representative DSC cure kinetic profile of 500:1:1 50 mol% NBE8 in DCPD-H_2:G2:TBP (H_r = 295 ± 7 J/g, Peak Temp = 71.8 ± 0.7 °C).

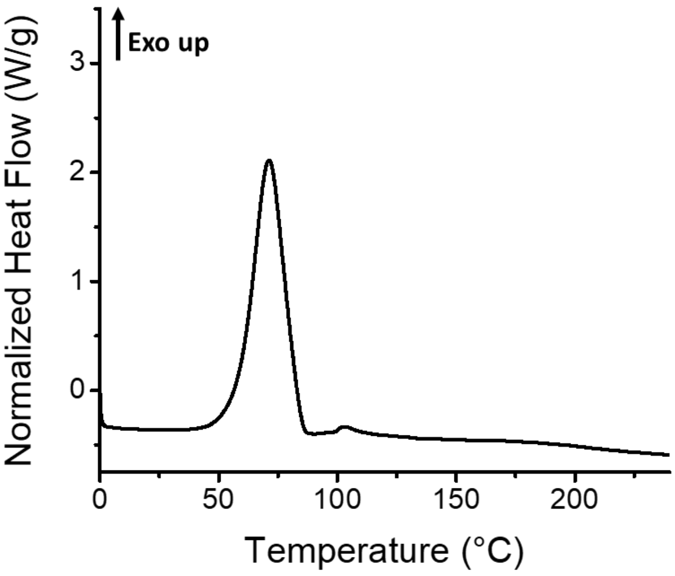

Figure S55: Representative DSC cure kinetic profile of 200:1:1 50 mol% NBE8 in DCPD-H_2:G2:TBP (H_r = 259 ± 6 J/g, Peak Temp = 71.9 ± 0.5 °C).

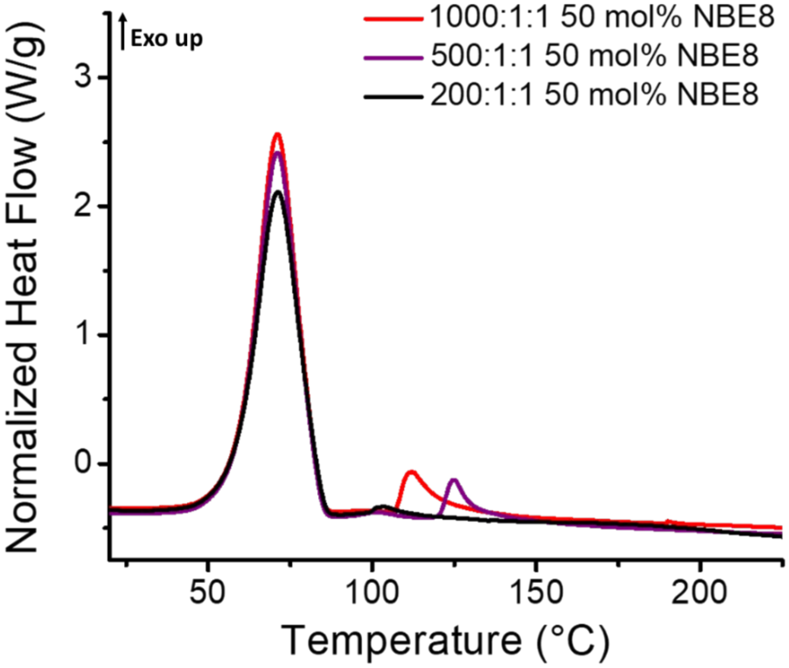

Figure S56: Stacked representative DSC cure kinetic profile for x:1:1 50 mol% NBE8 in DCPD-H_2:G2:TBP.

25 mol% NBE8

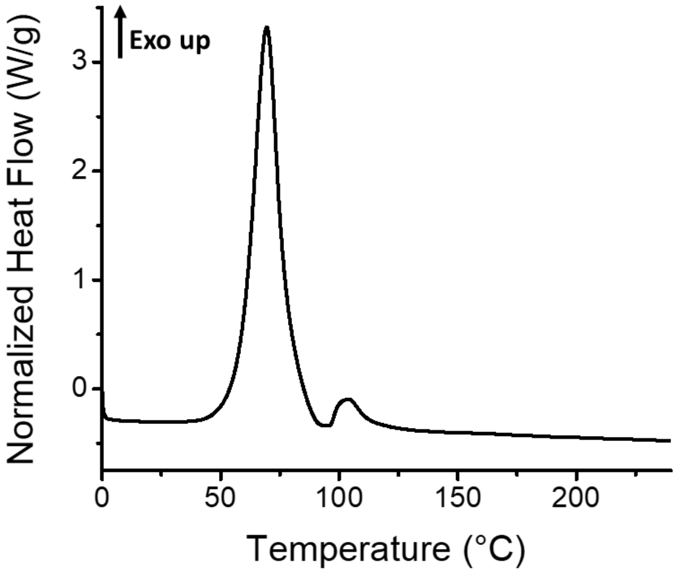

Figure S57: Representative DSC cure kinetic profile of 4000:1:1 25 mol% NBE8 in DCPD-H_2:G2:TBP (H_r = 362 ± 4 J/g, Peak Temp = 69.7 ± 0.6 °C).

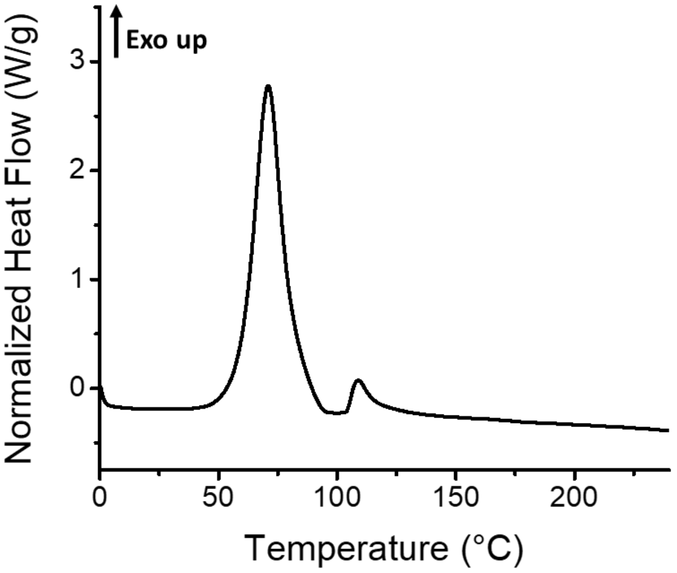

Figure S58: Representative DSC cure kinetic profile of 2000:1:1 25 mol% NBE8 in DCPD-H_2:G2:TBP (H_r = 346 ± 13 J/g, Peak Temp = 72.0 ± 0.9 °C).

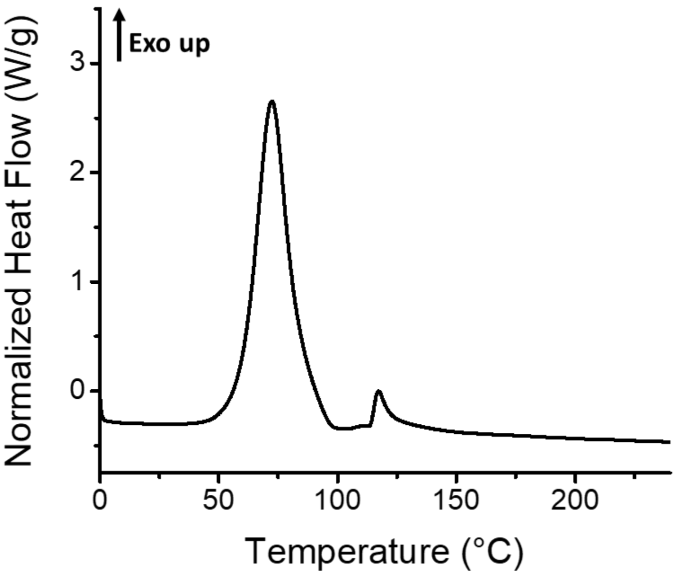

Figure S59: Representative DSC cure kinetic profile of 1000:1:1 25 mol% NBE8 in DCPD-H_2:G2:TBP (H_r = 348 ± 6 J/g, Peak Temp = 72.1 ± 0.3 °C).

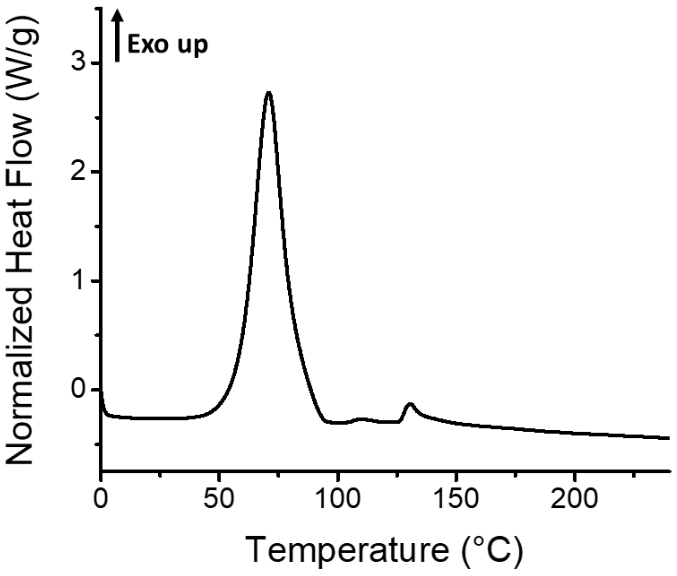

Figure S60: Representative DSC cure kinetic profile of 500:1:1 25 mol% NBE8 in DCPD-H_2:G2:TBP (H_r = 330 ± 3 J/g, Peak Temp = 71.1 ± 0.3 °C).

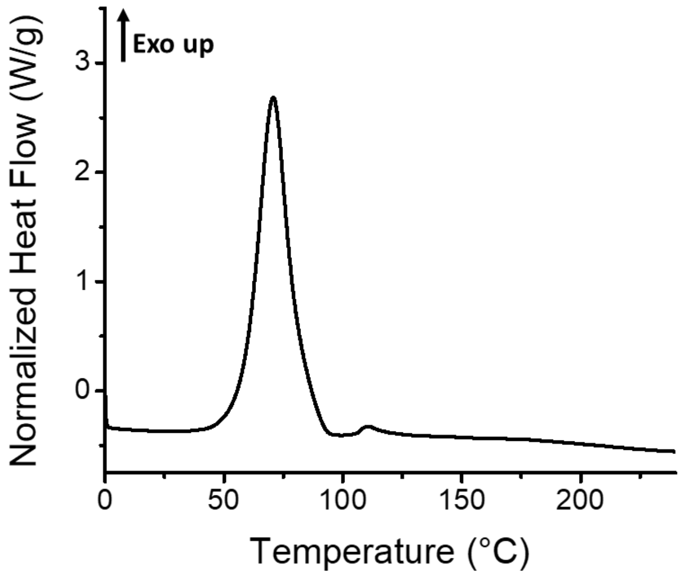

Figure S61: Representative DSC cure kinetic profile of 200:1:1 25 mol% NBE8 in DCPD-H_2:G2:TBP (H_r = 316 ± 6 J/g, Peak Temp = 70.9 ± 0.1 °C).

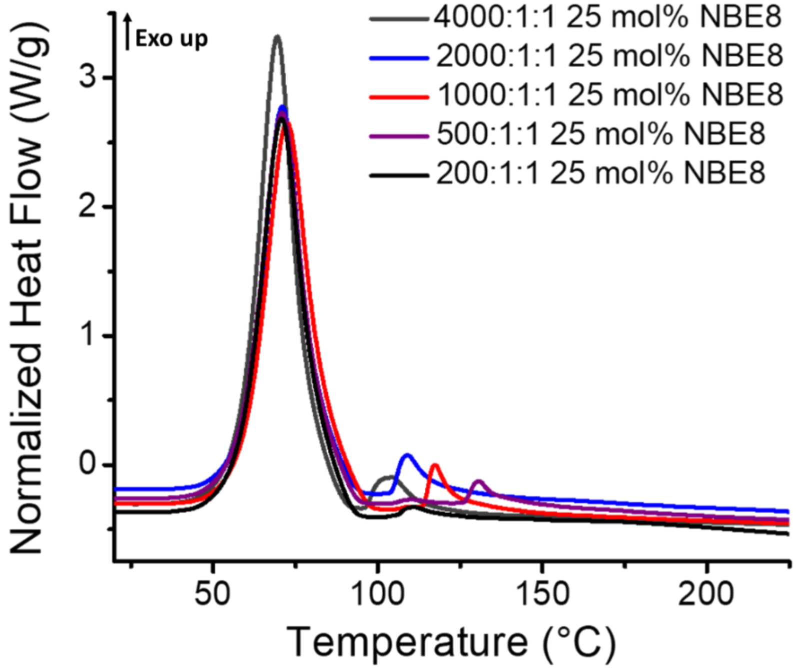

Figure S62: Stacked representative DSC cure kinetic profile for x:1:1 25 mol% NBE8 in DCPD-H_2:G2:TBP.

25 mol% NBE12

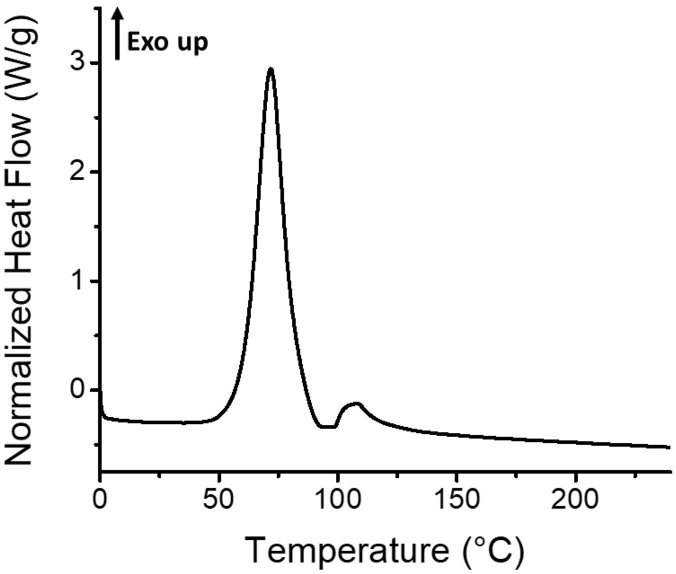

Figure S63: Representative DSC cure kinetic profile of 4000:1:1 25 mol% NBE12 in DCPD-H_2:G2:TBP (H_r = 339 ± 1 J/g, Peak Temp = 72.3 ± 0.4 °C).

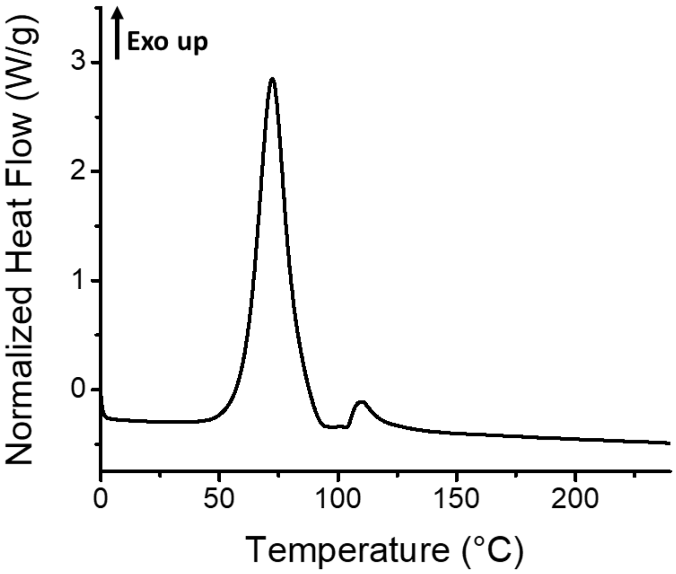

Figure S64: Representative DSC cure kinetic profile of 2000:1:1 25 mol% NBE12 in DCPD-H_2:G2:TBP (H_r = 324 ± 9 J/g, Peak Temp = 72.3 ± 0.9 °C).

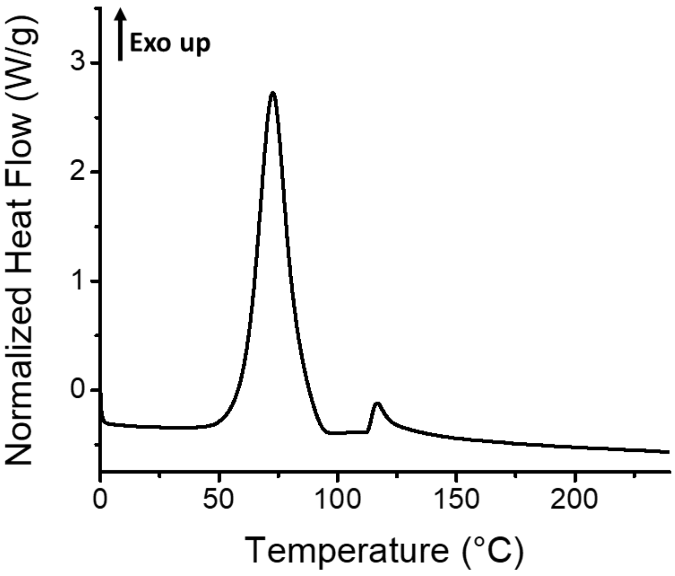

Figure S65: Representative DSC cure kinetic profile of 1000:1:1 25 mol% NBE12 in DCPD-H_2:G2:TBP (H_r = 332 ± 7 J/g, Peak Temp = 72.5 ± 0.3 °C).

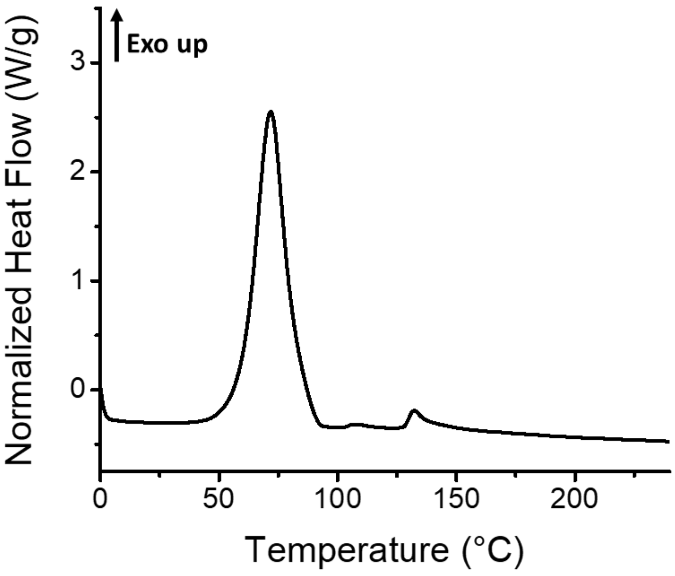

Figure S66: Representative DSC cure kinetic profile of 500:1:1 25 mol% NBE12 in DCPD-H_2:G2:TBP (H_r = 321 ± 8 J/g, Peak Temp = 71.7 ± 0.1 °C).

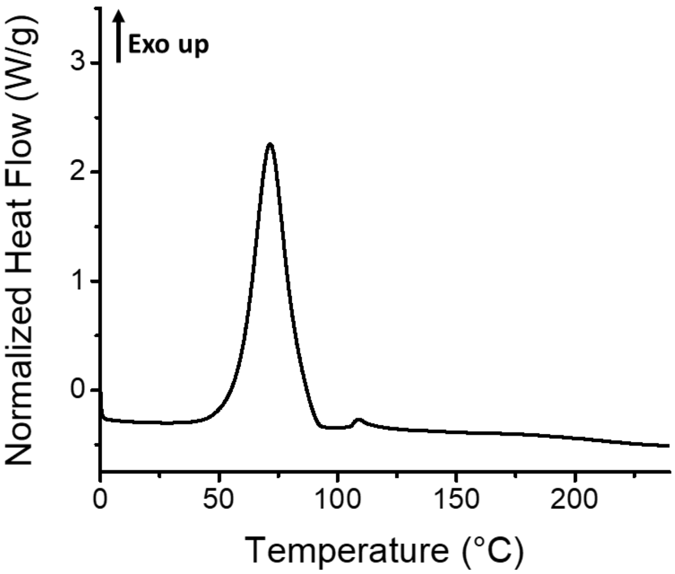

Figure S67: Representative DSC cure kinetic profile of 200:1:1 25 mol% NBE12 in DCPD-H_2:G2:TBP (H_r = 296 ± 6 J/g, Peak Temp = 71.6 ± 0.1 °C).

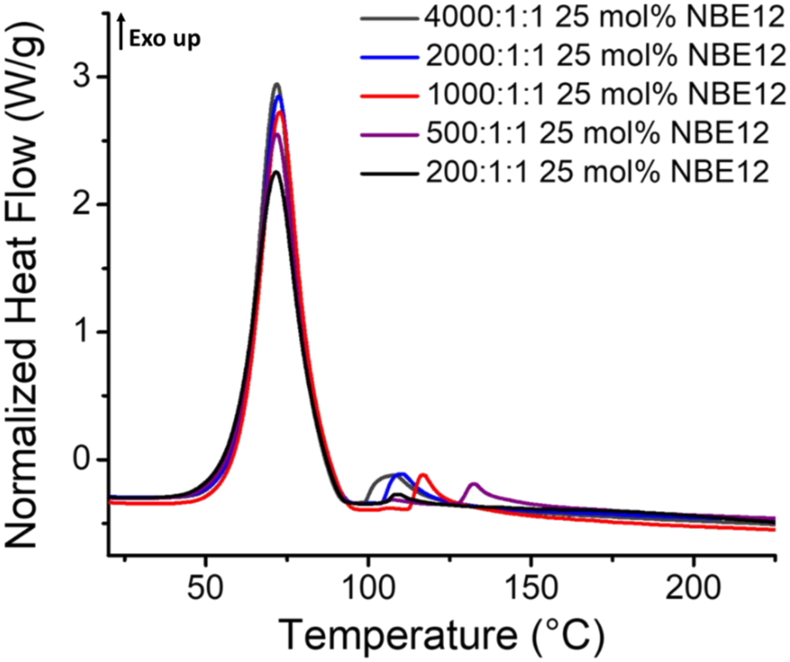

Figure S68: Stacked representative DSC cure kinetic profile for x:1:1 25 mol% NBE12 in DCPD-H_2:G2:TBP.

25 mol% NBE16

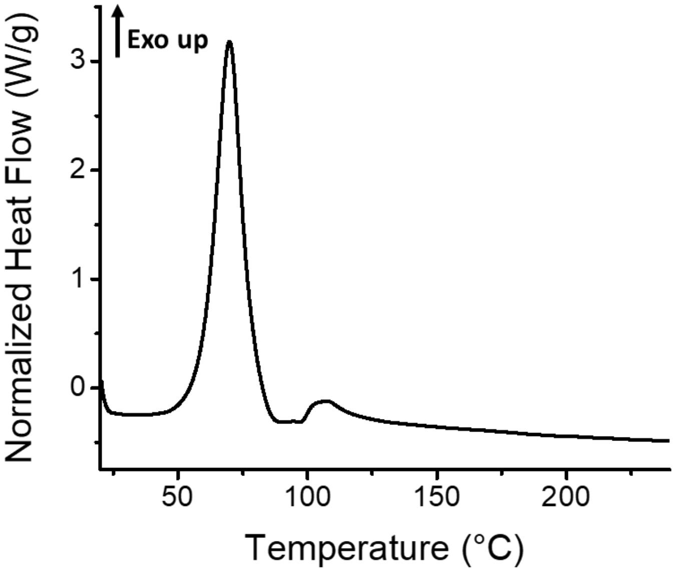

Figure S69: Representative DSC cure kinetic profile of 4000:1:1 25 mol% NBE16 in DCPD-H_2:G2:TBP (H_r = 301 ± 7 J/g, Peak Temp = 70.4 ± 0.5 °C).

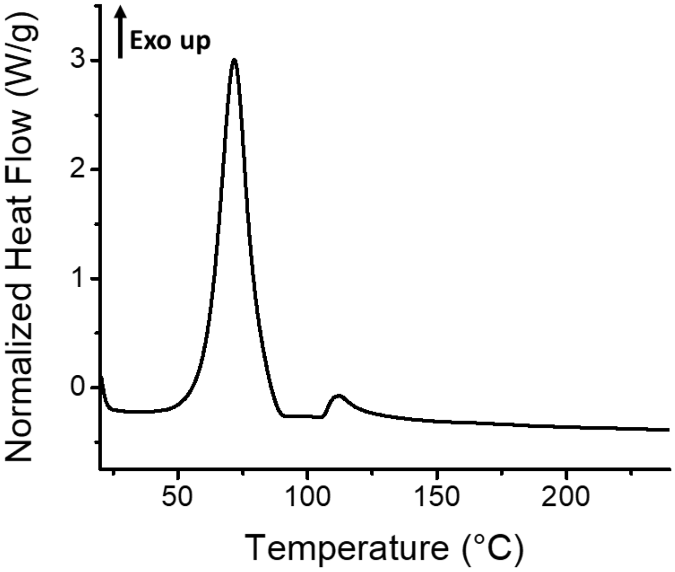

Figure S70: Representative DSC cure kinetic profile of 2000:1:1 25 mol% NBE16 in DCPD-H_2:G2:TBP (H_r = 299 ± 3 J/g, Peak Temp = 71.3 ± 0.3 °C).

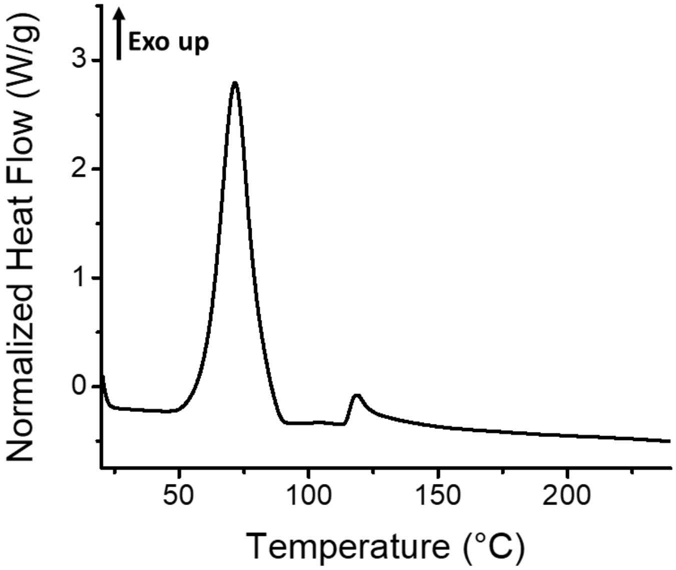

Figure S71: Representative DSC cure kinetic profile of 1000:1:1 25 mol% NBE16 in DCPD-H_2:G2:TBP (H_r = 299 ± 10 J/g, Peak Temp = 71.6 ± 0.2 °C).

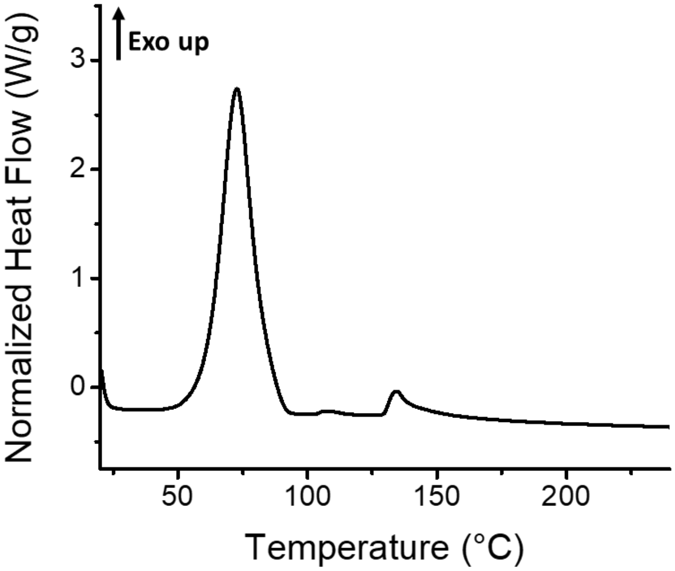

Figure S72: Representative DSC cure kinetic profile of 500:1:1 25 mol% NBE16 in DCPD-H_2:G2:TBP (H_r = 308 ± 2 J/g, Peak Temp = 72.2 ± 0.5 °C).

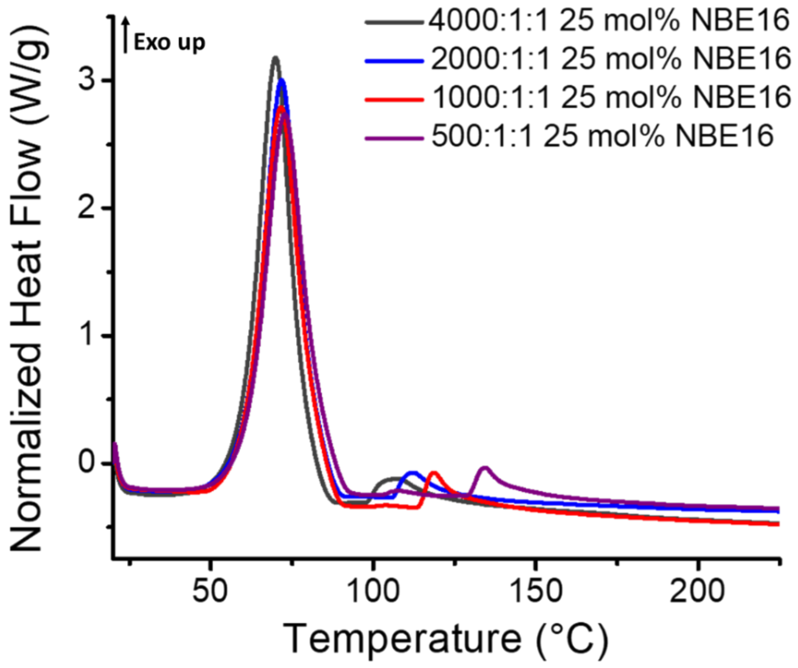

Figure S73: Stacked representative DSC cure kinetic profile for x:1:1 25 mol% NBE16 in DCPD-H_2:G2:TBP.

Table S2: Heat of reaction (H_r) and peak temperature (T_{peak}) for DCPD-H_2 resins at varied loadings.

Monomer (equiv)	Initiator (equiv)	Inhibitor (equiv)	Molar incorporation of NBE monomer (%)	H_r (J/g)	error	T_{peak} (°C)	error
NBE8							
1000	1	1	50	300	7	72.8	1.6
500	1	1	50	295	7	71.8	0.7
200	1	1	50	259	6	71.9	0.5
NBE8							
4000	1	1	25	362	4	69.7	0.6
2000	1	1	25	346	13	72.0	0.9
1000	1	1	25	348	6	72.1	0.3
500	1	1	25	330	3	71.1	0.3
200	1	1	25	316	6	70.9	0.1
NBE12							
4000	1	1	25	339	1	72.3	0.4
2000	1	1	25	324	9	72.3	0.9
1000	1	1	25	332	7	72.5	0.3
500	1	1	25	321	8	71.7	0.1
200	1	1	25	296	6	71.6	0.1
NBE16							
4000	1	1	25	301	7	70.4	0.5
2000	1	1	25	299	3	71.3	0.3
1000	1	1	25	299	10	71.6	0.2
500	1	1	25	308	2	72.2	0.5

Sample Images:

50 mol% NBE8

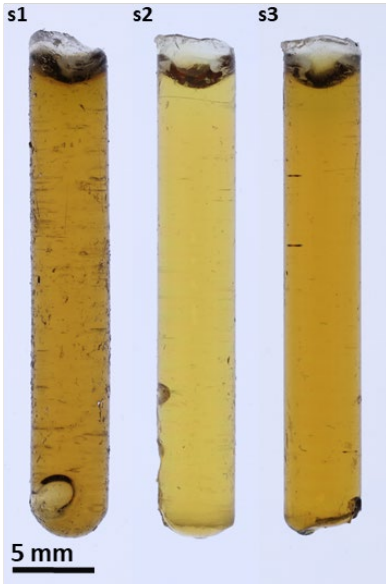

Figure S74: Image of triplicate of 50 mol% pNBE8-*co*-DCPD-H_2 at 1000:1:1 post-FROMP in DCPD-H_2:G2:TBP.

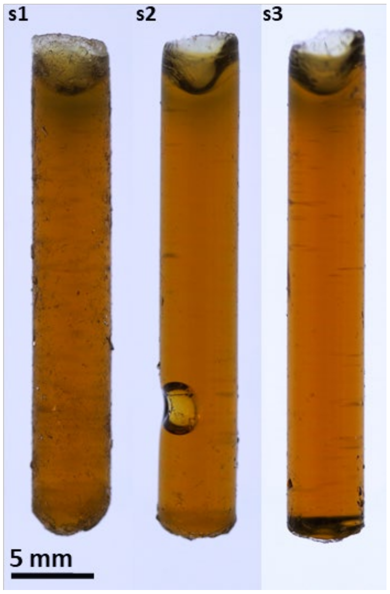

Figure S75: Image of triplicate of 50 mol% pNBE8-*co*-DCPD-H_2 at 500:1:1 post-FROMP in DCPD-H_2:G2:TBP.

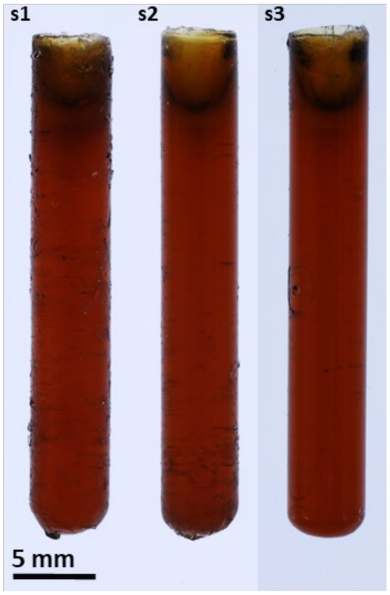

Figure S76: Image of triplicate of 50 mol% pNBE8-*co*-DCPD-H_2 at 200:1:1 post-FROMP in DCPD-H_2:G2:TBP.

<u>25 mol% NBE8</u>

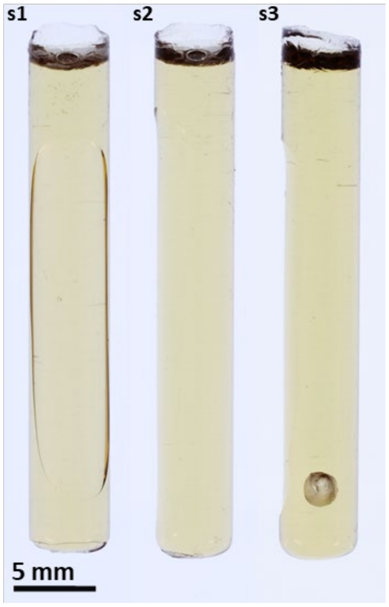

Figure S77: Image of triplicate of 25 mol% pNBE8-*co*-DCPD-H_2 at 4000:1:1 post-FROMP in DCPD-H_2:G2:TBP.

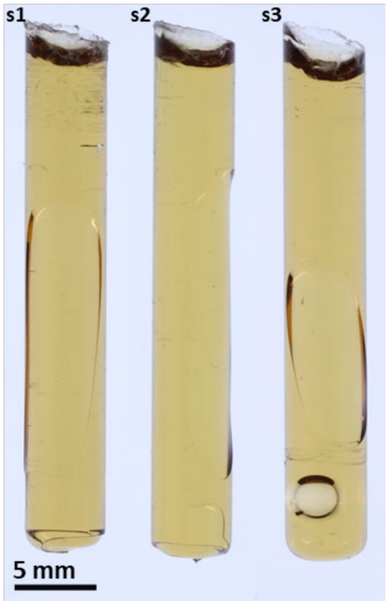

Figure S78: Image of triplicate of 25 mol% pNBE8-*co*-DCPD-H_2 at 2000:1:1 post-FROMP in DCPD-H_2:G2:TBP.

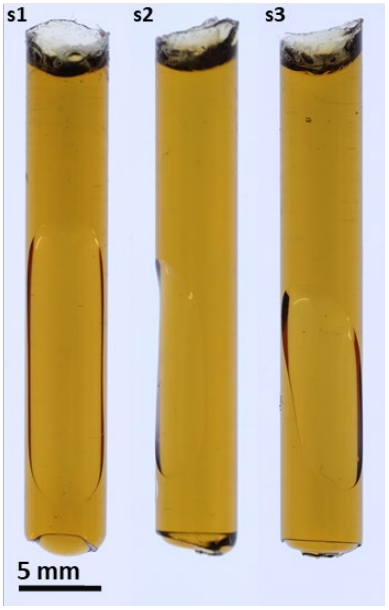

Figure S79: Image of triplicate of 25 mol% pNBE8-*co*-DCPD-H_2 at 1000:1:1 post-FROMP in DCPD-H_2:G2:TBP.

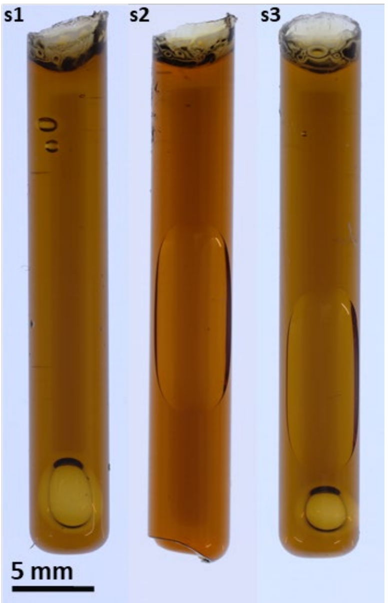

Figure S80: Image of triplicate of 25 mol% pNBE8-*co*-DCPD-H_2 at 500:1:1 post-FROMP in DCPD-H_2:G2:TBP.

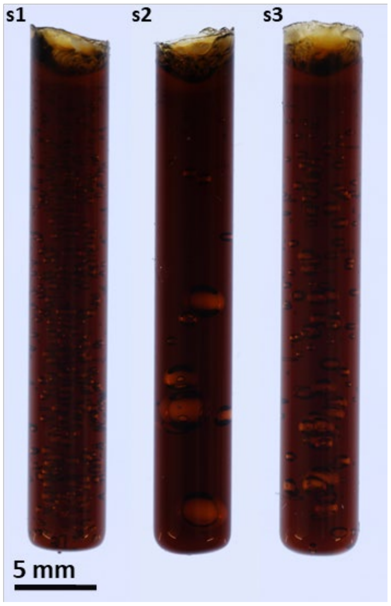

Figure S81: Image of triplicate of 25 mol% pNBE8-*co*-DCPD-H_2 at 200:1:1 post-FROMP in DCPD-H_2:G2:TBP.

25 mol% NBE12

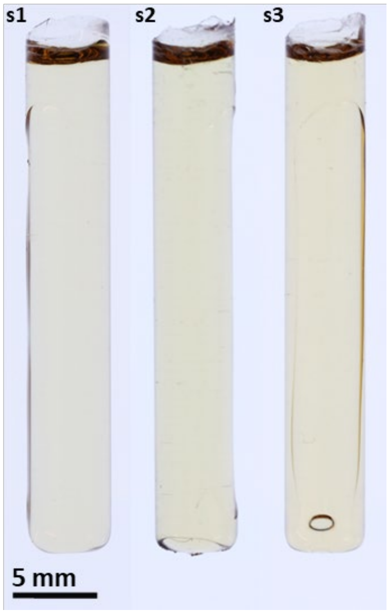

Figure S82: Image of triplicate of 25 mol% pNBE12-*co*-DCPD-H_2 at 4000:1:1 post-FROMP in DCPD-H_2:G2:TBP.

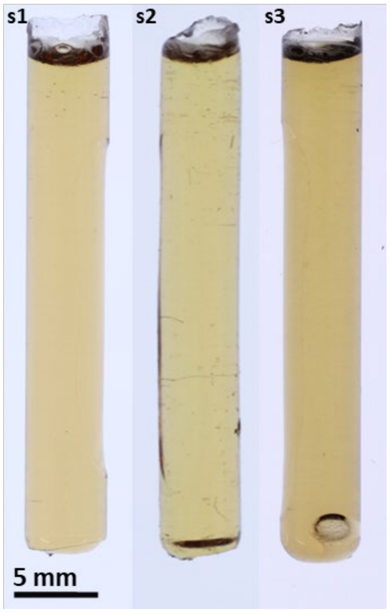

Figure S83: Image of triplicate of 25 mol% pNBE12-*co*-DCPD-H_2 at 2000:1:1 post-FROMP in DCPD-H_2:G2:TBP.

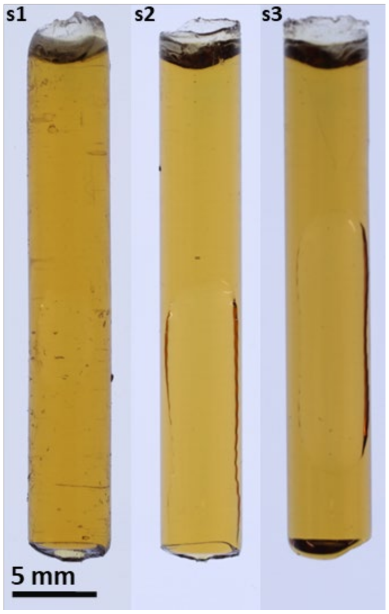

Figure S84: Image of triplicate of 25 mol% pNBE12-*co*-DCPD-H_2 at 1000:1:1 post-FROMP in DCPD-H_2:G2:TBP.

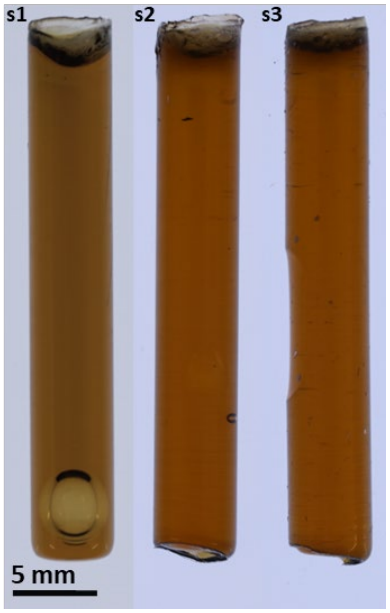

Figure S85: Image of triplicate of 25 mol% pNBE12-*co*-DCPD-H_2 at 500:1:1 post-FROMP in DCPD-H_2:G2:TBP.

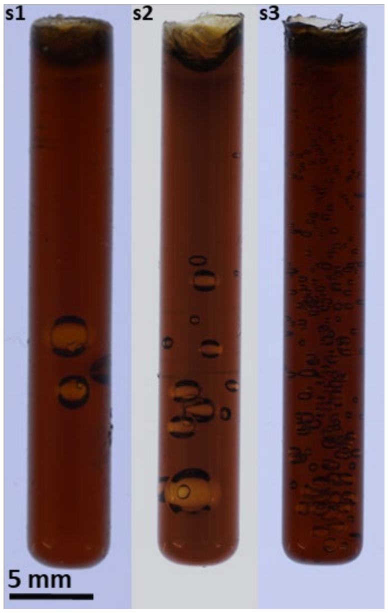

Figure S86: Image of triplicate of 25 mol% pNBE12-*co*-DCPD-H_2 at 200:1:1 post-FROMP in DCPD-H_2:G2:TBP.

<u>25 mol% NBE16</u>

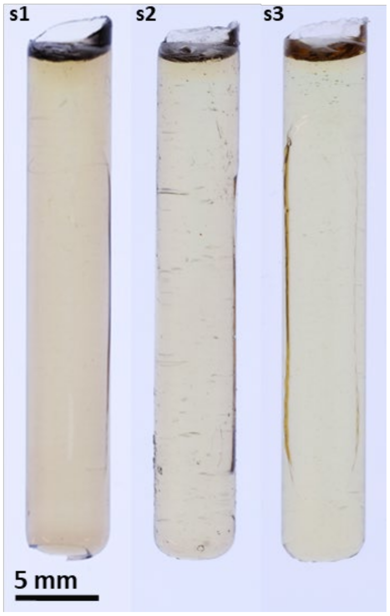

Figure S87: Image of triplicate of 25 mol% pNBE16-*co*-DCPD-H_2 at 4000:1:1 post-FROMP in DCPD-H_2:G2:TBP.

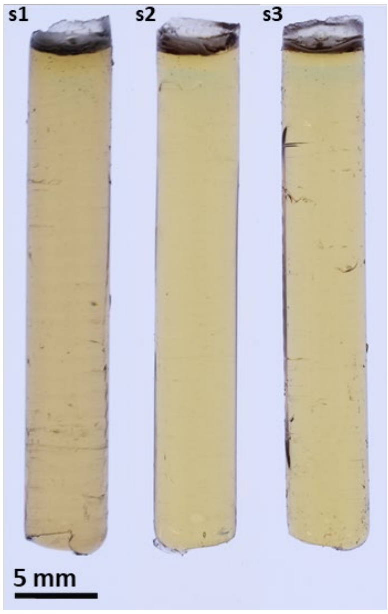

Figure S88: Image of triplicate of 25 mol% pNBE16-*co*-DCPD-H_2 at 2000:1:1 post-FROMP in DCPD-H_2:G2:TBP.

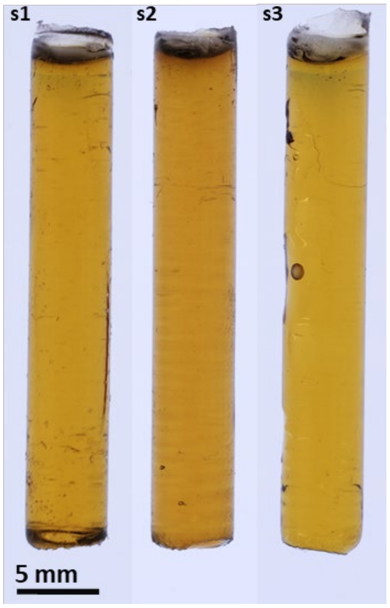

Figure S89: Image of triplicate of 25 mol% pNBE16-*co*-DCPD-H_2 at 1000:1:1 post-FROMP in DCPD-H_2:G2:TBP.

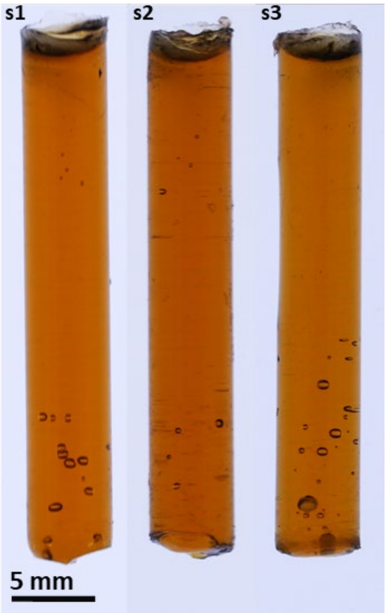

Figure S90: Image of triplicate of 25 mol% pNBE16-*co*-DCPD-H_2 at 500:1:1 post-FROMP in DCPD-H_2:G2:TBP.

Size-Exclusion Chromatography (SEC):

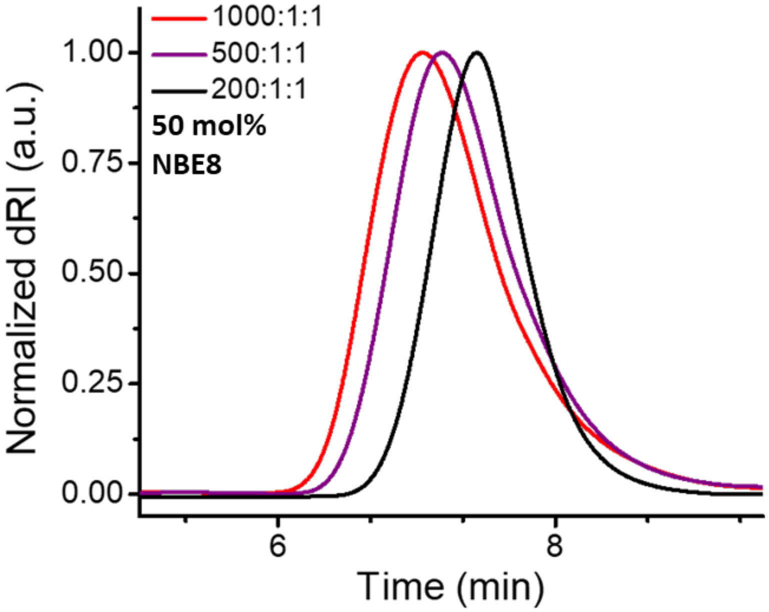

Figure S91: Representative SEC overlay of 50 mol% pNBE8-*co*-DCPD-H_2 post-FROMP for varied loadings (x:1:1 monomer:initiator:inhibitor).

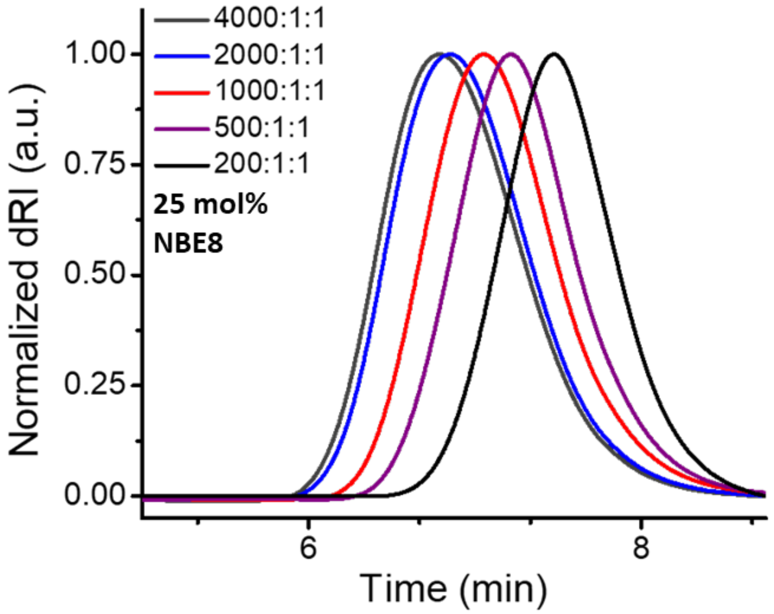

Figure S92: Representative SEC overlay of 25 mol% pNBE8-*co*-DCPD-H_2 post-FROMP for varied loadings (x:1:1 monomer:initiator:inhibitor).

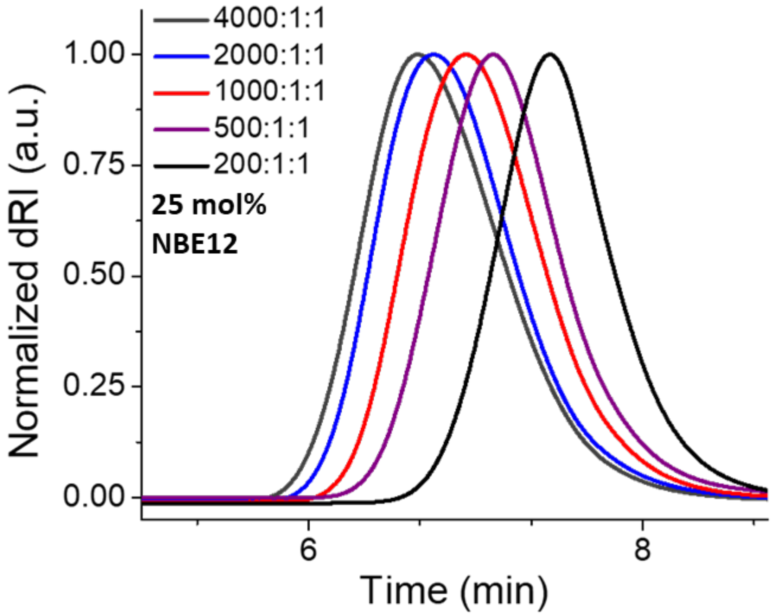

Figure S93: Representative SEC overlay of 25 mol% pNBE12-*co*-DCPD-H_2 post-FROMP for varied loadings (x:1:1 monomer:initiator:inhibitor).

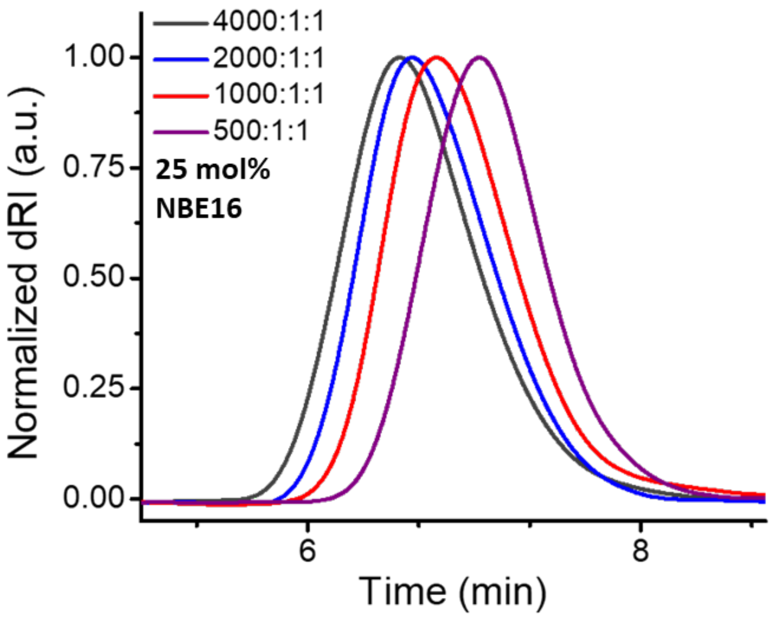

Figure S94: Representative SEC overlay of 25 mol% pNBE16-*co*-DCPD-H_2 post-FROMP for varied loadings (x:1:1 monomer:initiator:inhibitor).

Table S3: Degrees of polymerization and dispersity values (n = 3) determined by SEC for pNBE-*co*-DCPD-H_2 post-FROMP at varying loadings and molar incorporation of NBE monomer.

Monomer (equiv)	Initiator (equiv)	Inhibitor (equiv)	Molar incorporation of NBE monomer (%)	DP	error	*Đ*	error
NBE8							
1000	1	1	50	564	28	1.66	0.06
500	1	1	50	412	21	1.59	0.02
200	1	1	50	206	21	1.63	0.05
NBE8							
4000	1	1	25	1039	134	1.61	0.06
2000	1	1	25	784	17	1.58	0.02
1000	1	1	25	649	26	1.53	0.04
500	1	1	25	455	5	1.42	0.02
200	1	1	25	284	14	1.36	0.03
NBE12							
4000	1	1	25	959	19	1.59	0.05
2000	1	1	25	814	12	1.53	0.03
1000	1	1	25	683	47	1.45	0.03
500	1	1	25	473	10	1.47	0.02
200	1	1	25	263	5	1.35	0.01
NBE16							
4000	1	1	25	1414	99	1.53	0.05
2000	1	1	25	1048	61	1.55	0.08
1000	1	1	25	884	22	1.40	0.01
500	1	1	25	589	18	1.41	0.01

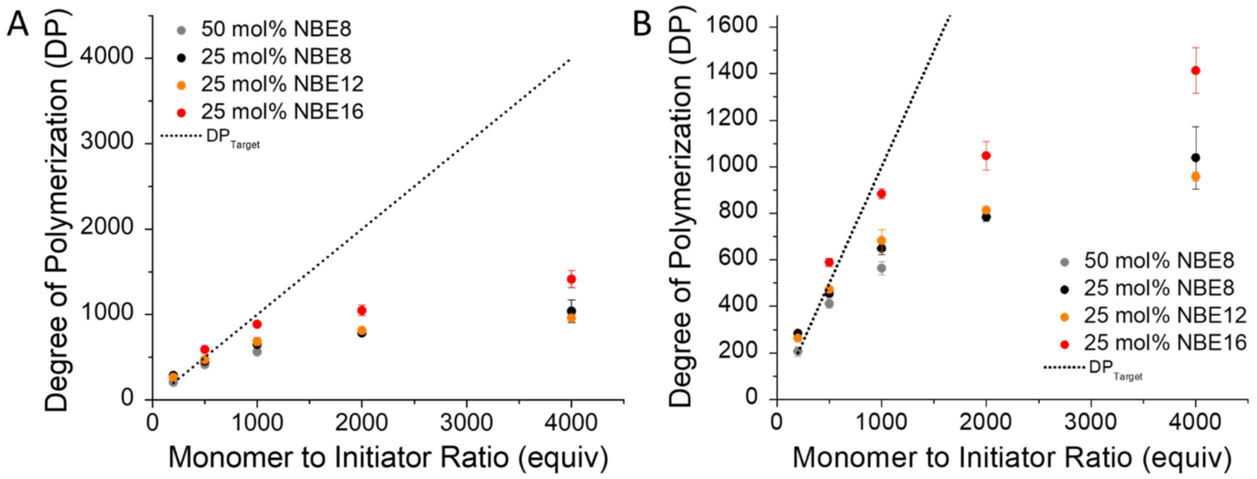

Figure S95: (A) Experimental degree of polymerization (DP) versus monomer to initiator ratio of pNBE-*co*-DCPD-H_2 post-FROMP at varying loadings and molar incorporation of NBE monomer. (B) Magnification of DP versus monomer to initiator ratio.

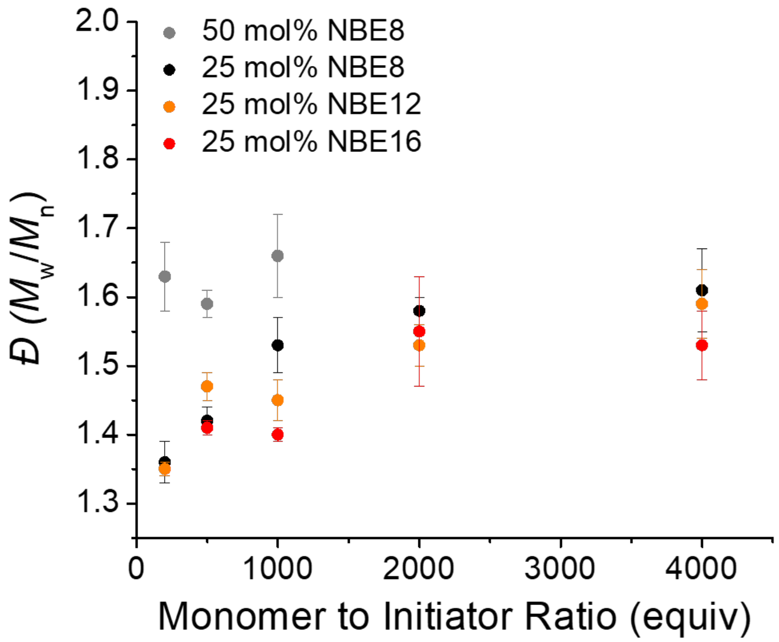

Figure S96: Dispersity (*Đ*) values of pNBE-*co*-DCPD-H_2 post-FROMP at varying loadings and molar incorporation of NBE monomer

Dynamic Scanning Calorimetry (DSC) Post-FROMP:

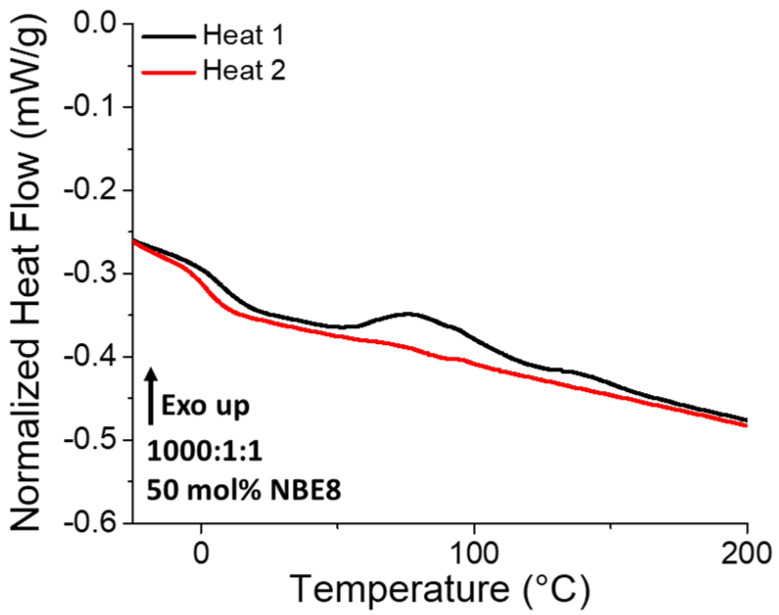

Figure S97: Representative DSC of 50 mol% pNBE8-*co*-DCPD-H_2 post-FROMP for 1000:1:1 (T_g = 2.6 ± 0.3 °C, $H_{r,\ residual}$ = 42 ± 28 J/g). First (black) and second heat (red) cycle. T_g is taken from the second heat cycle.

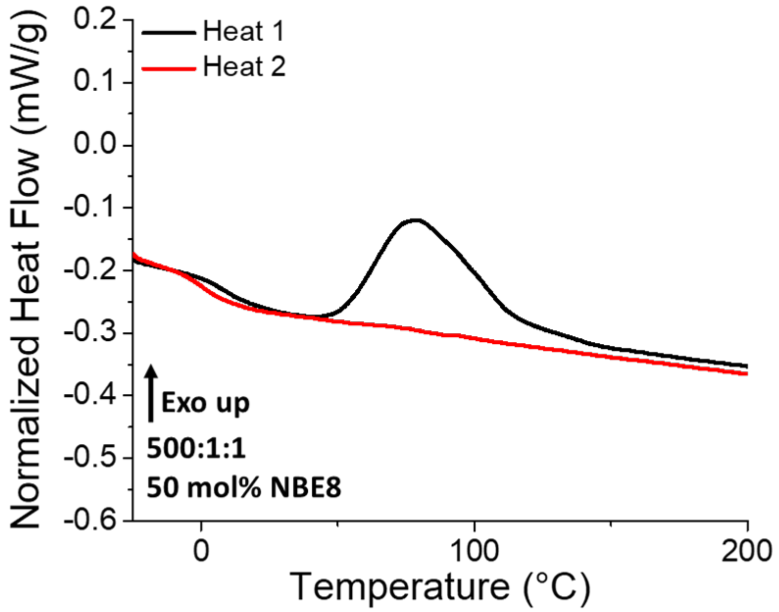

Figure S98: Representative DSC of 50 mol% pNBE8-*co*-DCPD-H_2 post-FROMP for 500:1:1 (T_g = 1.6 ± 0.8 °C, $H_{r,\ residual}$ = 50 ± 5 J/g). First (black) and second heat (red) cycle. T_g is taken from the second heat cycle.

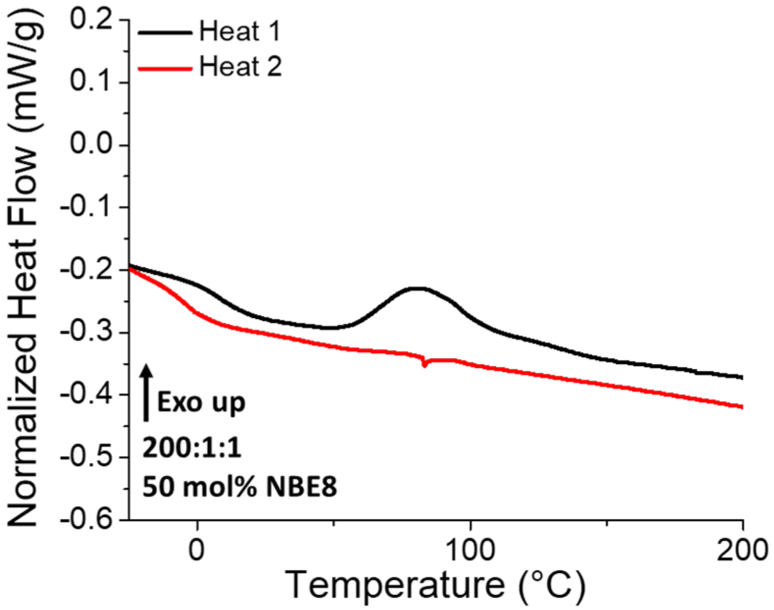

Figure S99: Representative DSC of 50 mol% pNBE8-*co*-DCPD-H_2 post-FROMP for 200:1:1 (T_g = -3.0 ± 0.6 °C, $H_{r,\ residual}$ = 40 ± 22 J/g). First (black) and second heat (red) cycle. T_g is taken from the second heat cycle.

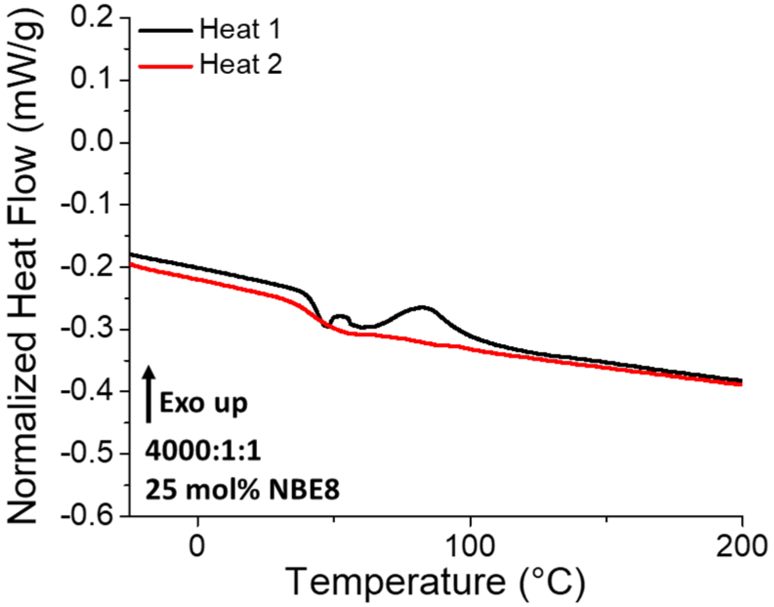

Figure S100: Representative DSC of 25 mol% pNBE8-*co*-DCPD-H_2 post-FROMP for 4000:1:1 (T_g = 42 ± 1 °C, $H_{r,\ residual}$ = 5 ± 1 J/g). First (black) and second heat (red) cycle. T_g is taken from the second heat cycle.

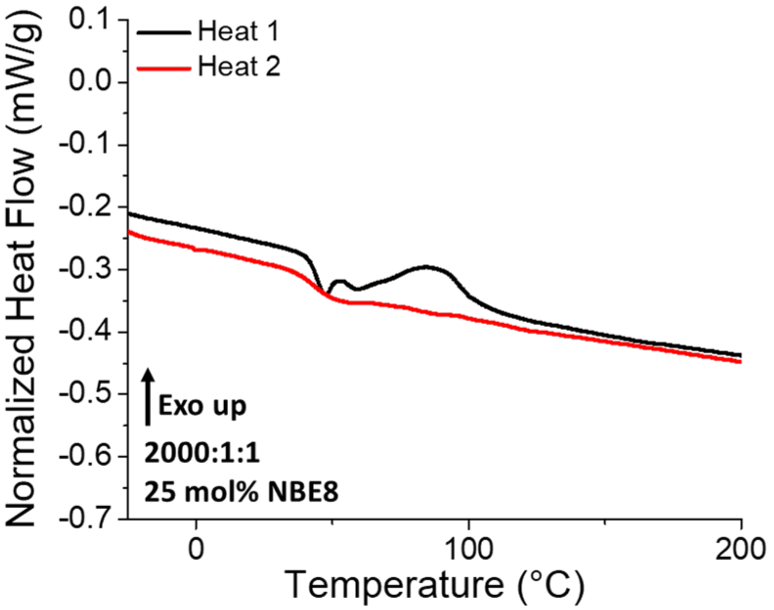

Figure S101: Representative DSC of 25 mol% pNBE8-*co*-DCPD-H_2 post-FROMP for 2000:1:1 (T_g = 43 ± 1 °C, $H_{r,\ residual}$ = 7 ± 2 J/g). First (black) and second heat (red) cycle. T_g is taken from the second heat cycle.

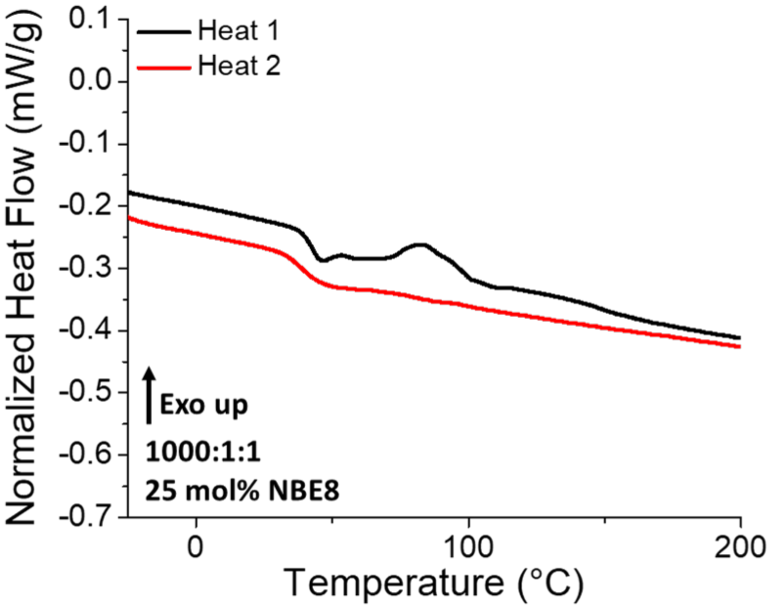

Figure S102: Representative DSC of 25 mol% pNBE8-*co*-DCPD-H_2 post-FROMP for 1000:1:1 (T_g = 39 ± 1 °C, $H_{r,\ residual}$ = 9 ± 3 J/g). First (black) and second heat (red) cycle. T_g is taken from the second heat cycle.

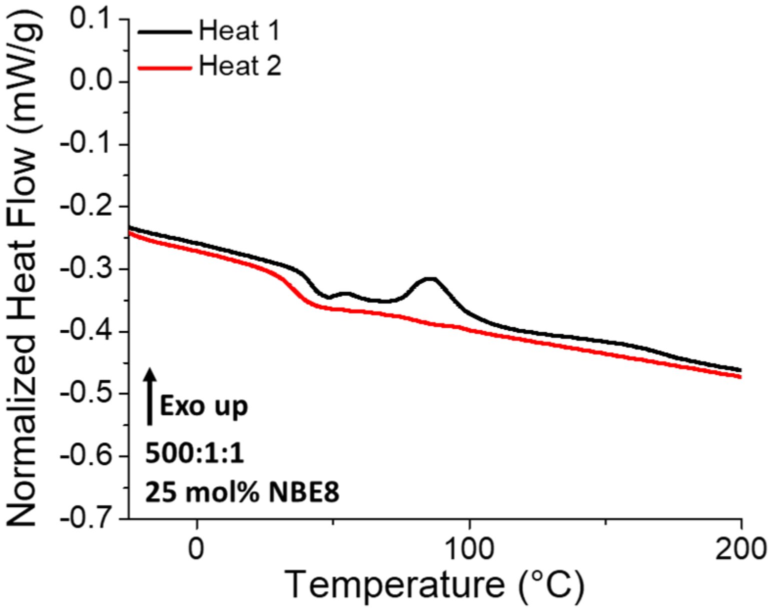

Figure S103: Representative DSC of 25 mol% pNBE8-*co*-DCPD-H_2 post-FROMP for 500:1:1 (T_g = 35 ± 2 °C, $H_{r,\ residual}$ = 10 ± 2 J/g). First (black) and second heat (red) cycle. T_g is taken from the second heat cycle.

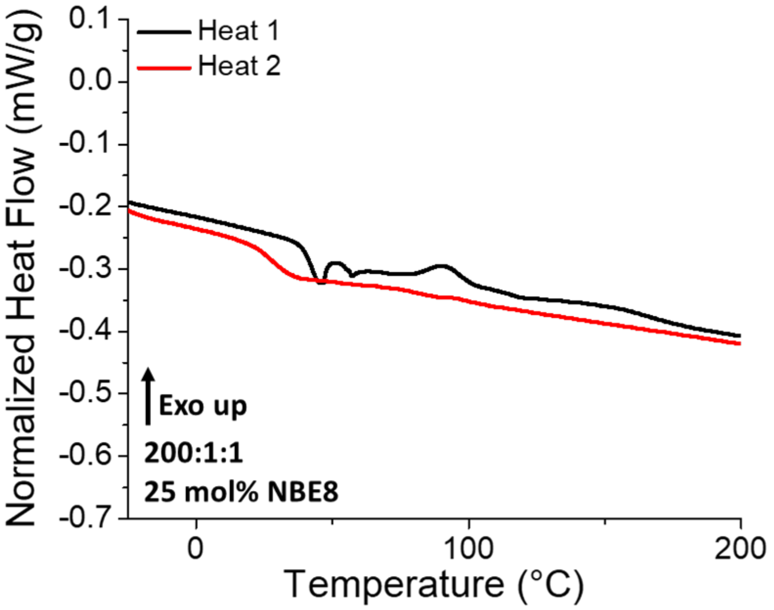

Figure S104: Representative DSC of 25 mol% pNBE8-*co*-DCPD-H_2 post-FROMP for 200:1:1 (T_g = 27 ± 1 °C, $H_{r,\ residual}$ = 5 ± 3 J/g). First (black) and second heat (red) cycle. T_g is taken from the second heat cycle.

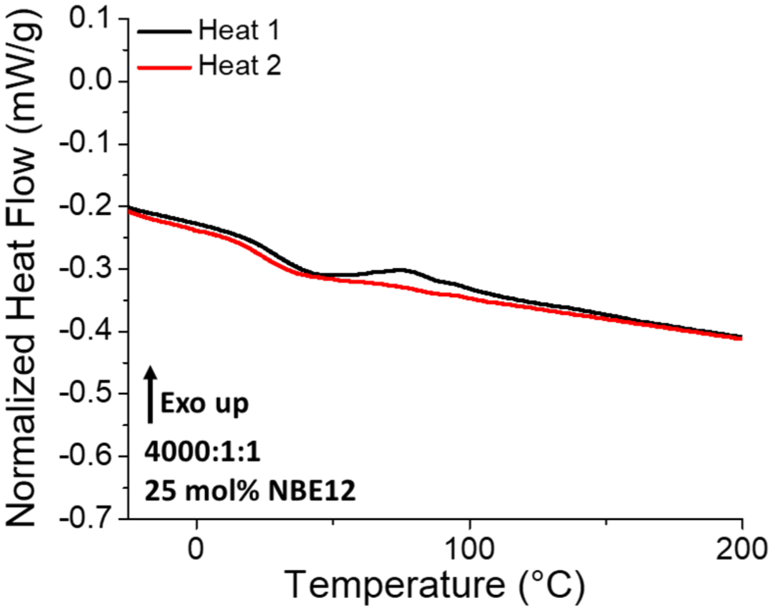

Figure S105: Representative DSC of 25 mol% pNBE12-*co*-DCPD-H_2 post-FROMP for 4000:1:1 (T_g = 25 ± 1 °C, $H_{r,\ residual}$ = 8 ± 1 J/g). First (black) and second heat (red) cycle. T_g is taken from the second heat cycle.

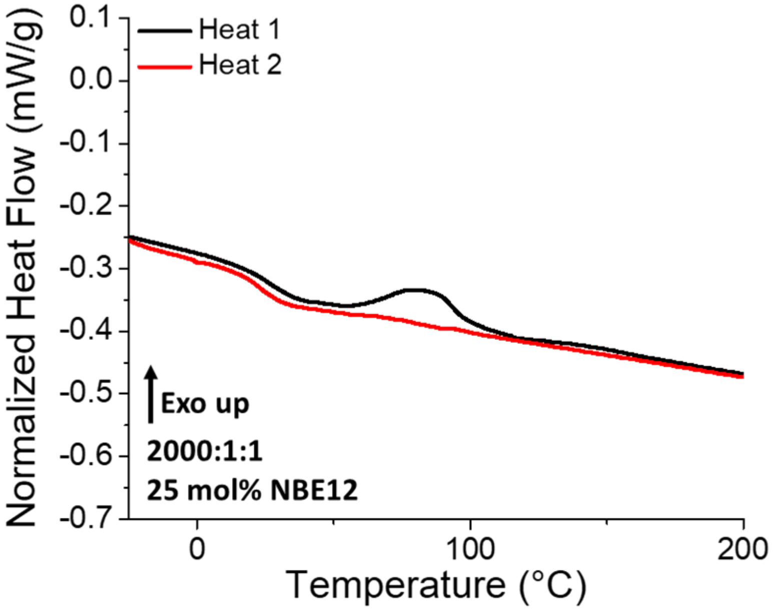

Figure S106: Representative DSC of 25 mol% pNBE12-*co*-DCPD-H_2 post-FROMP for 2000:1:1 (T_g = 23 ± 1 °C, $H_{r,\ residual}$ = 8 ± 1 J/g). First (black) and second heat (red) cycle. T_g is taken from the second heat cycle.

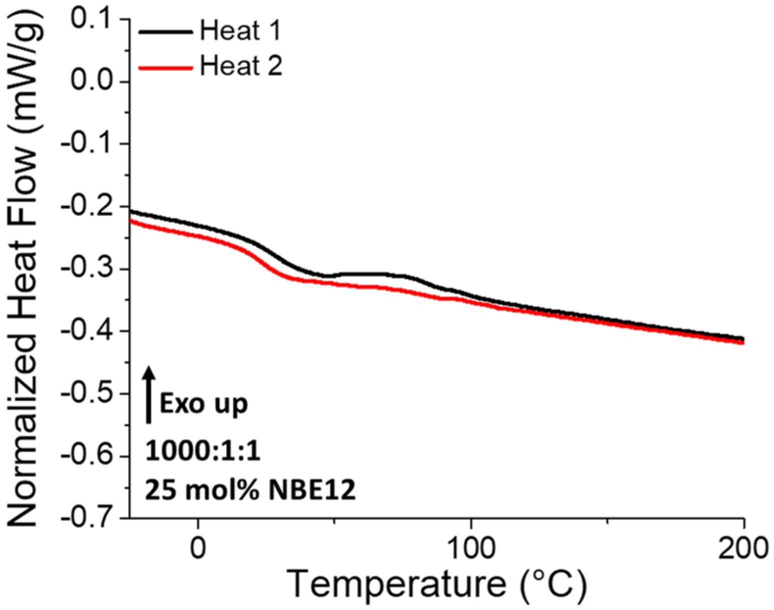

Figure S107: Representative DSC of 25 mol% pNBE12-*co*-DCPD-H_2 post-FROMP for 1000:1:1 (T_g = 22 ± 2 °C, $H_{r,\ residual}$ = 8 ± 4 J/g). First (black) and second heat (red) cycle. T_g is taken from the second heat cycle.

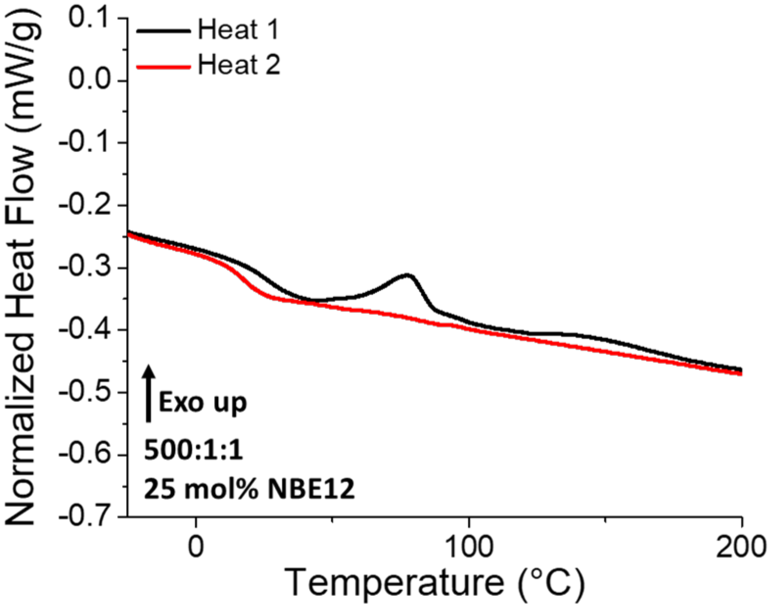

Figure S108: Representative DSC of 25 mol% pNBE12-*co*-DCPD-H_2 post-FROMP for 500:1:1 (T_g = 18 ± 1 °C, $H_{r,\ residual}$ =11 ± 1 J/g). First (black) and second heat (red) cycle. T_g is taken from the second heat cycle.

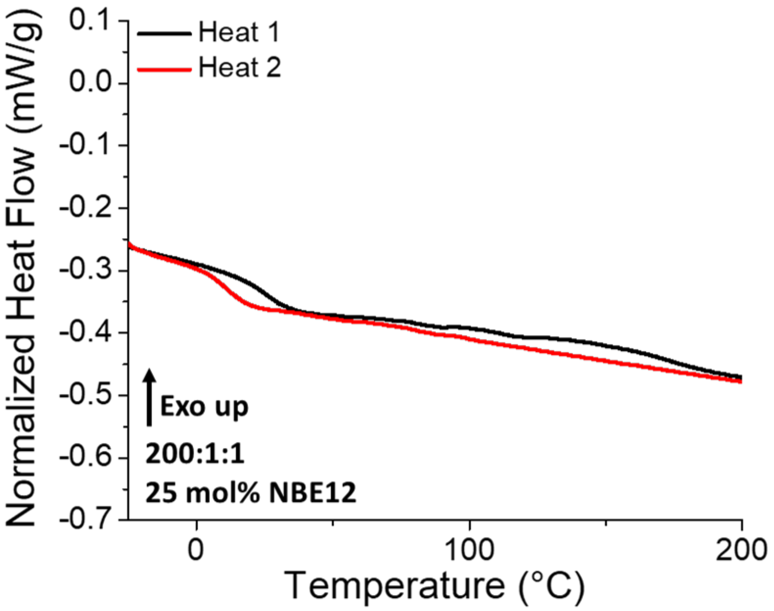

Figure S109: Representative DSC of 25 mol% pNBE12-*co*-DCPD-H_2 post-FROMP for 200:1:1 (T_g = 12 ± 1 °C, $H_{r,\ residual}$ = 13 ± 2 J/g). First (black) and second heat (red) cycle. T_g is taken from the second heat cycle.

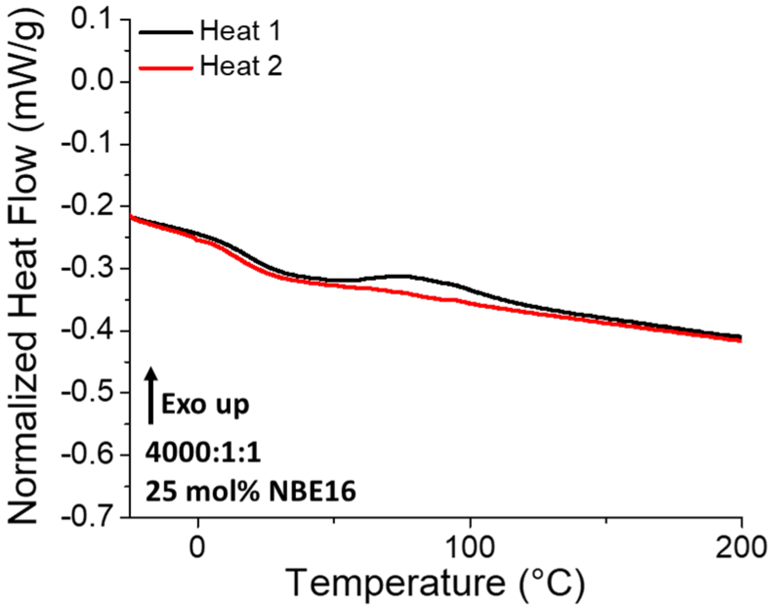

Figure S110: Representative DSC of 25 mol% pNBE16-*co*-DCPD-H_2 post-FROMP for 4000:1:1 (T_g = 16 ± 1 °C, $H_{r,\ residual}$ = 8 ± 2 J/g). First (black) and second heat (red) cycle. T_g is taken from the second heat cycle.

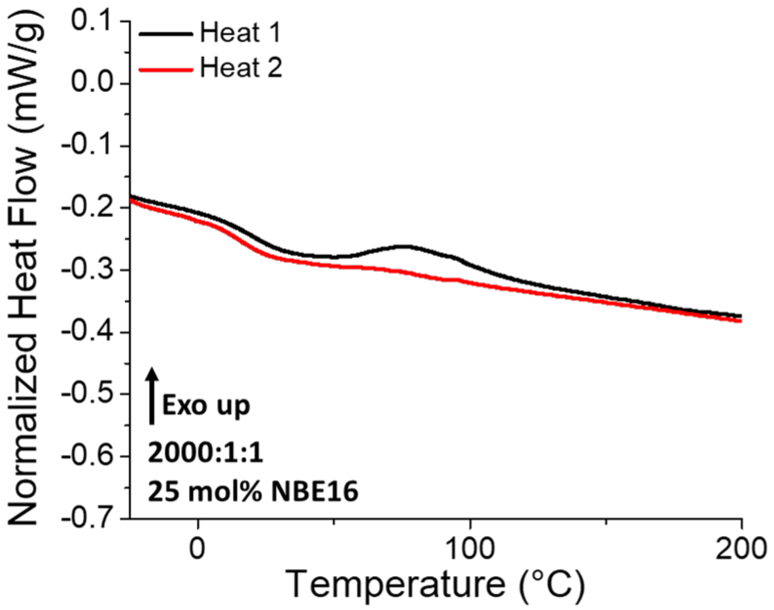

Figure S111: Representative DSC of 25 mol% pNBE16-*co*-DCPD-H_2 post-FROMP for 2000:1:1 (T_g = 16 ± 1 °C, $H_{r,\ residual}$ = 11 ± 2 J/g). First (black) and second heat (red) cycle. T_g is taken from the second heat cycle.

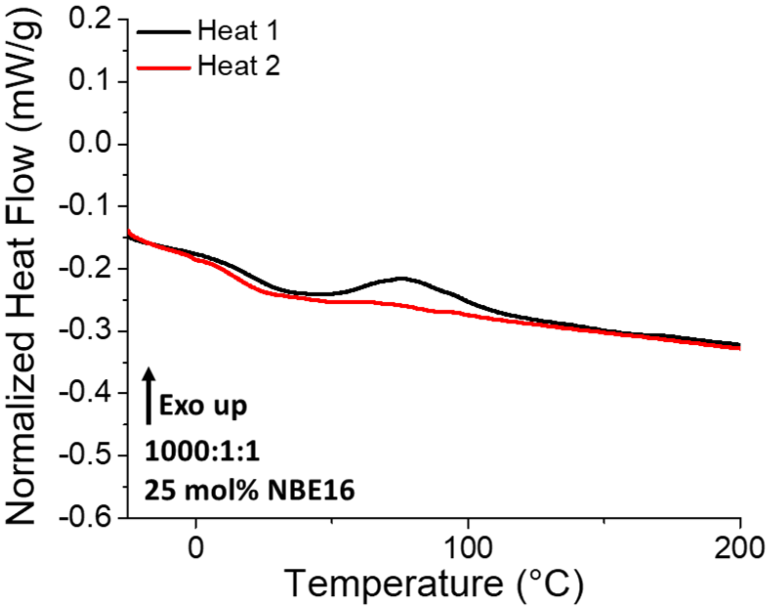

Figure S112: Representative DSC of 25 mol% pNBE16-*co*-DCPD-H_2 post-FROMP for 1000:1:1 (T_g = 14 ± 2 °C, $H_{r,\ residual}$ = 14 ± 3 J/g). First (black) and second heat (red) cycle. T_g is taken from the second heat cycle.

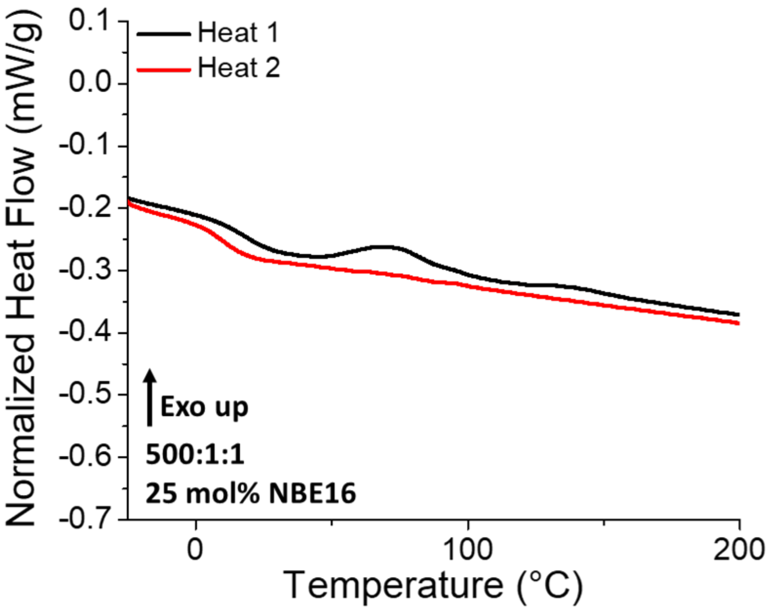

Figure S113: Representative DSC of 25 mol% pNBE16-*co*-DCPD-H_2 post-FROMP for 500:1:1 (T_g = 12 ± 1 °C, $H_{r,\ residual}$ = 9 ± 2 J/g). First (black) and second heat (red) cycle. T_g is taken from the second heat cycle.

Table S4: Glass transition temperature (T_g) for pNBE-*co*-DCPD-H_2 post-FROMP at varied loadings. T_g is taken from the second heat cycle.

**Monomer (equiv)**	**Initiator (equiv)**	**Inhibitor (equiv)**	**Molar incorporation of NBE monomer (%)**	**T_g (°C)**	**error**
NBE8					
1000	1	1	50	2.6	0.3
500	1	1	50	1.6	0.8
200	1	1	50	-3.0	0.6
NBE8					
4000	1	1	25	42	1
2000	1	1	25	43	1
1000	1	1	25	39	1
500	1	1	25	35	2
200	1	1	25	27	1
NBE12					
4000	1	1	25	25	8
2000	1	1	25	23	1
1000	1	1	25	22	2
500	1	1	25	18	1
200	1	1	25	12	1
NBE16					
4000	1	1	25	16	1
2000	1	1	25	16	1
1000	1	1	25	14	2
500	1	1	25	12	1

Table S5: Residual heat of reaction ($H_{r,\ residual}$) and percent monomer conversion for pDCPD-H_2 post-FROMP at varied loadings.

Monomer (equiv)	Initiator (equiv)	Inhibitor (equiv)	Molar incorporation of NBE monomer (%)	$H_{r,\ residual}$ (J/g)	error	Calculated Conversion (%)	error
NBE8							
1000	1	1	50	42	28	85	8
500	1	1	50	50	5	83	2
200	1	1	50	40	22	86	9
NBE8							
4000	1	1	25	5	2	99	1
2000	1	1	25	7	2	98	1
1000	1	1	25	9	3	97	1
500	1	1	25	10	2	97	1
200	1	1	25	5	3	99	1
NBE12							
4000	1	1	25	8	1	98	1
2000	1	1	25	8	1	98	1
1000	1	1	25	8	4	98	1
500	1	1	25	11	1	97	1
200	1	1	25	13	2	96	1
NBE16							
4000	1	1	25	8	2	98	1
2000	1	1	25	11	2	96	1
1000	1	1	25	14	3	95	1
500	1	1	25	9	2	97	1

NMR Spectroscopy Post-Cure:

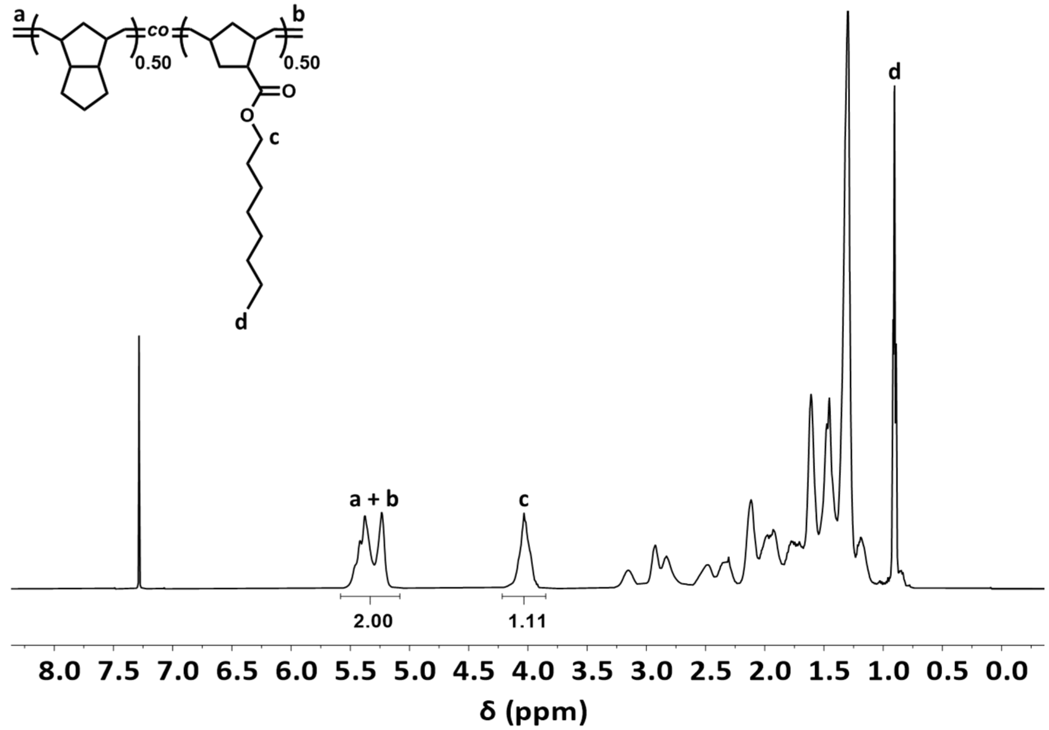

Figure S114: Representative NMR of 50 mol% pNBE8-*co*-DCPD-H_2 post-FROMP for 1000:1:1 with characteristic repeat unit signals labeled.

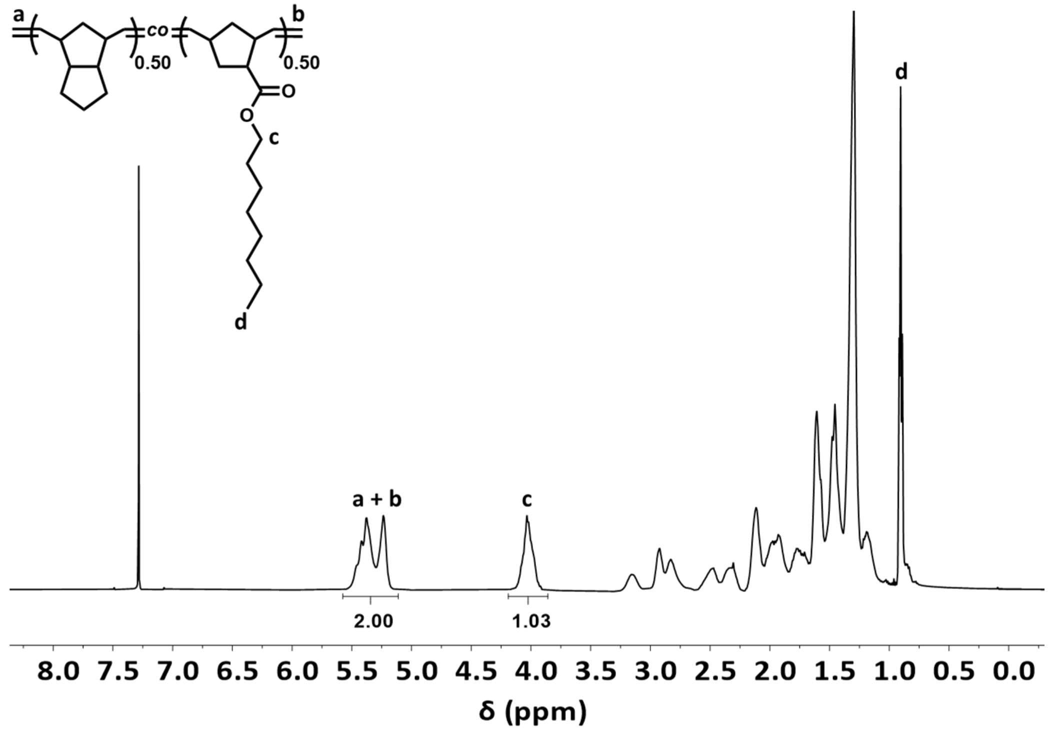

Figure S115: Representative NMR of 50 mol% pNBE8-*co*-DCPD-H_2 post-FROMP for 500:1:1 with characteristic repeat unit signals labeled.

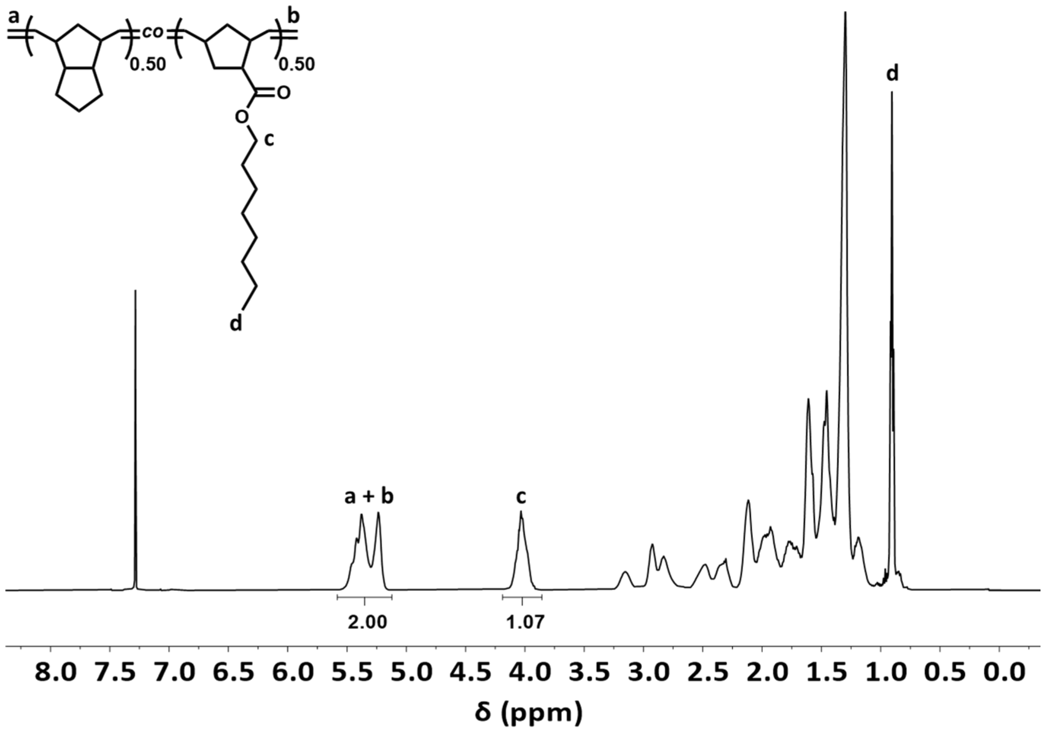

Figure S116: Representative NMR of 50 mol% pNBE8-*co*-DCPD-H_2 post-FROMP for 200:1:1 with characteristic repeat unit signals labeled.

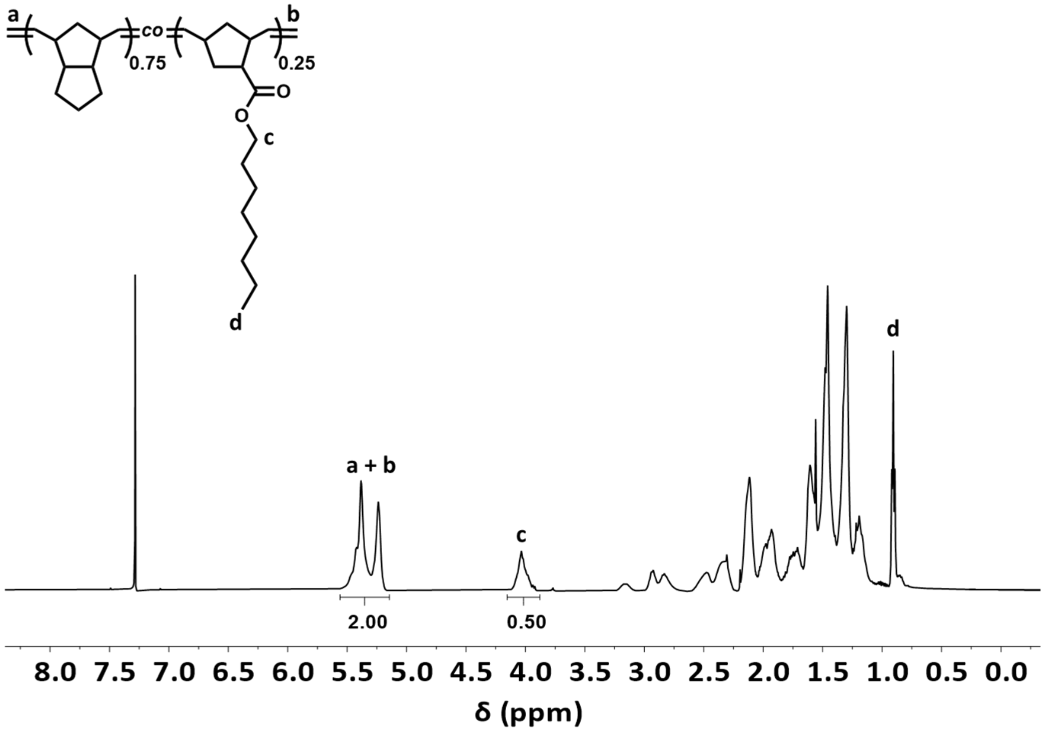

Figure S117: Representative NMR of 25 mol% pNBE8-*co*-DCPD-H_2 post-FROMP for 4000:1:1 with characteristic repeat unit signals labeled.

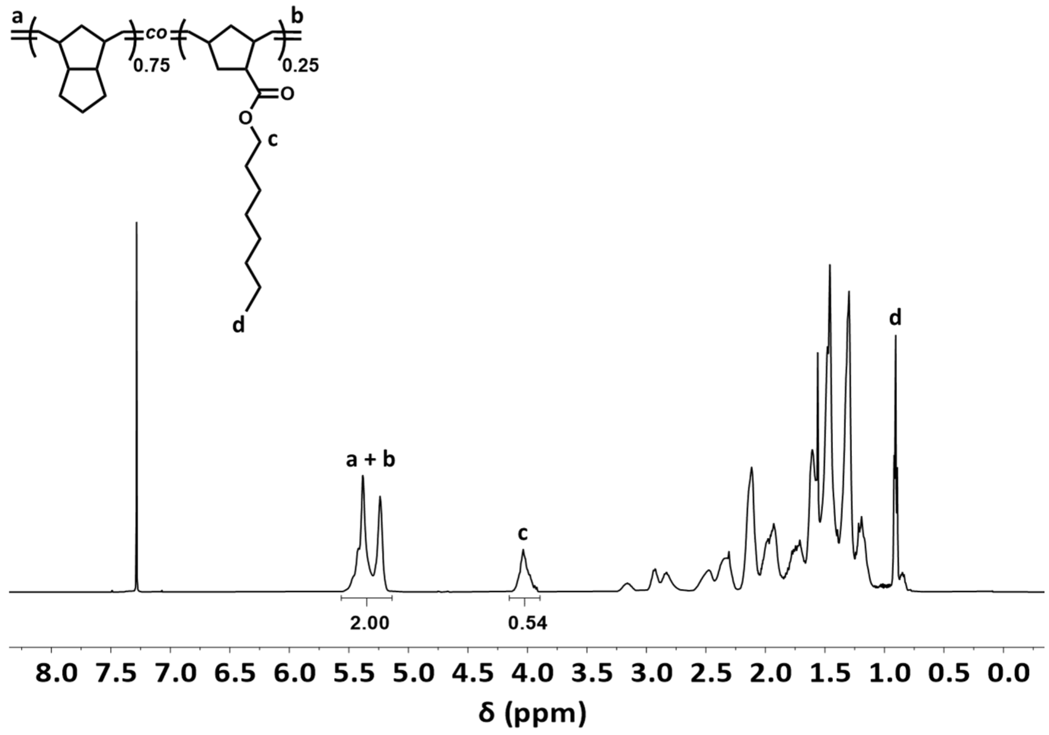

Figure S118: Representative NMR of 25 mol% pNBE8-*co*-DCPD-H_2 post-FROMP for 2000:1:1 with characteristic repeat unit signals labeled.

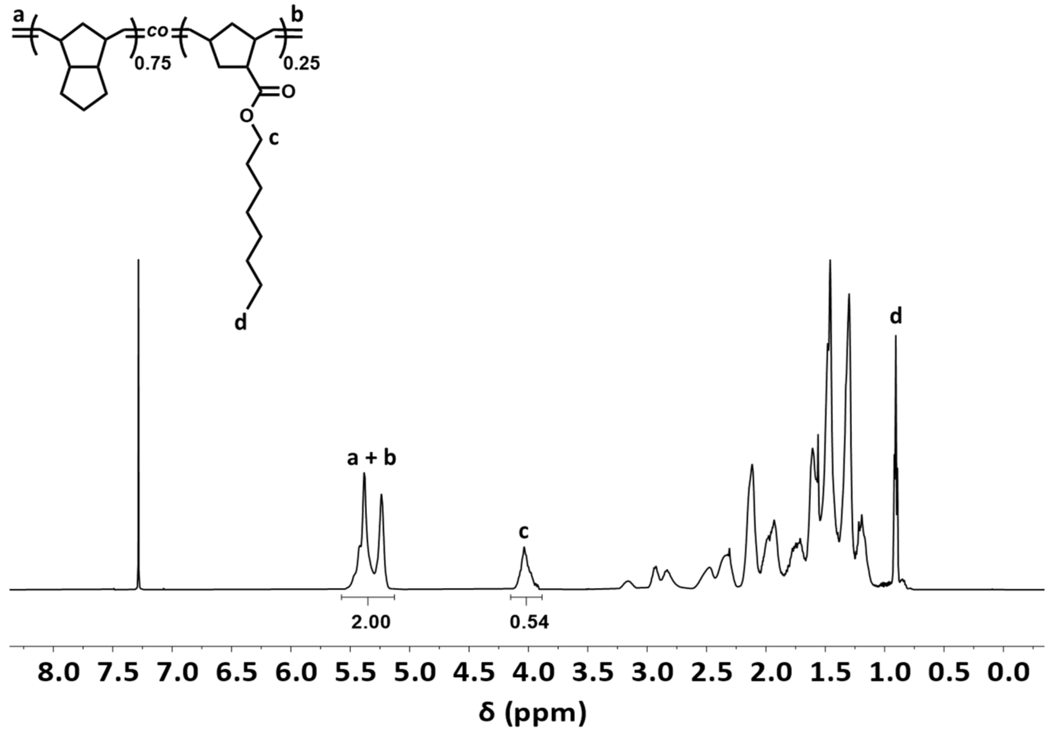

Figure S119: Representative NMR of 25 mol% pNBE8-*co*-DCPD-H_2 post-FROMP for 1000:1:1 with characteristic repeat unit signals labeled.

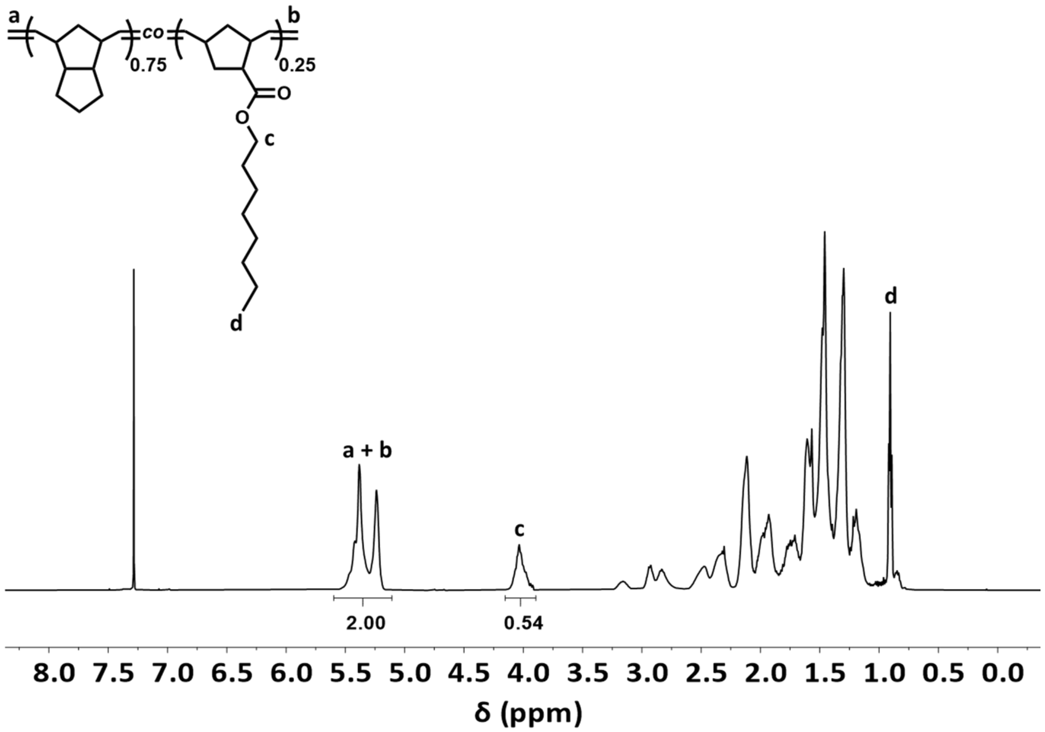

Figure S120: Representative NMR of 25 mol% pNBE8-*co*-DCPD-H_2 post-FROMP for 500:1:1 with characteristic repeat unit signals labeled.

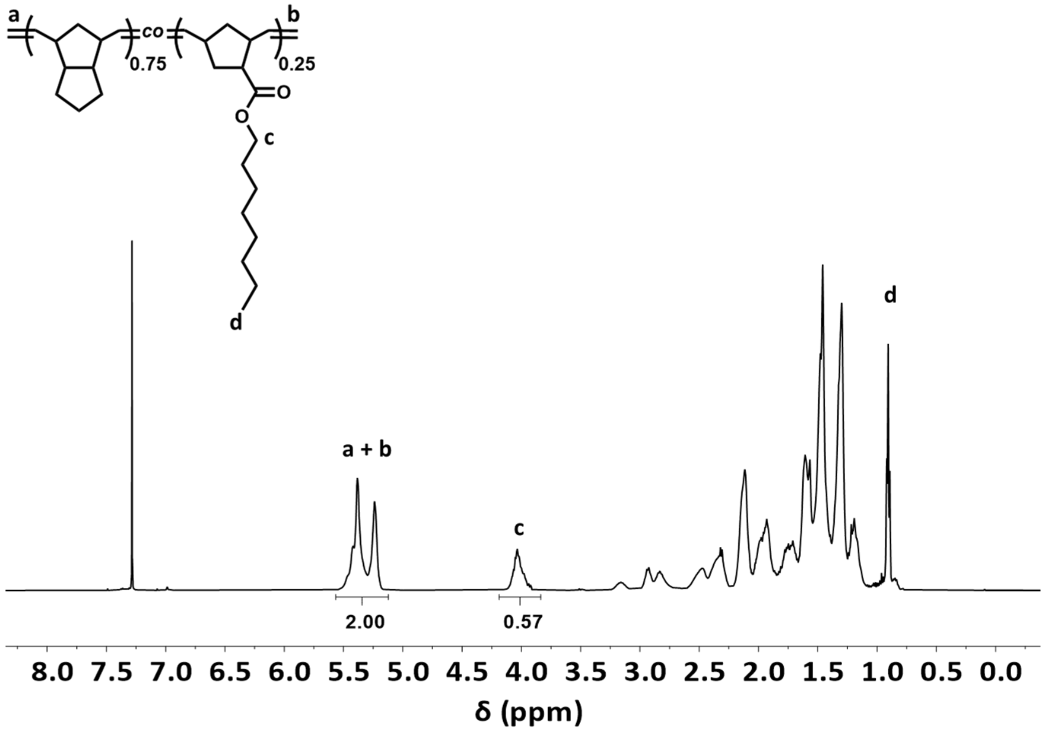

Figure S121: Representative NMR of 25 mol% pNBE8-*co*-DCPD-H_2 post-FROMP for 200:1:1 with characteristic repeat unit signals labeled.

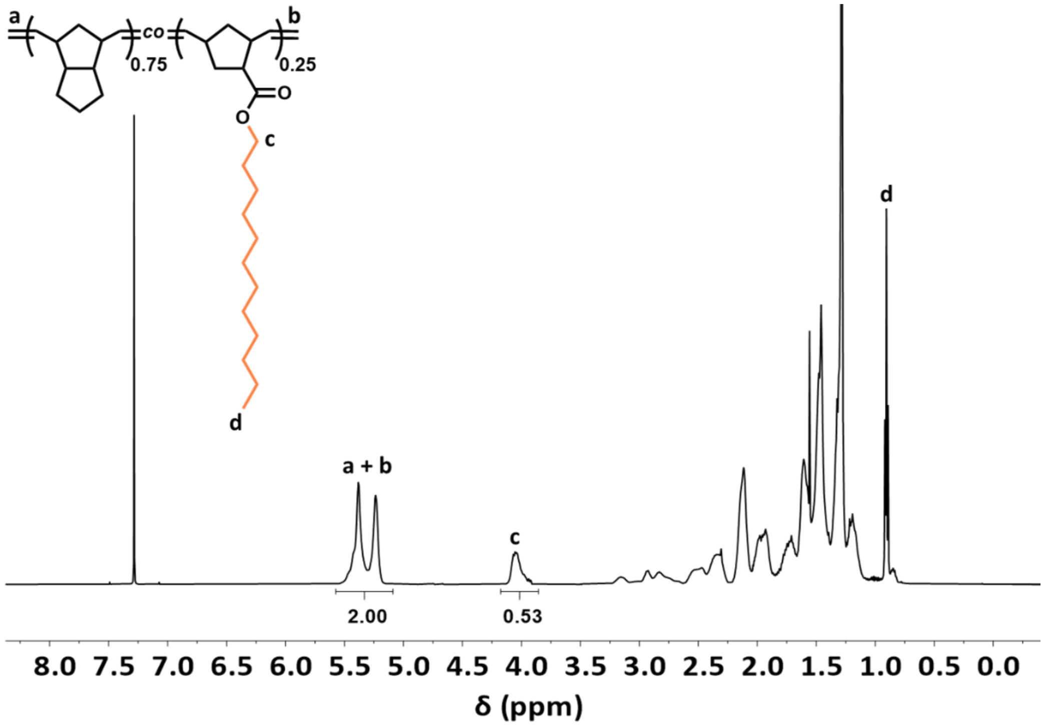

Figure S122: Representative NMR of 25 mol% pNBE12-*co*-DCPD-H_2 post-FROMP for 4000:1:1 with characteristic repeat unit signals labeled.

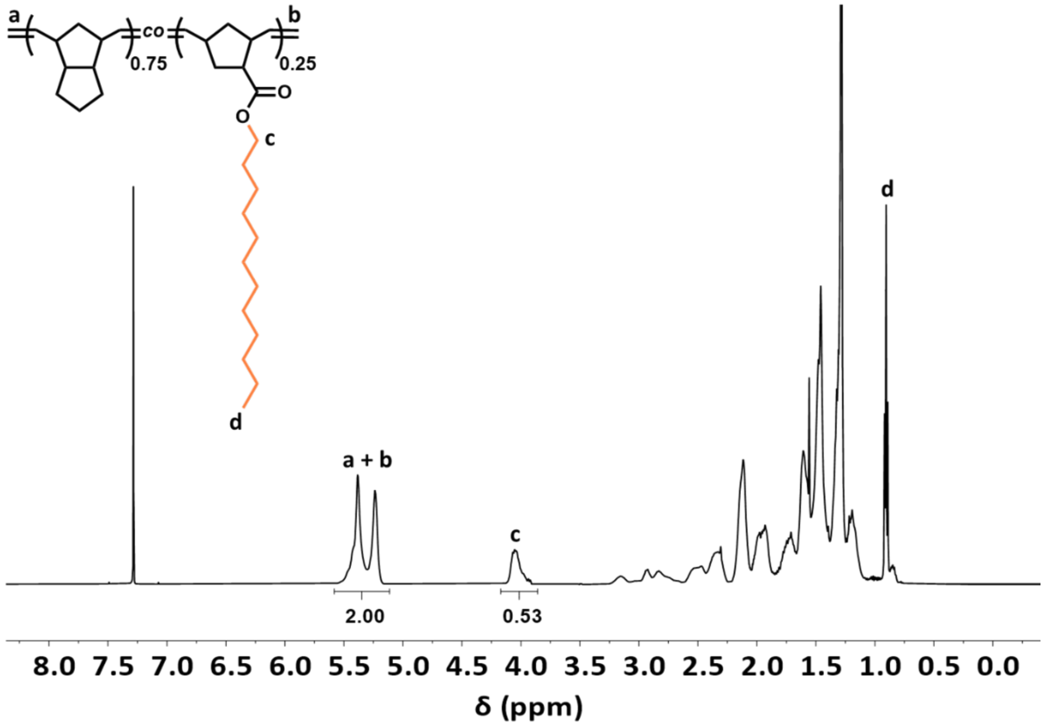

Figure S123: Representative NMR of 25 mol% pNBE12-*co*-DCPD-H_2 post-FROMP for 2000:1:1 with characteristic repeat unit signals labeled.

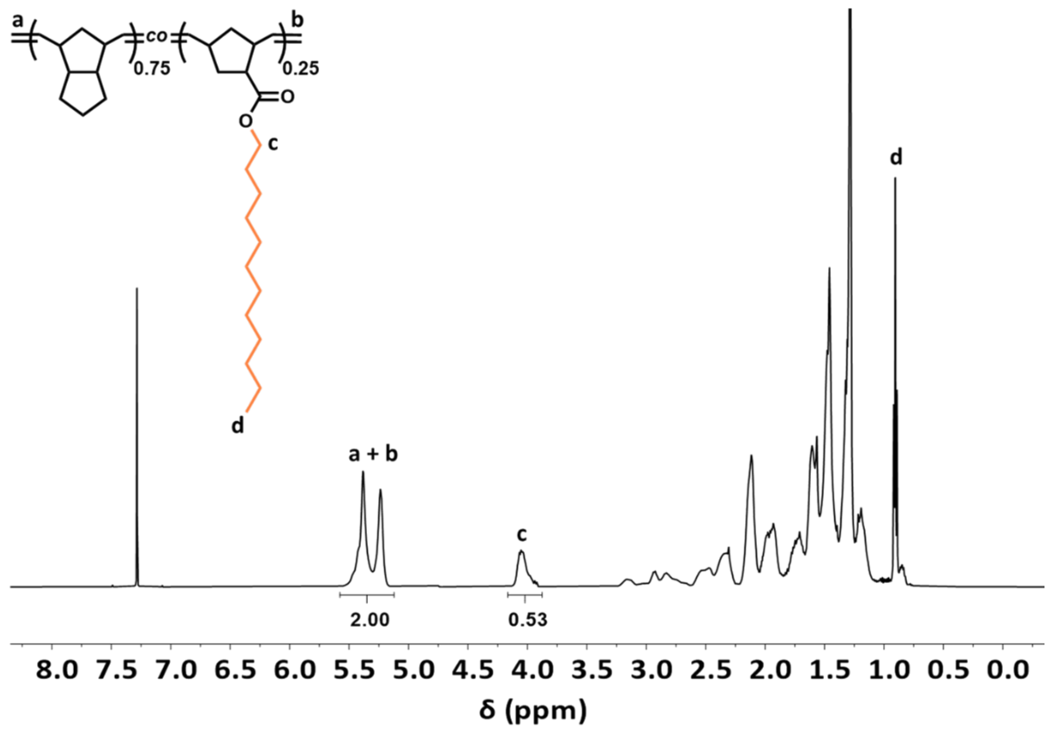

Figure S124: Representative NMR of 25 mol% pNBE12-*co*-DCPD-H_2 post-FROMP for 1000:1:1 with characteristic repeat unit signals labeled.

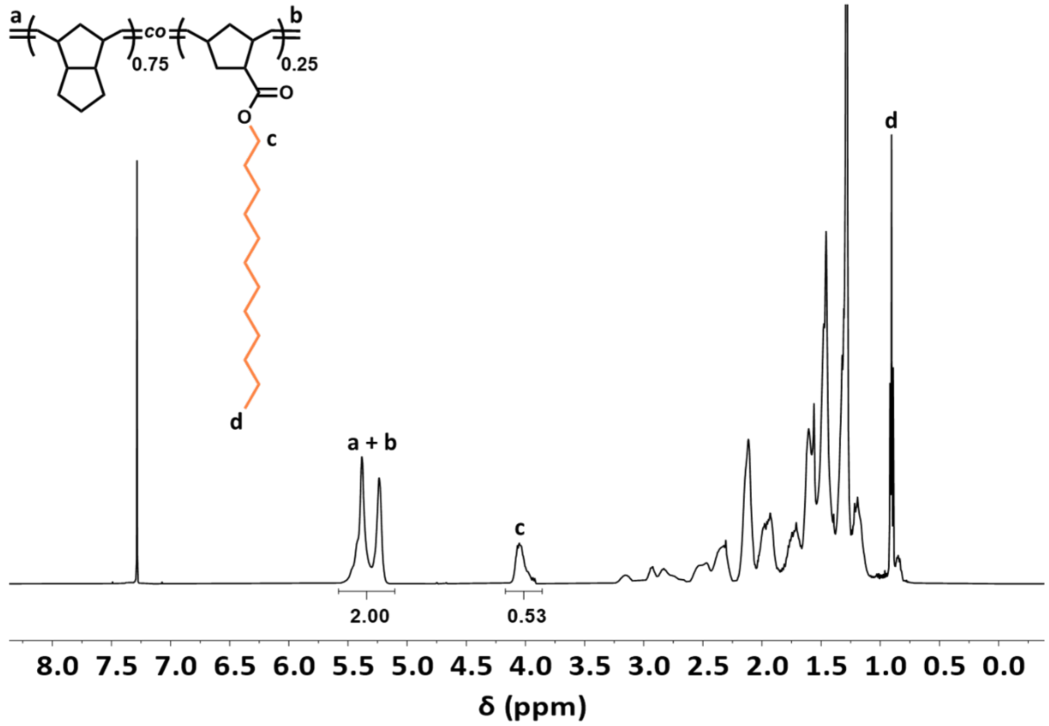

Figure S125: Representative NMR of 25 mol% pNBE12-*co*-DCPD-H_2 post-FROMP for 500:1:1 with characteristic repeat unit signals labeled.

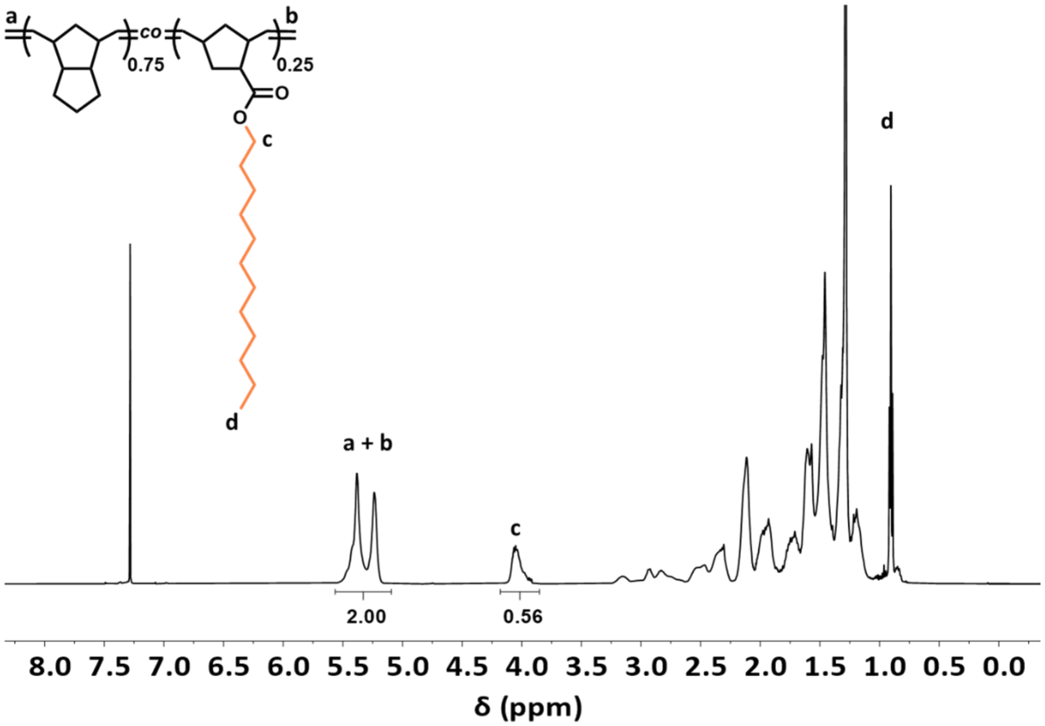

Figure S126: Representative NMR of 25 mol% pNBE12-*co*-DCPD-H_2 post-FROMP for 200:1:1 with characteristic repeat unit signals labeled.

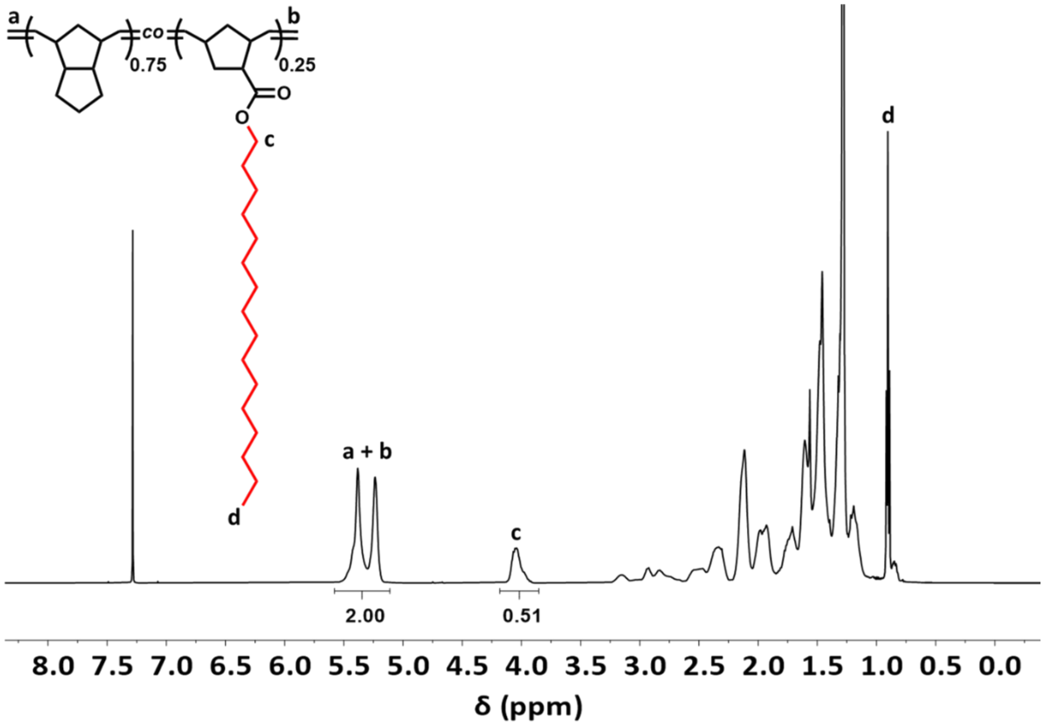

Figure S127: Representative NMR of 25 mol% pNBE16-*co*-DCPD-H_2 post-FROMP for 4000:1:1 with characteristic repeat unit signals labeled.

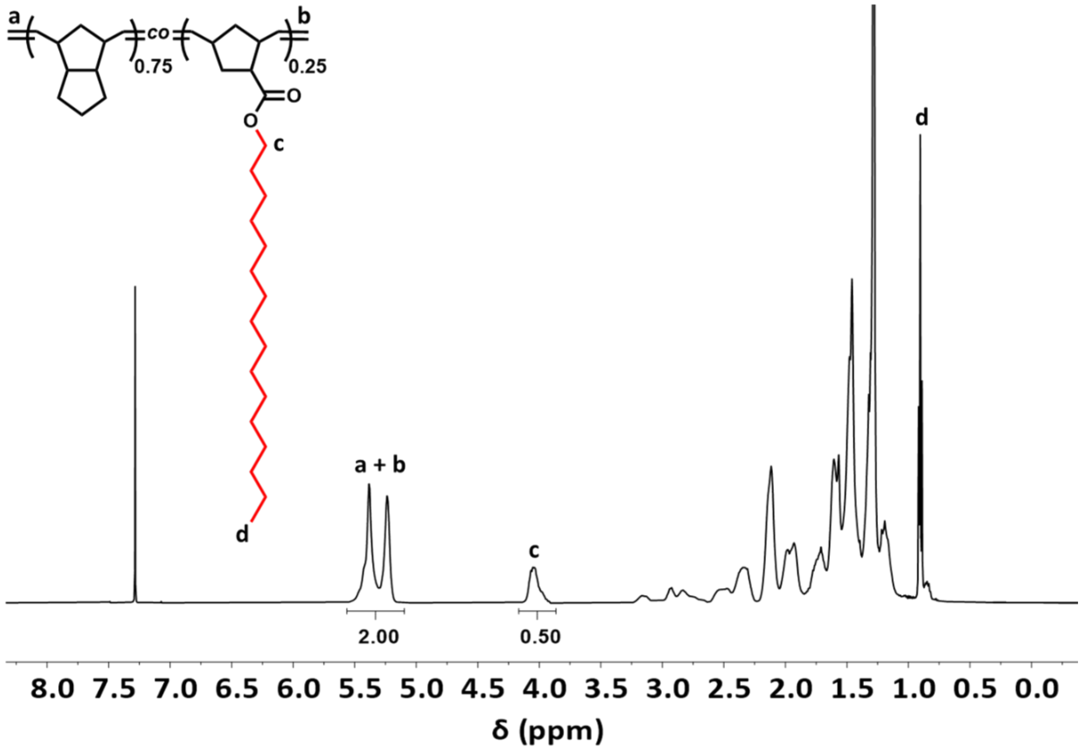

Figure S128: Representative NMR of 25 mol% pNBE16-*co*-DCPD-H_2 post-FROMP for 2000:1:1 with characteristic repeat unit signals labeled.

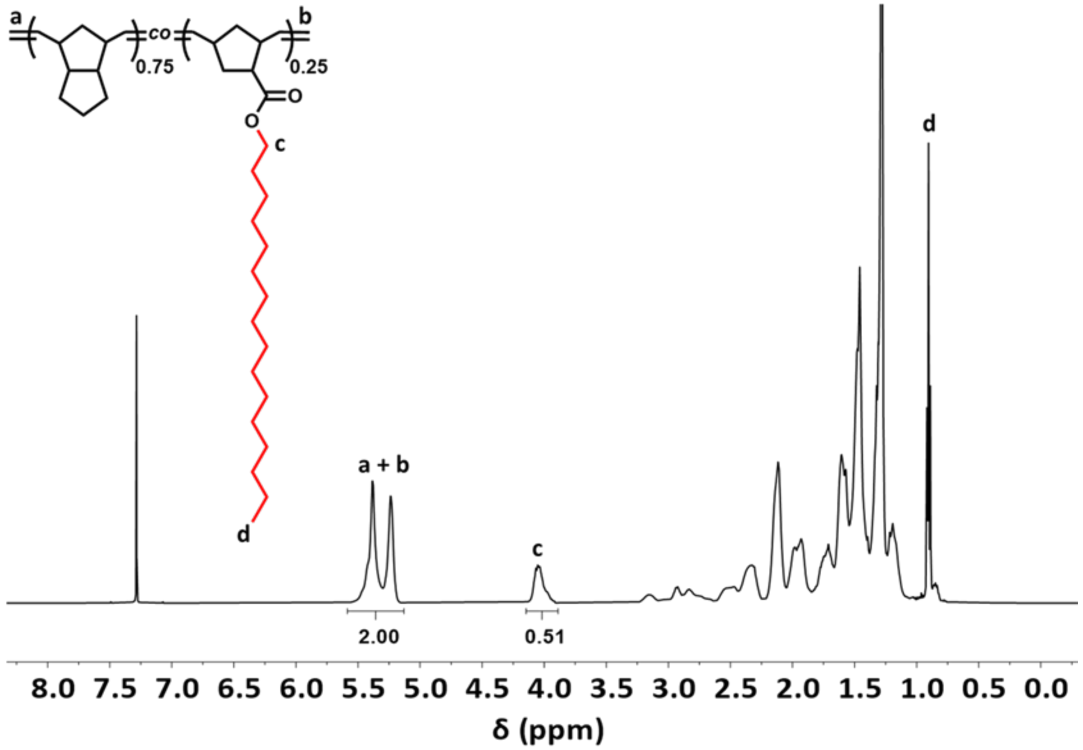

Figure S129: Representative NMR of 25 mol% pNBE16-*co*-DCPD-H_2 post-FROMP for 1000:1:1 with characteristic repeat unit signals labeled.

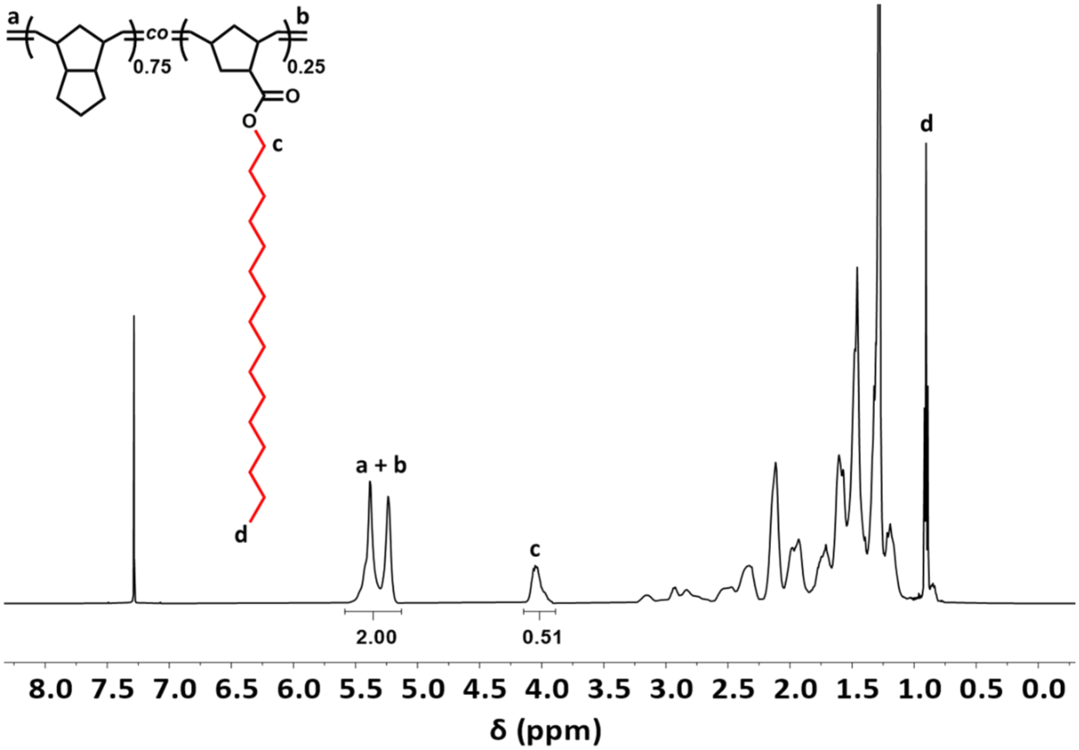

Figure S130: Representative NMR of 25 mol% pNBE16-*co*-DCPD-H_2 post-FROMP for 500:1:1 with characteristic repeat unit signals labeled.

Table S6: Ester incorporation (%) post-FROMP determined by NMR for 1000:1:1 (n = 3).

Monomer (equiv)	Initiator (equiv)	Inhibitor (equiv)	Molar incorporation, Theory (%)	Molar incorporation, NMR (%)	error
NBE8					
1000	1	1	50	55.0	1.5
NBE8					
1000	1	1	25	26.5	0.7
NBE12					
1000	1	1	25	26.0	1.1
NBE16					
1000	1	1	25	25.0	0.7

General Procedure for NBE and DCPD Resin Preparation:

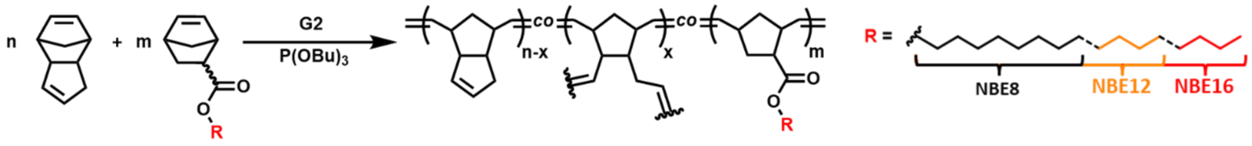

Grubbs' 2nd generation initiator was massed (G2, **w** mg, 1.00 equiv.) in a 5 mL vial prior to the addition of tributyl phosphite (TBP, **x** μL, **y** equiv.). The mixture was dissolved in monomer (*NBE and DCPD*, 2.800 g, **z** equiv.) and sonicated for up to 5 minutes. The resulting solution was transferred to 3 mm rubber gasket U-mold in a 40 °C oven. After a 2-3 min soak period, U-mold samples were initiated at the top the resin with a preheated sottering iron creating a descending front.

<u>50 mol% NBE8; n = 5</u>

<u>1000:1:1 Monomer:Initiator:Inhibitor</u>

G2: **w** = 12.44 mg

TBP: **x** = 4.00 μL, **y** = 1.00 equiv

NBE8 and DCPD: **z** = 500 and 500 equiv, respectively

<u>25 mol% NBE8; n = 5</u>

<u>1000:1:1 Monomer:Initiator:Inhibitor</u>

G2: **w** = 14.70 mg

TBP: **x** = 4.70 μL, **y** = 1.00 equiv

NBE8 and DCPD: **z** = 250 and 750 equiv, respectively

<u>25 mol% NBE12; n = 5</u>

<u>1000:1:1 Monomer:Initiator:Inhibitor</u>

G2: **w** = 13.52 mg

TBP: **x** = 4.30 μL, **y** = 1.00 equiv.

NBE12 and DCPD: **z** = 250 and 750 equiv, respectively

<u>25 mol% NBE16; n = 5</u>

<u>1000:1:1 Monomer:Initiator:Inhibitor</u>

G2: **w** = 12.52 mg

TBP: **x** = 4.00 μL, **y** = 1.00 equiv.

NBE16 and DCPD: **z** = 250 and 750 equiv, respectively

50 mol% NBE8 (linear); n = 3

1000:1:1 Monomer:Initiator:Inhibitor

G2: **w** = 12.36 mg

TBP: **x** = 3.95 µL, **y** = 1.00 equiv.

NBE8 and DCPD-H_2: **z** = 500 and 500 equiv, respectively

25 mol% NBE16 (linear); n = 3

1000:1:1 Monomer:Initiator:Inhibitor

G2: **w** = 12.39 mg

TBP: **x** = 4.00 µL, **y** = 1.00 equiv.

NBE8 and DCPD-H_2: **z** = 250 and 750 equiv, respectively

Front velocities and Timelapses (3 mm U-molds at 40 °C):

50 mol% NBE8:

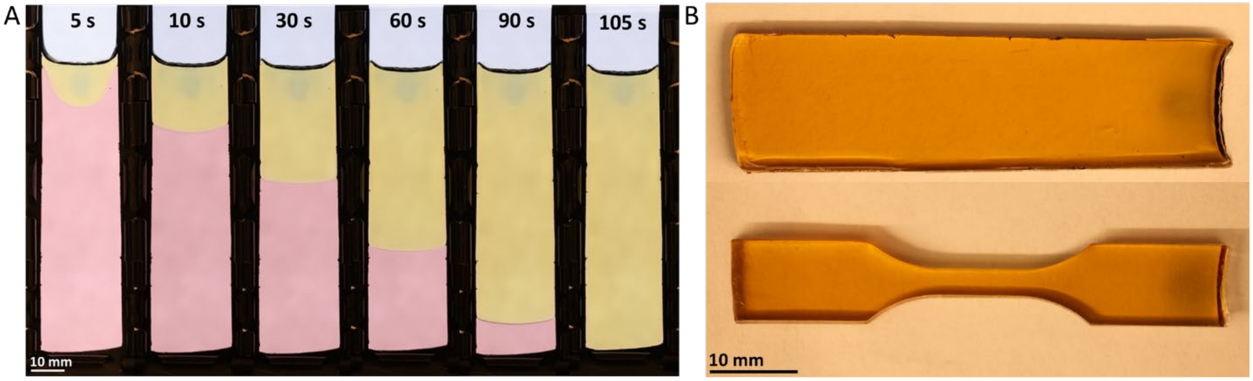

Figure S131: (A) Representative timelapse of 1000:1:1 50 mol% NBE8 in DCPD:G2:TBP. (B) Post-FROMP plaque (top) and subsequent dogbone (bottom) punchout using a Qualitest die (D-638-5-IMP-ASTM). The scale bars are 10 mm.

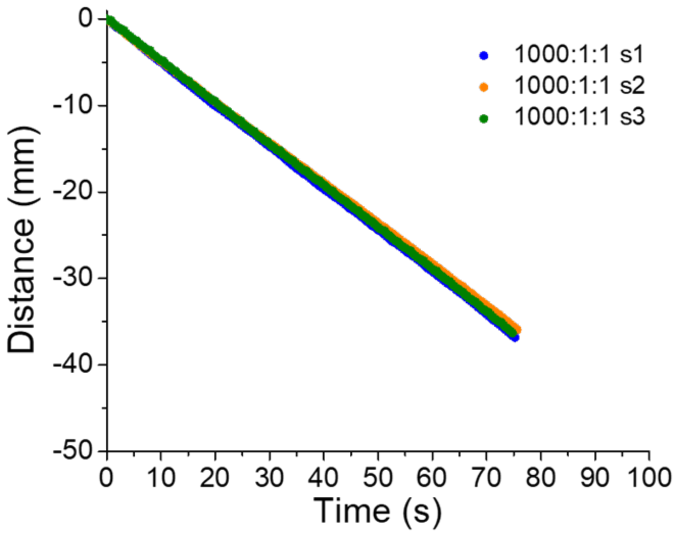

Figure S132: Front tracking of 1000:1:1 1000:1:1 50 mol% NBE8 in DCPD:G2:TBP at 40 °C. (v_f = 0.48 ± 0.01 mm/s).

<u>25 mol% NBE8:</u>

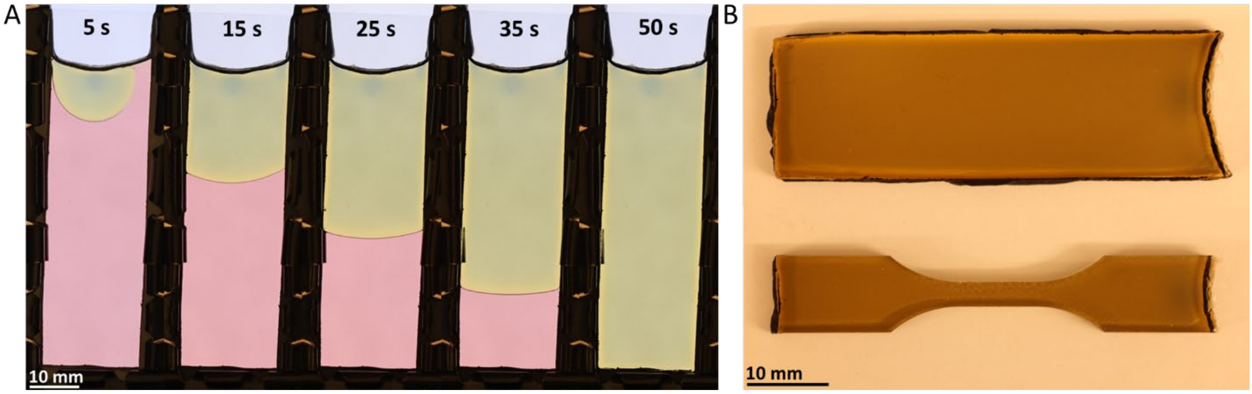

Figure S133: (A) Representative timelapse of 1000:1:1 25 mol% NBE8 in DCPD:G2:TBP. (B) Post-FROMP plaque (top) and subsequent dogbone (bottom) punchout using a Qualitest die (D-638-5-IMP-ASTM). The scale bars are 10 mm.

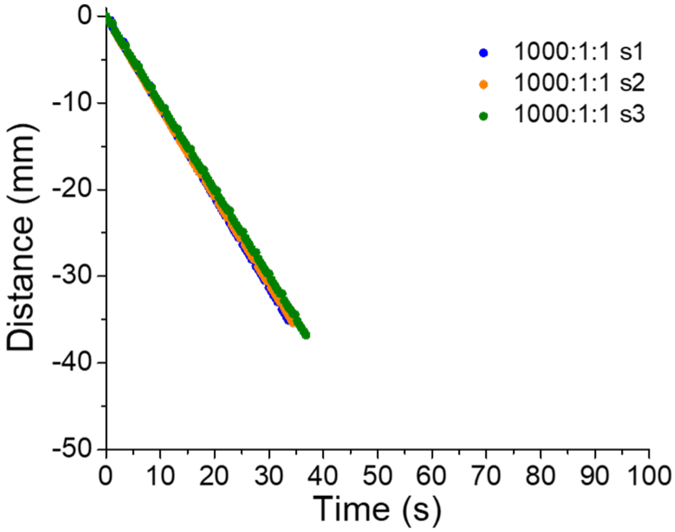

Figure S134: Front tracking of 1000:1:1 1000:1:1 25 mol% NBE8 in DCPD:G2:TBP at 40 °C. (v_f = 1.02 ± 0.02 mm/s).

<u>25 mol% NBE12:</u>

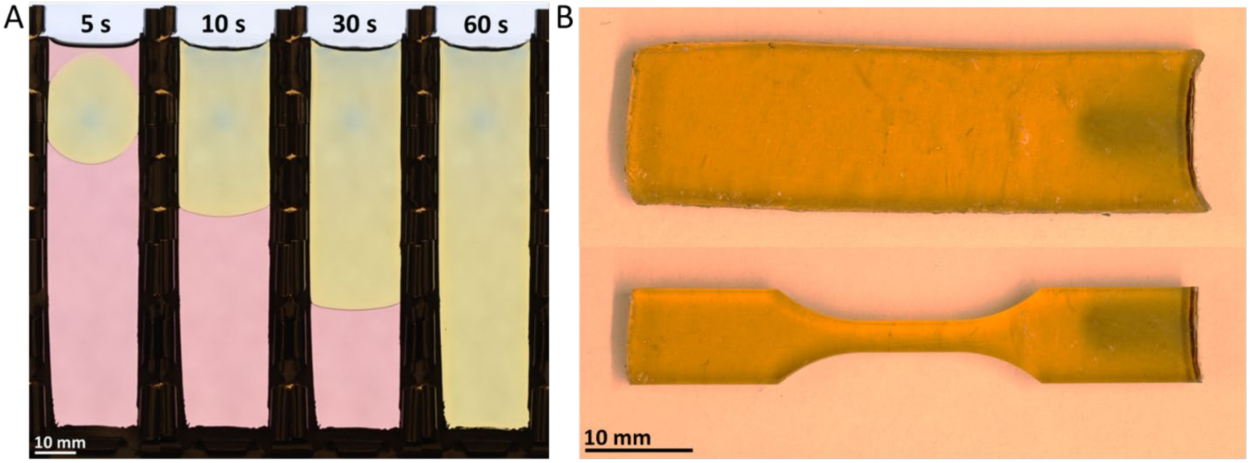

Figure S135: (A) Representative timelapse of 1000:1:1 25 mol% NBE12 in DCPD:G2:TBP. (B) Post-FROMP plaque (top) and subsequent dogbone (bottom) punchout using a Qualitest die (D-638-5-IMP-ASTM). The scale bars are 10 mm.

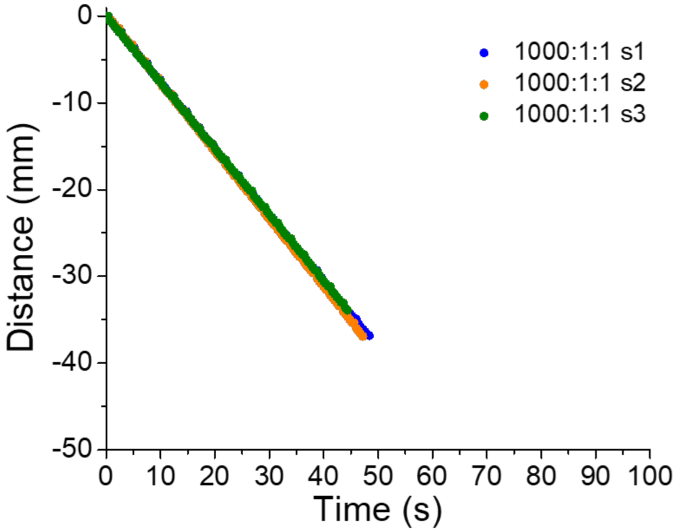

Figure S136: Front tracking of 1000:1:1 1000:1:1 25 mol% NBE12 in DCPD:G2:TBP at 40 °C. (v_f = 0.77 ± 0.01 mm/s).

25 mol% NBE16:

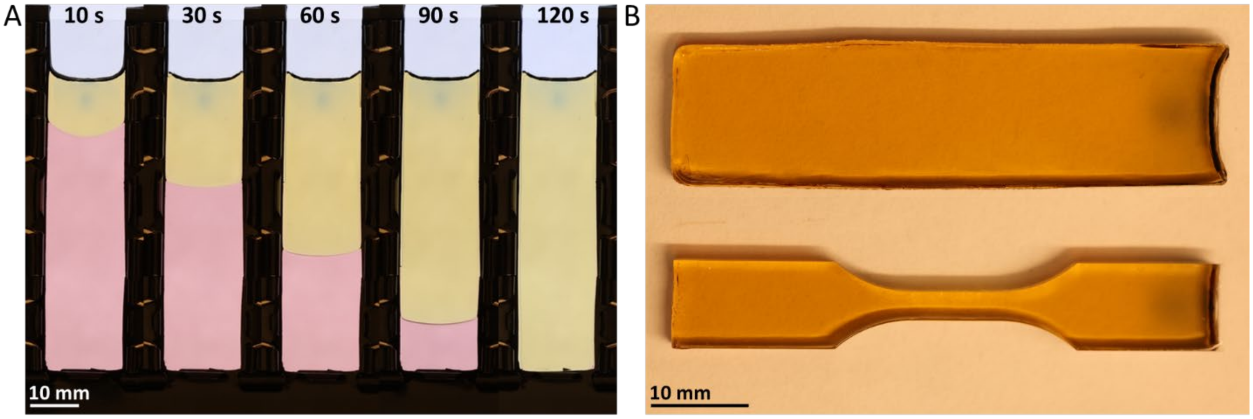

Figure S137: (A) Representative timelapse of 1000:1:1 25 mol% NBE16 in DCPD:G2:TBP. (B) Post-FROMP plaque (top) and subsequent dogbone (bottom) punchout using a Qualitest die (D-638-5-IMP-ASTM). The scale bars are 10 mm.

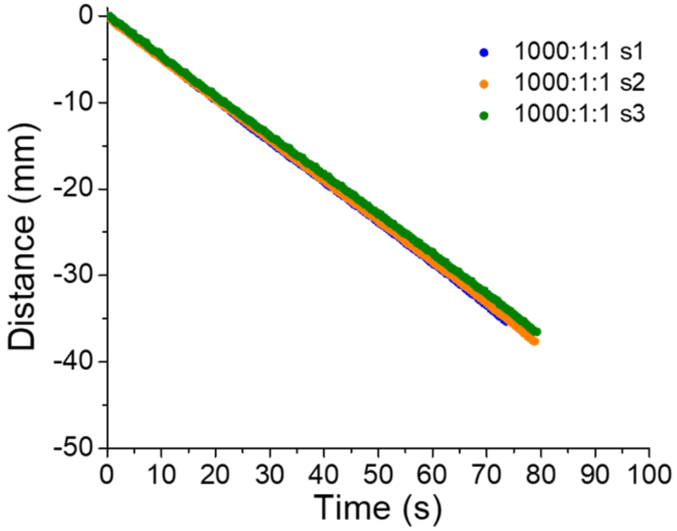

Figure S138: Front tracking of 1000:1:1 1000:1:1 25 mol% NBE16 in DCPD:G2:TBP at 40 °C. (v_f = 0.47 ± 0.01 mm/s).

Table S7: Front velocity for NBE copolymerizations in DCPD at 1000:1:1 loading at 40 °C.

Monomer (equiv)	Initiator (equiv)	Inhibitor (equiv)	Molar incorporation of NBE monomer (%)	Front velocity (mm/s)	error
NBE8					
1000	1	1	50	0.48	0.01
NBE8					
1000	1	1	25	1.02	0.02
NBE12					
1000	1	1	25	0.77	0.01
NBE16					
1000	1	1	25	0.47	0.01

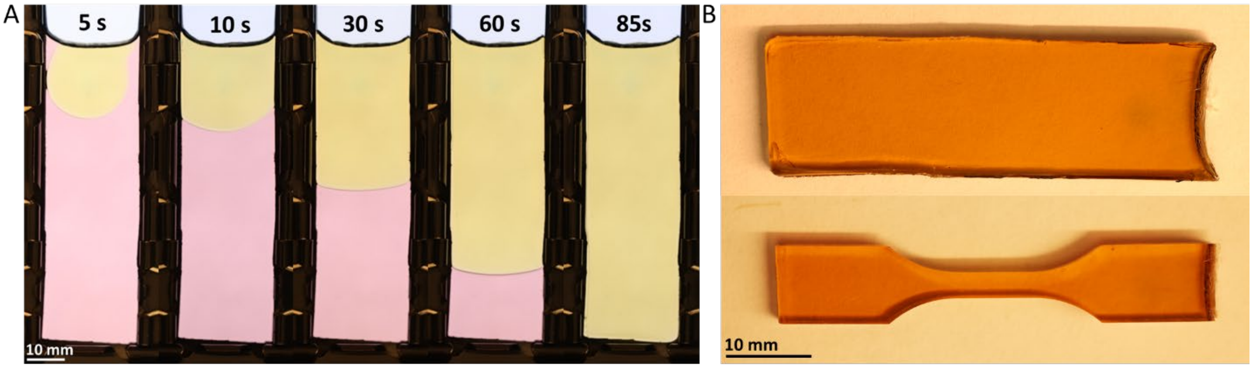

Figure S139: (A) Representative timelapse of 1000:1:1 50 mol% NBE8 in DCPD-H_2:G2:TBP. (B) Post-FROMP plaque (top) and subsequent dogbone (bottom) punchout using a Qualitest die (D-638-5-IMP-ASTM). The scale bars are 10 mm.

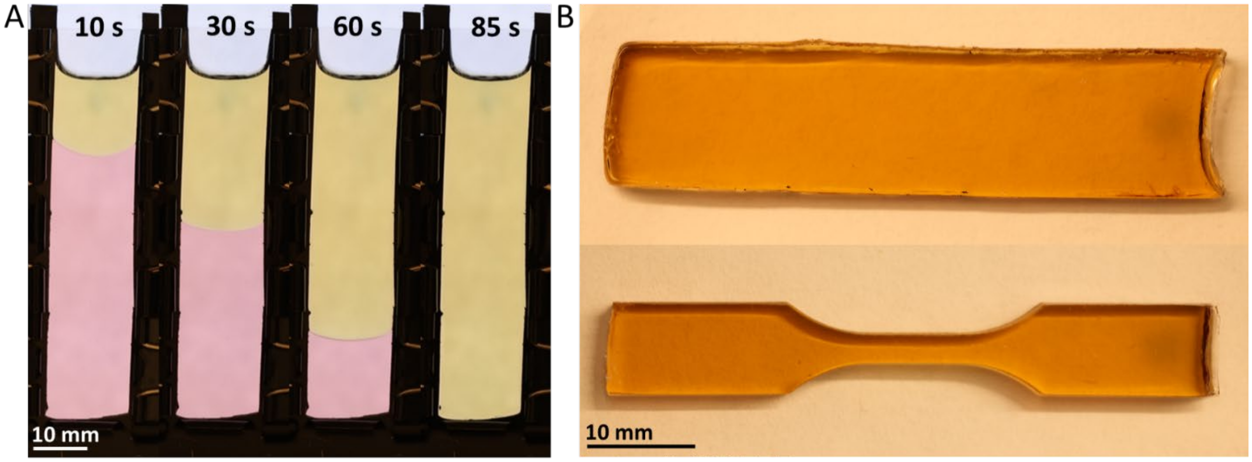

Figure S140: (A) Representative timelapse of 1000:1:1 25 mol% NBE16 in DCPD-H_2:G2:TBP. (B) Post-FROMP plaque (top) and subsequent dogbone (bottom) punchout using a Qualitest die (D-638-5-IMP-ASTM). The scale bars are 10 mm.

DSC Cure Kinetics:

Dynamic Scanning Calorimetry (DSC) Post-FROMP:

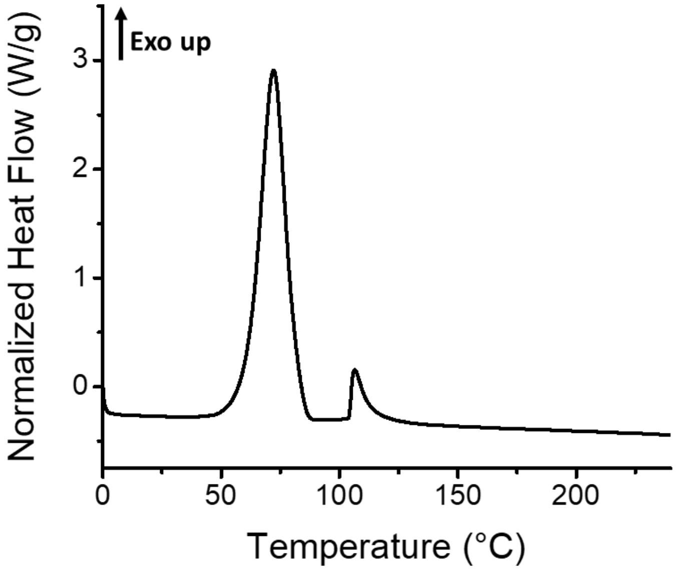

Figure S141: Representative DSC cure kinetic profile of 1000:1:1 50 mol% NBE8 in DCPD:G2:TBP (H_r = 300 ± 1 J/g, Peak Temp = 73 ± 1 °C).

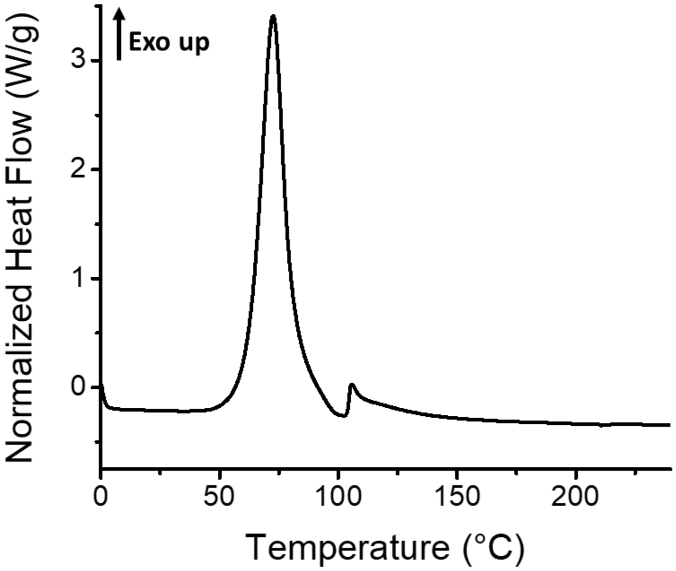

Figure S142: Representative DSC cure kinetic profile of 1000:1:1 25 mol% NBE8 in DCPD:G2:TBP (H_r = 347 ± 1 J/g, Peak Temp = 73 ± 1 °C).

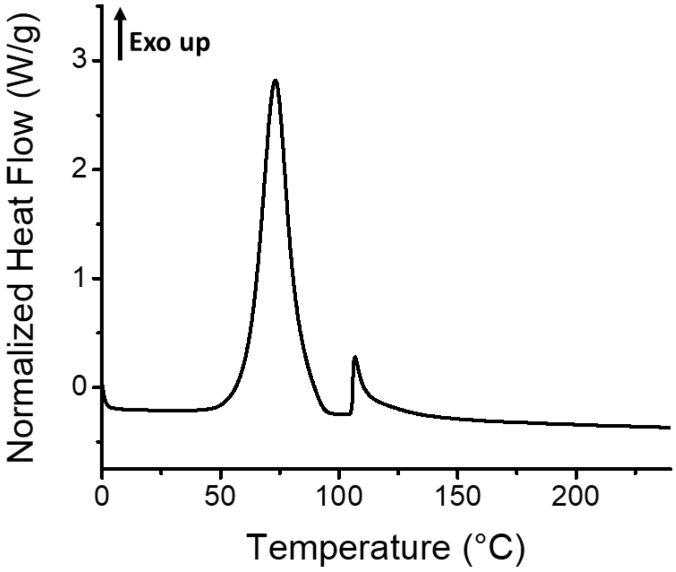

Figure S143: Representative DSC cure kinetic profile of 1000:1:1 25 mol% NBE12 in DCPD:G2:TBP (H_r = 322 ± 6 J/g, Peak Temp = 73 ± 1 °C).

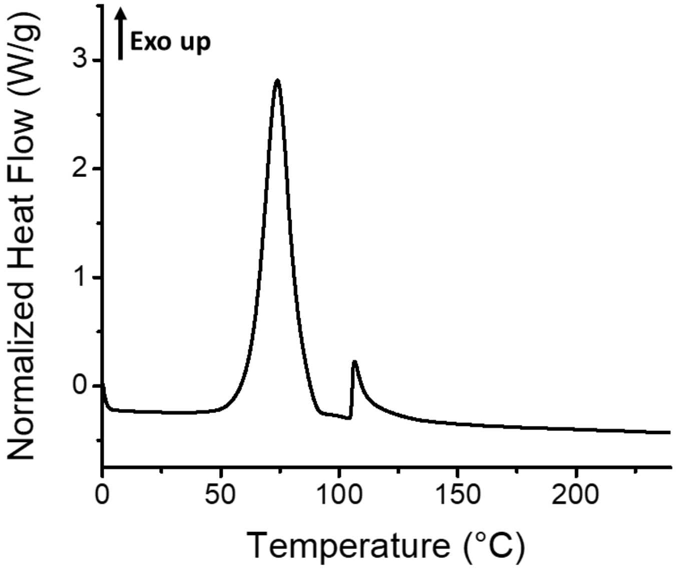

Figure S144: Representative DSC cure kinetic profile of 1000:1:1 25 mol% NBE16 in DCPD:G2:TBP (H_r = 301 ± 8 J/g, Peak Temp = 74 ± 1 °C).

Table S8: Heat of reaction (H_r) and peak temperature (T_{peak}) for DCPD resins at varied loadings.

**Monomer (equiv)**	**Initiator (equiv)**	**Inhibitor (equiv)**	**Molar incorporation of NBE monomer (%)**	**H_r (J/g)**	**error**	**T_{peak} (°C)**	**error**
NBE8							
1000	1	1	50	300	1	72.5	0.5
NBE8							
1000	1	1	25	347	1	72.5	0.1
NBE12							
1000	1	1	25	322	6	73.0	0.3
NBE16							
1000	1	1	25	301	8	73.9	0.1

Dynamic Scanning Calorimetry (DSC) Post-FROMP:

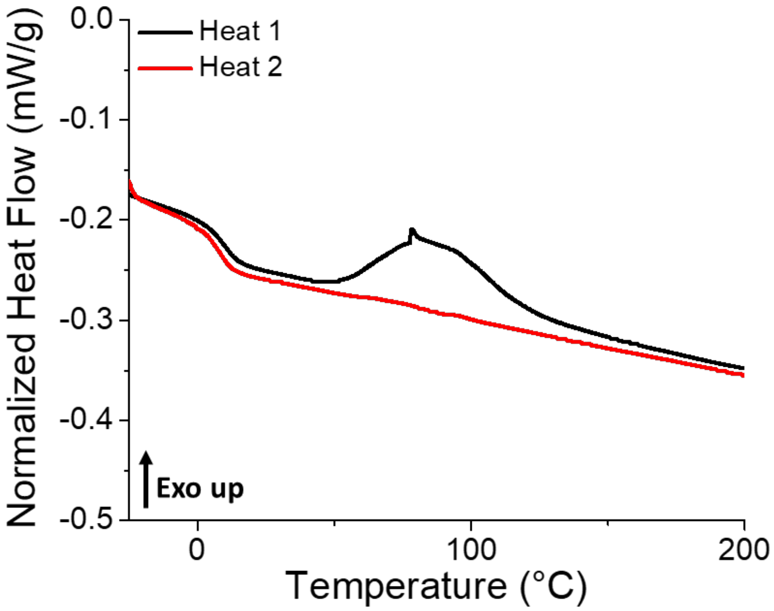

Figure S145: Representative DSC of 1000:1:1 50 mol% NBE8 in DCPD:G2:TBP post-FROMP (T_g = 8.3 ± 0.3 °C, $H_{r,\ residual}$ = 22 ± 4 J/g). First (black) and second heat (red) cycle. Reported T_g is taken from the second heat cycle.

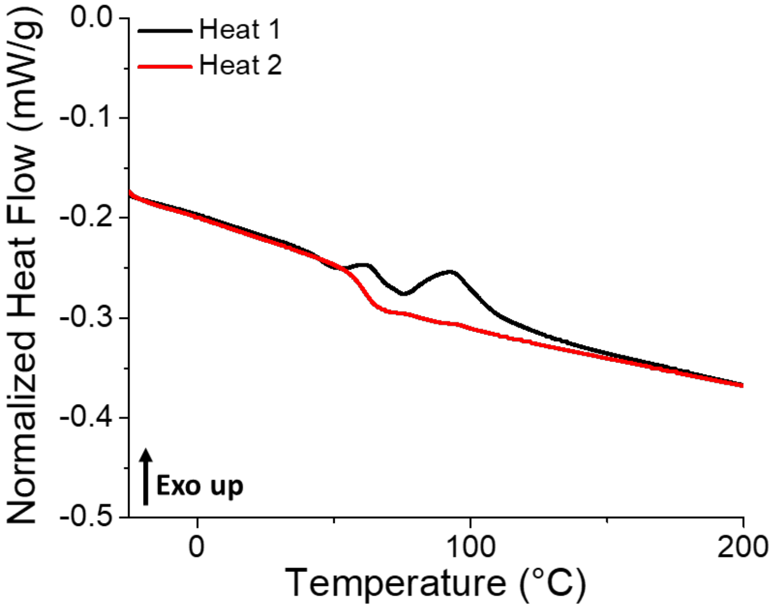

Figure S146: Representative DSC of 1000:1:1 25 mol% NBE8 in DCPD:G2:TBP post-FROMP (T_g = 61 ± 1 °C, $H_{r,\ residual}$ = 5.5 ± 0.7 J/g). First (black) and second heat (red) cycle. Reported T_g is taken from the second heat cycle.

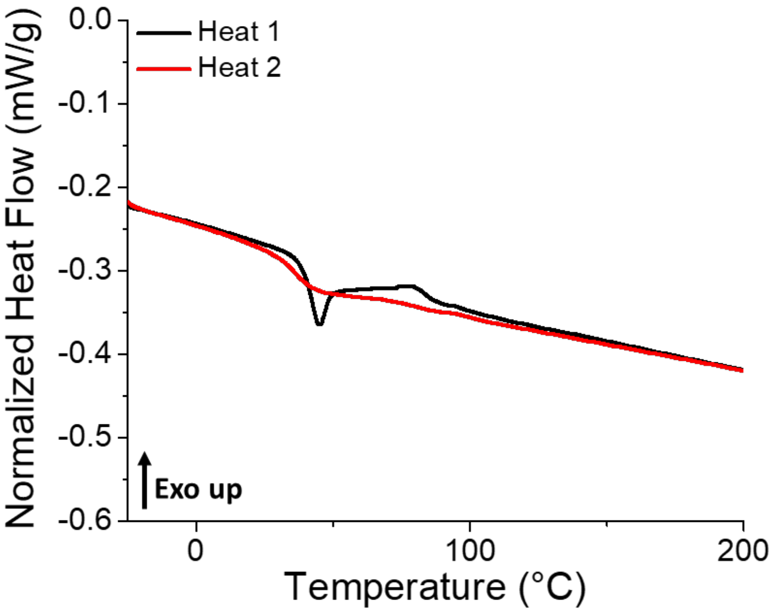

Figure S147: Representative DSC of 1000:1:1 25 mol% NBE12 in DCPD:G2:TBP post-FROMP (T_g = 37 ± 1 °C, $H_{r,\,residual}$ = 9.0 ± 3.3 J/g). First (black) and second heat (red) cycle. Reported T_g is taken from the second heat cycle.

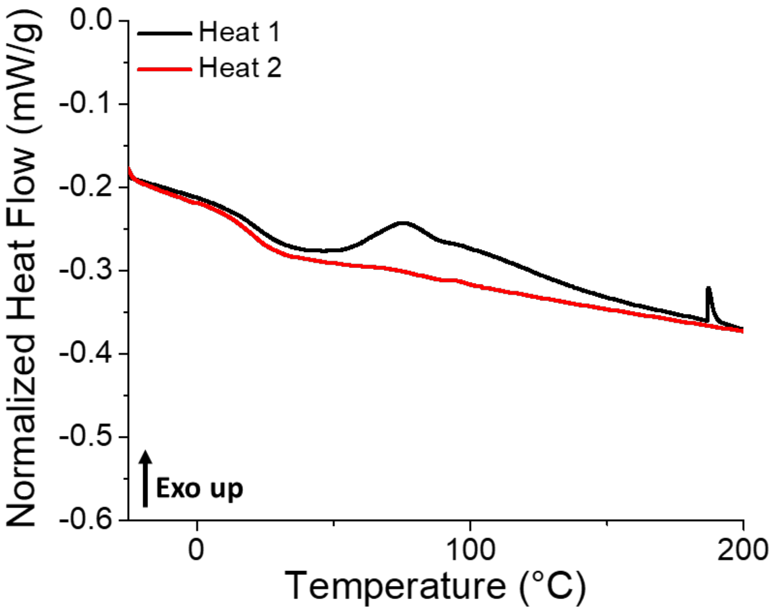

Figure S148: Representative DSC of 1000:1:1 25 mol% NBE16 in DCPD:G2:TBP post-FROMP (T_g = 21 ± 1 °C, $H_{r,\,residual}$ = 9.3 ± 3.8 J/g). First (black) and second heat (red) cycle. Reported T_g is taken from the second heat cycle.

Table S9: Glass transition temperature (T_g) for pNBE-*co*-DCPD-H_2 post-FROMP at varied loadings. T_g is taken from the second heat cycle.

Monomer (equiv)	Initiator (equiv)	Inhibitor (equiv)	Molar incorporation of NBE monomer (%)	T_g (°C)	error
NBE8					
1000	1	1	50	8.3	0.3
NBE8					
1000	1	1	25	61	1
NBE12					
1000	1	1	25	37	1
NBE16					
1000	1	1	25	21	1

Table S10: Residual heat of reaction ($H_{r,\ residual}$) and percent monomer conversion for pDCPD-H_2 post-FROMP at varied loadings.

Monomer (equiv)	Initiator (equiv)	Inhibitor (equiv)	Molar incorporation of NBE monomer (%)	$H_{r,\ residual}$ (J/g)	error	Calculated Conversion (%)	error
NBE8							
1000	1	1	50	22	4	93	1
NBE8							
1000	1	1	25	6	1	98	1
NBE12							
1000	1	1	25	9	3	97	1
NBE16							
1000	1	1	25	9	4	97	1

Dynamic Mechanical Analysis (DMA):

DMA samples were prepared in a U-type mold (3 mm thickness) at 40 °C initial temperature as shown in literature.[1]

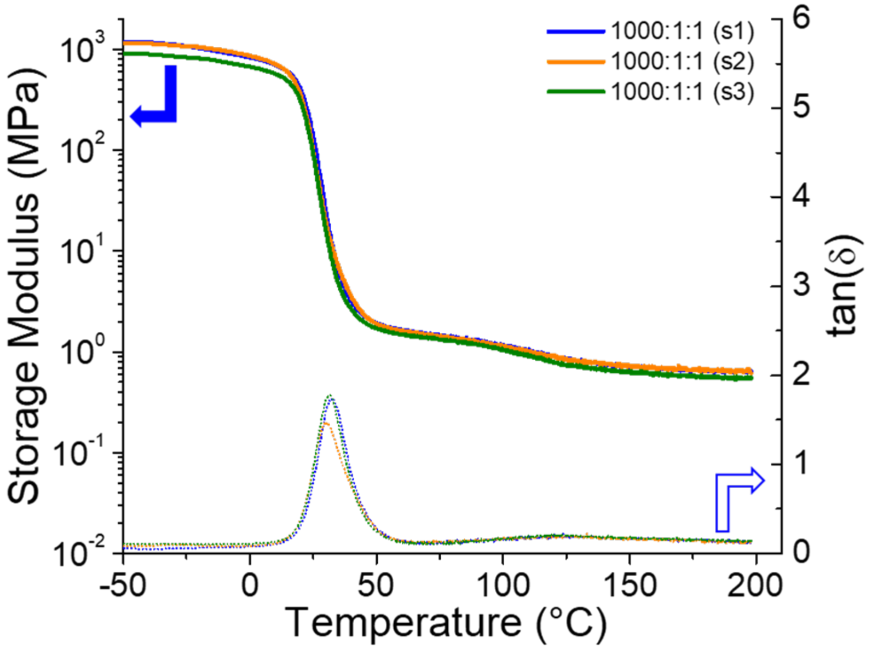

Figure S149: Triplicate DMA temperature ramp of 1000:1:1 50 mol% NBE8 in DCPD:G2:TBP post-FROMP.

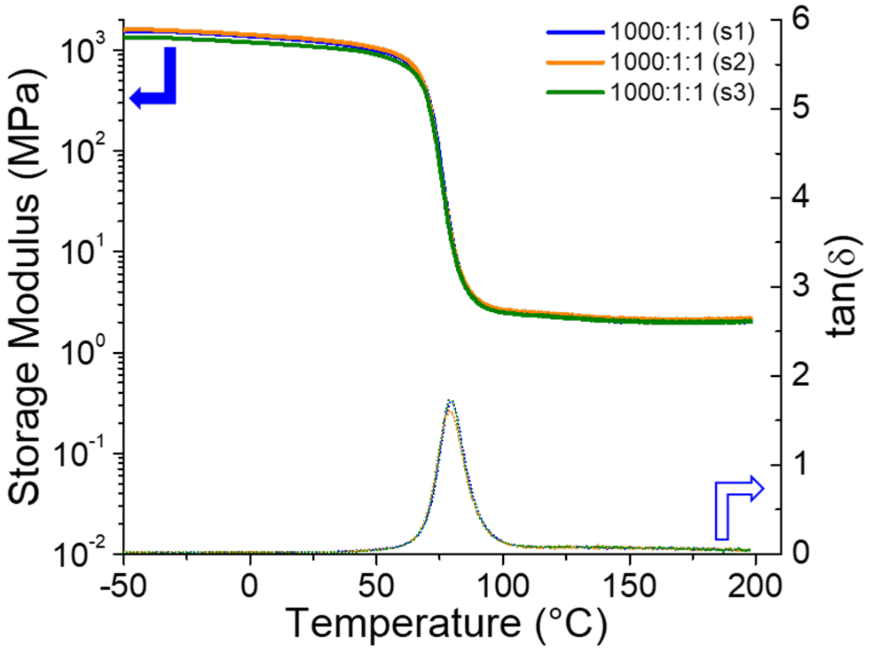

Figure S150: Triplicate DMA temperature ramp of 1000:1:1 25 mol% NBE8 in DCPD:G2:TBP post-FROMP.

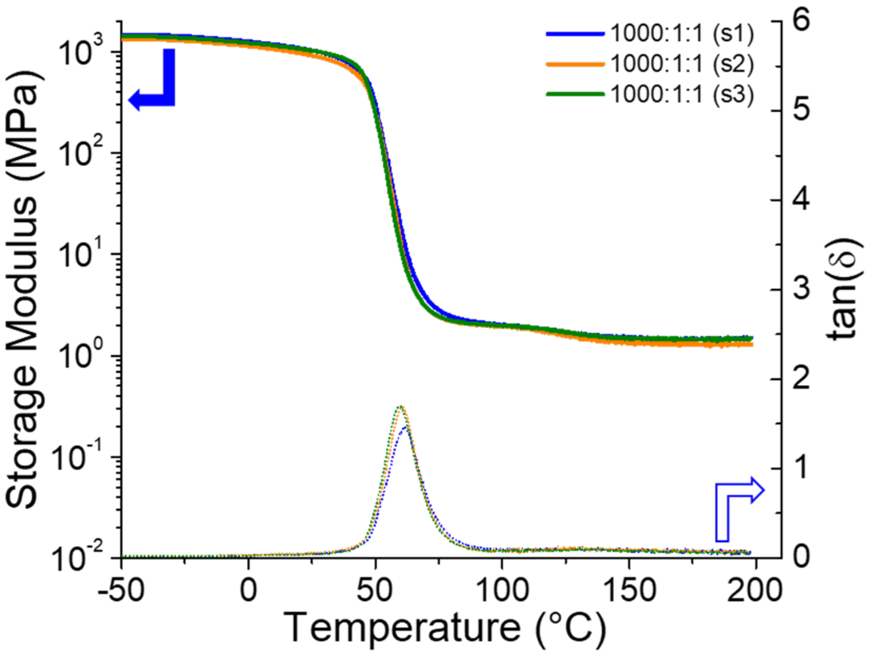

Figure S151: Triplicate DMA temperature ramp of 1000:1:1 25 mol% NBE12 in DCPD:G2:TBP post-FROMP.

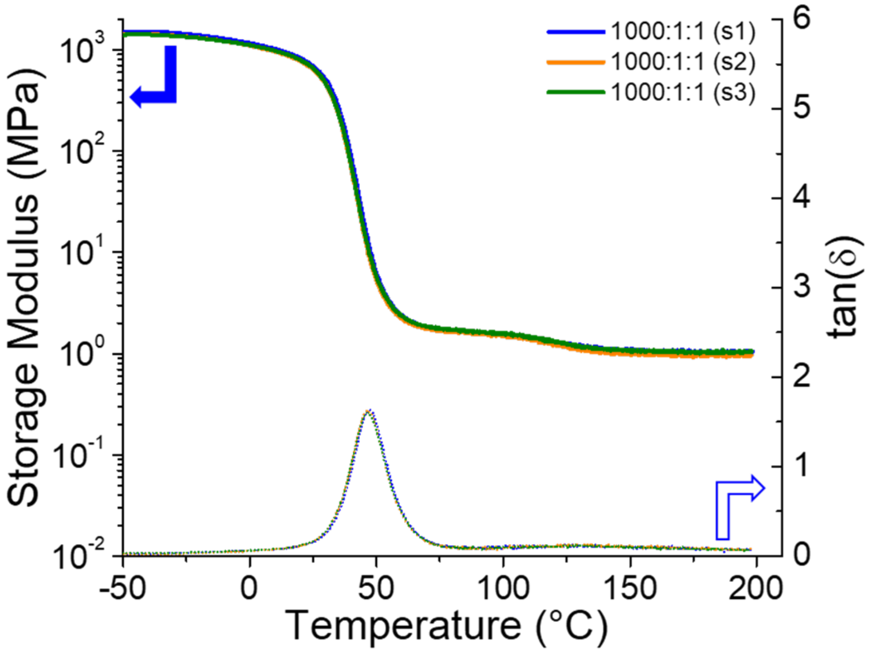

Figure S152: Triplicate DMA temperature ramp of 1000:1:1 25 mol% NBE16 in DCPD:G2:TBP post-FROMP.

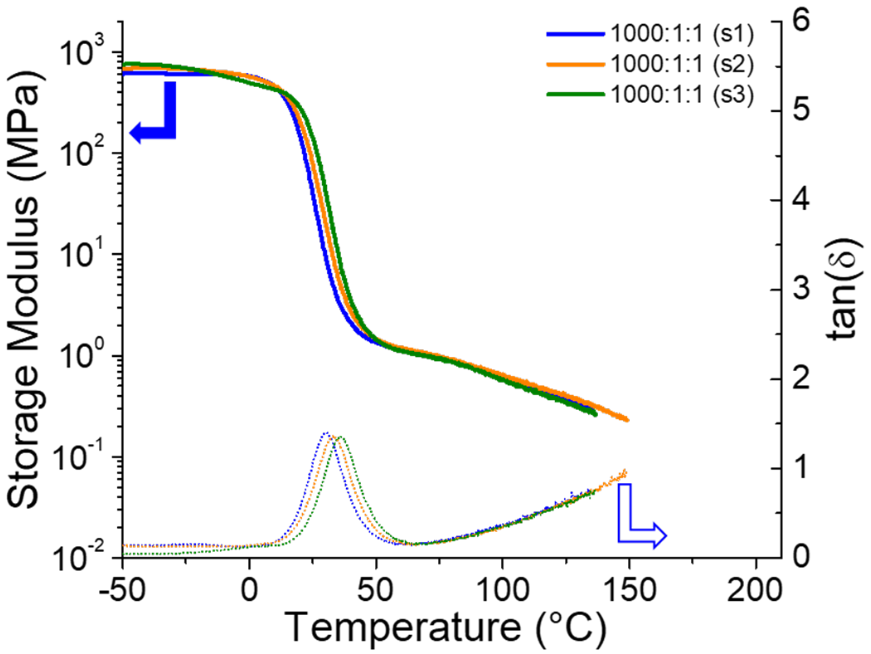

Figure S153: Triplicate DMA temperature ramp of 1000:1:1 50 mol% NBE8 in DCPD-H_2:G2:TBP post-FROMP.

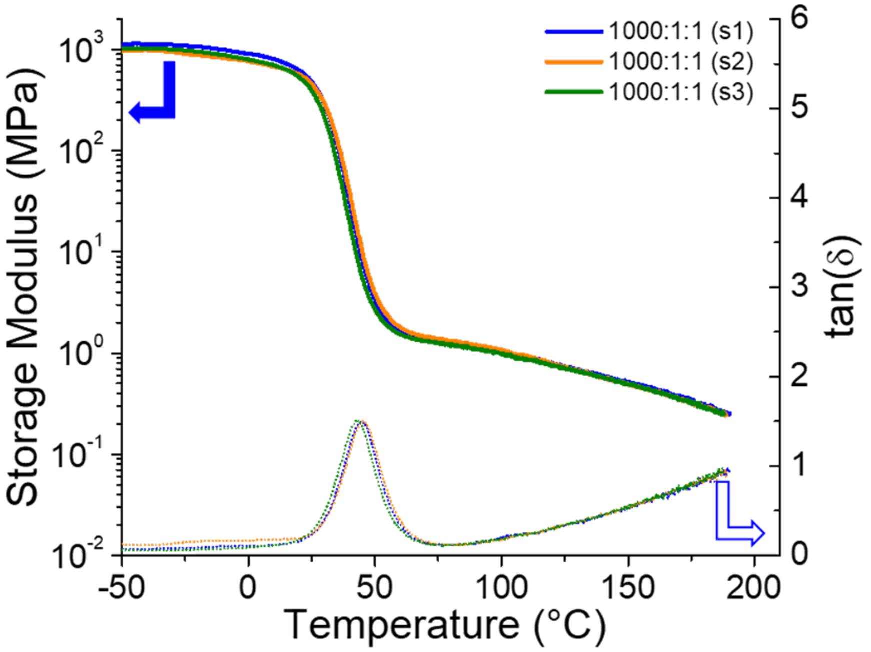

Figure S154: Triplicate DMA temperature ramp of 1000:1:1 25 mol% NBE16 in DCPD-H_2:G2:TBP post-FROMP.

Table S11: Glass transition temperature (T_g), glassy modulus (storage modulus below T_g), and M_x determination (n = 3) for variable loading of NBE monomer copolymerized with DCPD post-FROMP at 1000:1:1 monomer:initiator:inhibitor.

Sample	Loading	$T_{g, DMA}$ (°C)				
		S1	S2	S3	**average**	**error**
50 mol% NBE8	1000:1:1	30.1	31.5	30.5	**30.7**	**0.6**
25 mol% NBE8	1000:1:1	84.3	79.0	80.0	**81.1**	**2.3**
25 mol% NBE12	1000:1:1	61.4	60.2	59.9	**60.5**	**0.6**
25 mol% NBE16	1000:1:1	47.1	46.8	46.7	**46.9**	**0.2**
Sample	Loading	**Storage Modulus (*E*', MPa) at 25 °C**				
		S1	S2	S3	**average**	**error**
50 mol% NBE8	1000:1:1	99.9	90.4	70.4	**86.9**	**12.3**
25 mol% NBE8	1000:1:1	1230	1281	1086	**1199**	**83**
25 mol% NBE12	1000:1:1	1033	919.2	1028	**993.4**	**53**
25 mol% NBE16	1000:1:1	687.5	640.5	651.0	**660.0**	**20.1**
Sample	Loading	**Storage Modulus (*E*', MPa) at 180 °C**				
		S1	S2	S3	**average**	**error**
50 mol% NBE8	1000:1:1	0.666	0.661	0.571	**0.633**	**0.044**
25 mol% NBE8	1000:1:1	2.003	2.134	2.030	**2.056**	**0.056**
25 mol% NBE12	1000:1:1	1.461	1.291	1.470	**1.407**	**0.082**
25 mol% NBE16	1000:1:1	1.027	0.959	1.034	**1.007**	**0.034**
Sample	Loading	**M_x (kg/mol) (assuming ν = 0.4/0.5)**				
		S1	S2	S3	**average**	**error**
50 mol% NBE8	1000:1:1	16.2/17.4	16.3/17.5	18.9/20.2	**17.1/18.4**	**1.2/1.3**
25 mol% NBE8	1000:1:1	5.45/5.84	5.12/5.48	5.38/5.76	**5.31/5.69**	**0.14/0.15**
25 mol% NBE12	1000:1:1	7.38/7.91	8.35/8.95	7.33/7.86	**7.69/8.24**	**0.47/0.50**
25 mol% NBE16	1000:1:1	10.4/11.1	11.1/11.9	10.3/11.0	**10.6/11.4**	**0.4/0.4**

Density Measurements (Archimedes Kit):

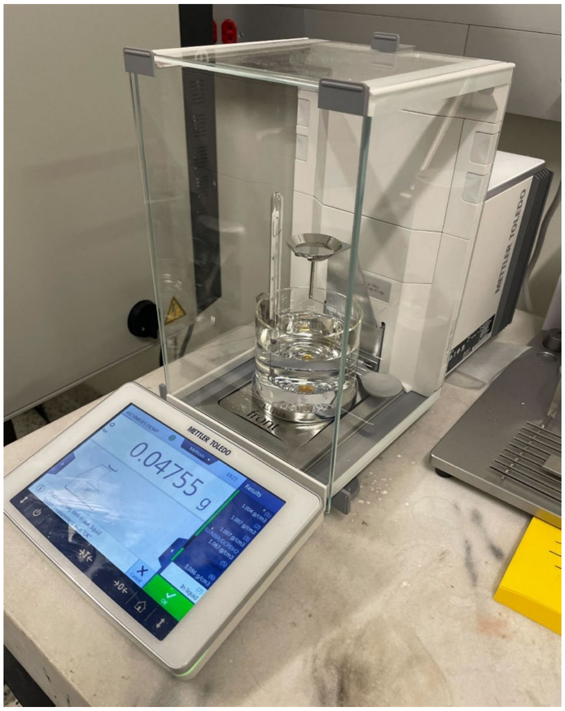

Figure S155: Setup for density calculation for polymer materials using an Archimedes kit.

Table S12: Density determination (n = 3) for NBE copolymer networks post-FROMP at 1000:1:1 monomer:initiator:inhibitor.

Sample	Loading	Density (g/mL)				
		S1	S2	S3	**average**	**error**
50 mol% NBE8	1000:1:1	1.022	1.022	1.021	**1.022**	**0.001**
25 mol% NBE8	1000:1:1	1.035	1.035	1.035	**1.035**	**0.000**
25 mol% NBE12	1000:1:1	1.022	1.022	1.023	**1.022**	**0.001**
25 mol% NBE16	1000:1:1	1.009	1.009	1.012	**1.010**	**0.001**

The molecular weight between crosslinks (M_x) was calculated using the tensile storage modulus (E') from the rubbery plateau.

$$G' = (E')/(2(1+\nu)) = \rho RT/M_x$$ [Equation S1][4, 5]

$$M_x = (2.8(\rho RT))/(E')$$

Using the average densities of the polymeric materials, Poisson's ratio of pDCPD (ν = 0.4),[6-8] the ideal gas constant (R = 8.314 J/mol*K)), and the storage modulus at 180 °C, M_x was calculated using Equation S1. **It is important to note that ν = 0.4 was assumed for our samples, as it is often used for pDCPD networks, but we have also added ν = 0.5 (used for elastomeric cyclooctadiene-co-DCPD FROMP-dervied networks[9]) for all samples as this value was not determined experimentally.**

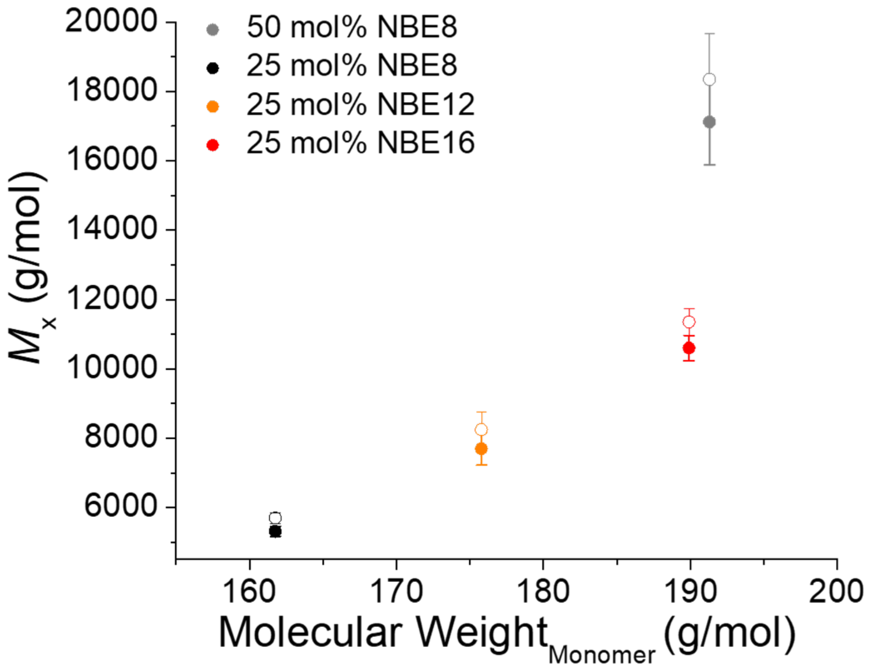

Figure S156: Scatter plot of average monomer molecular weight versus calculated molecular weight between crosslinks (M_x) for norbornene ester copolymer networks at 1000:1:1 NBE in DCPD:G2:TBP assuming v = 0.4 (closed dots) or 0.5 (open dots).

Swelling Studies:

Table S13: Swelling ratios (n = 3) for NBE copolymer networks post-FROMP at 1000:1:1 monomer:initiator:inhibitor.

Sample	Loading	Swelling Ratio (%)				
		s1	s2	s3	**average**	**error**
50 mol% NBE8	1000:1:1	750.3	897.2	821.5	**823.0**	**60.0**
25 mol% NBE8	1000:1:1	369.3	388.5	382.6	**380.1**	**8.0**
25 mol% NBE12	1000:1:1	445.0	468.8	426.5	**446.8**	**17.3**
25 mol% NBE16	1000:1:1	505.5	540.2	501.8	**515.8**	**17.3**

Tensile Testing:

Timelapses and stress-strain curves of specimen elongation until failure

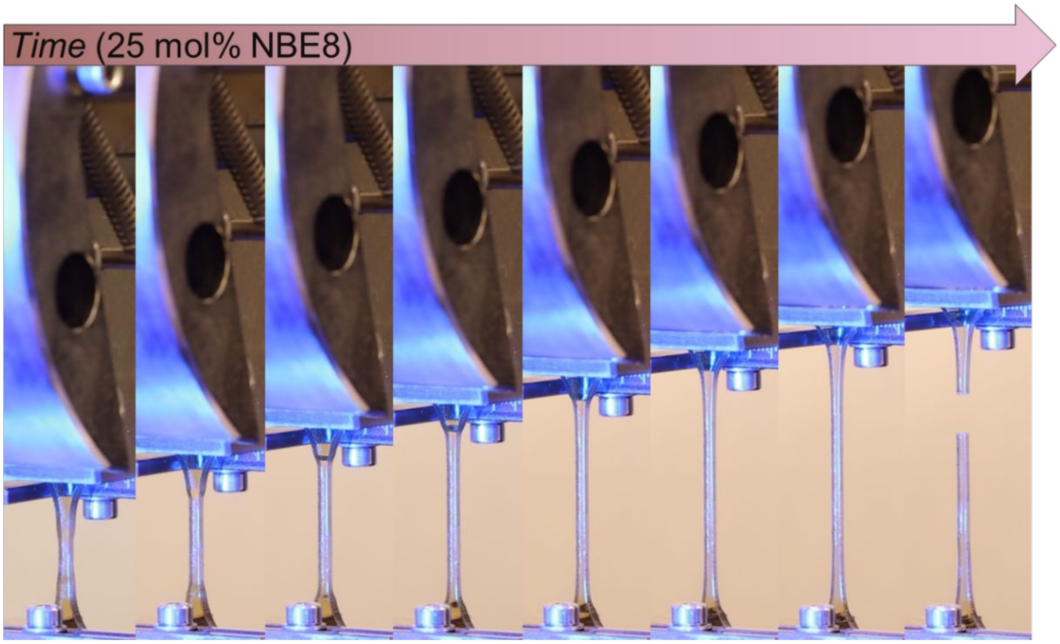

Figure S157: Representative timelapse of 1000:1:1 25 mol% NBE8 in DCPD:G2:TBP.

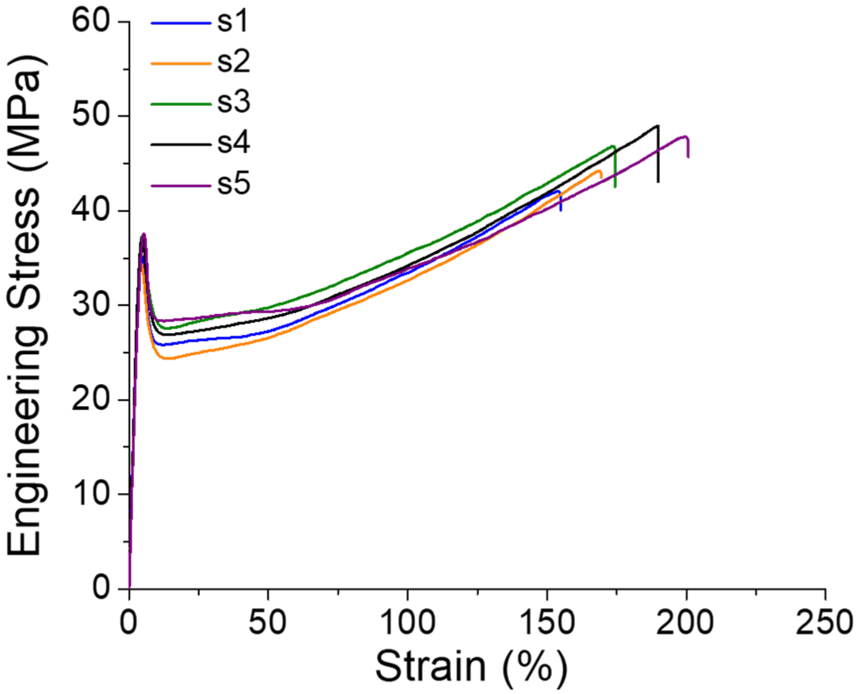

Figure S158: Stress-strain curves of 1000:1:1 25 mol% NBE8 in DCPD:G2:TBP (n = 5).

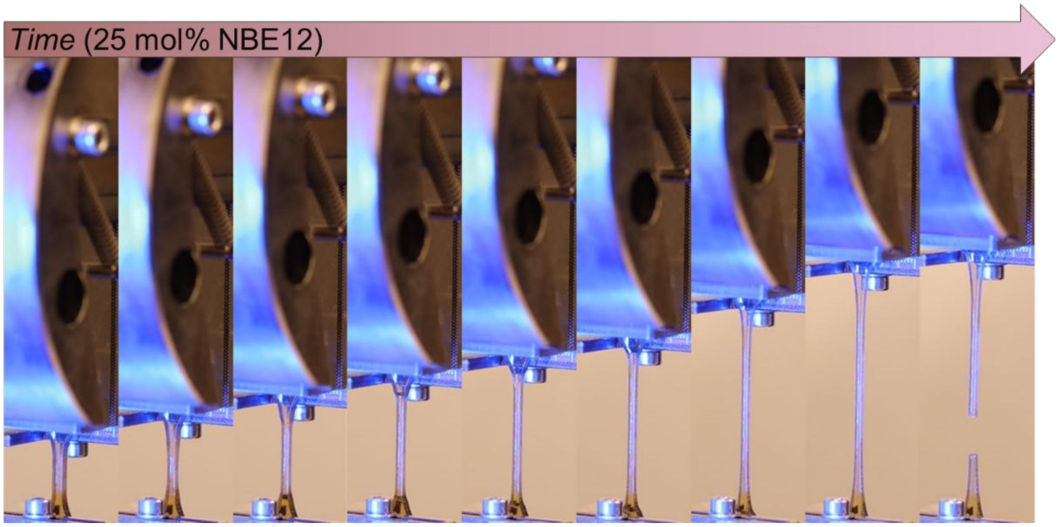

Figure S159: Representative timelapse of 1000:1:1 25 mol% NBE12 in DCPD:G2:TBP.

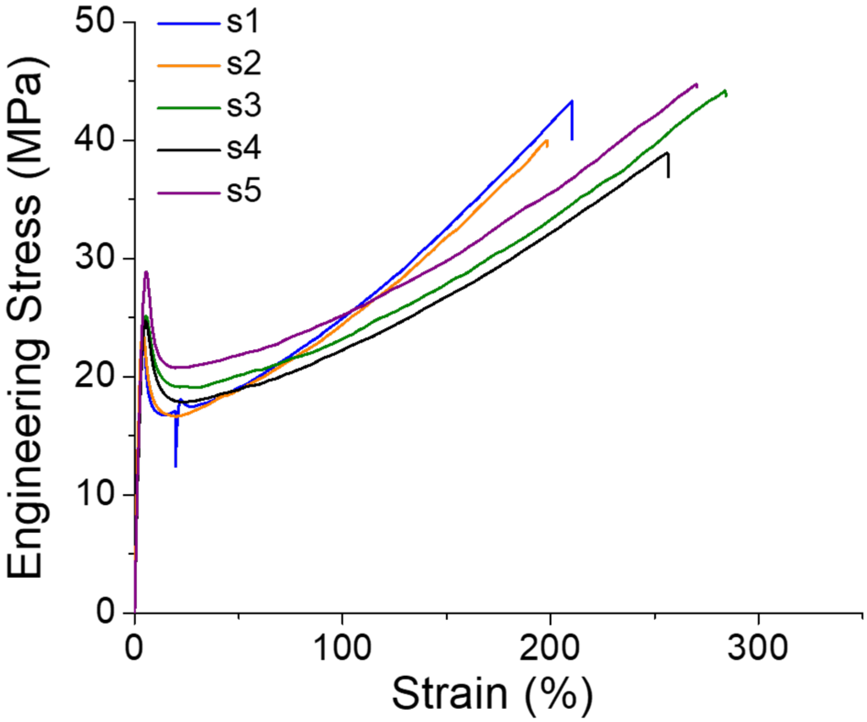

Figure S160: Stress-strain curves of 1000:1:1 25 mol% NBE12 in DCPD:G2:TBP (n = 5).

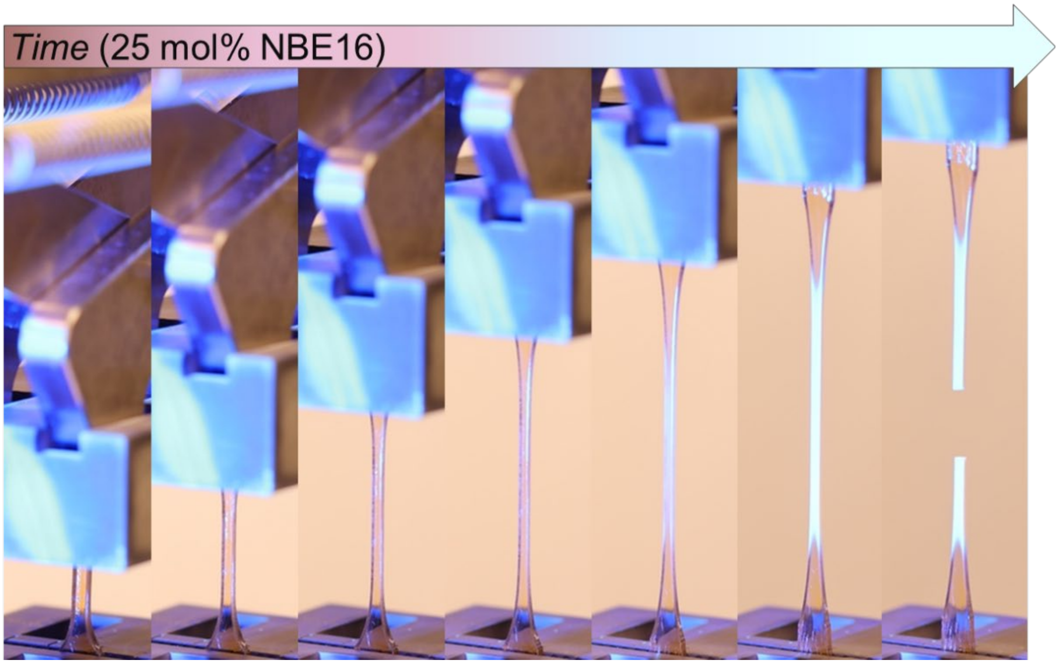

Figure S161: Representative timelapse of 1000:1:1 25 mol% NBE16 in DCPD:G2:TBP.

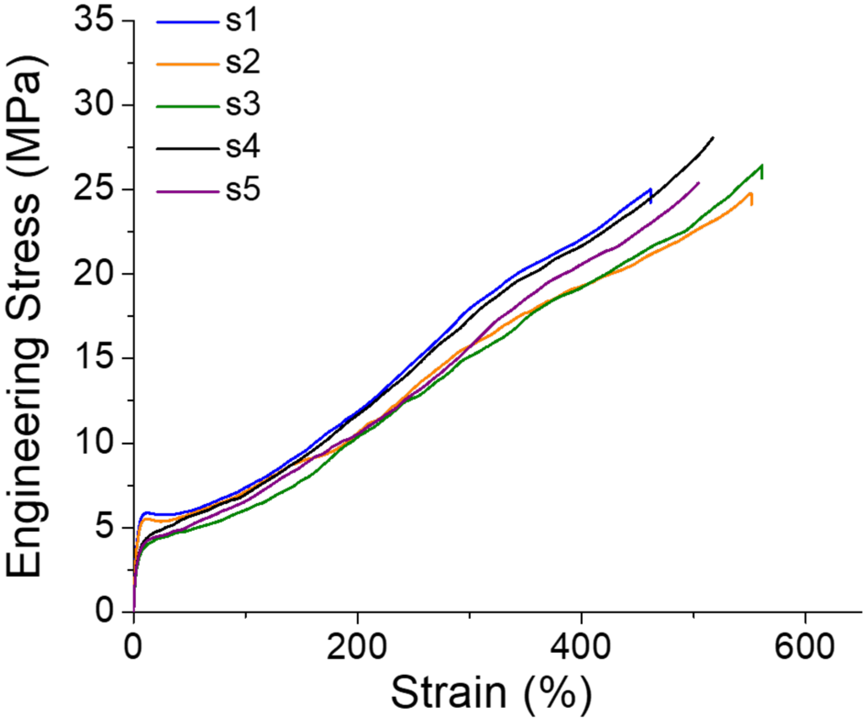

Figure S162: Stress-strain curves of 1000:1:1 25 mol% NBE16 in DCPD:G2:TBP (n = 5).

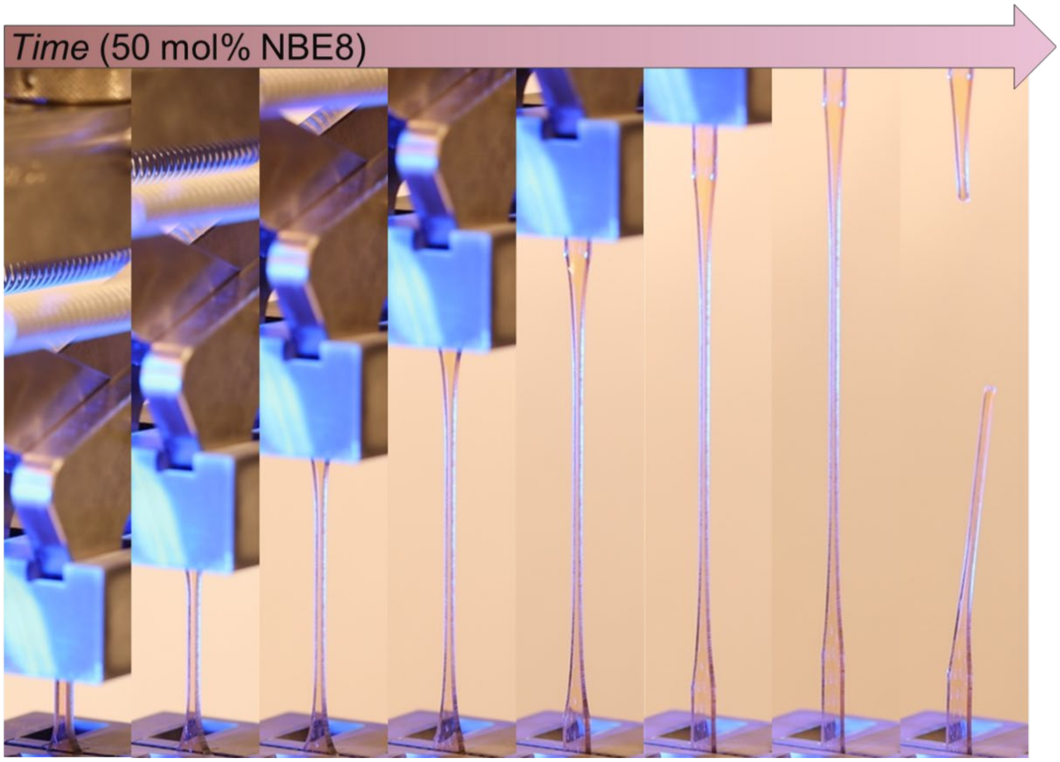

Figure S163: Representative timelapse of 1000:1:1 50 mol% NBE8 in DCPD:G2:TBP.

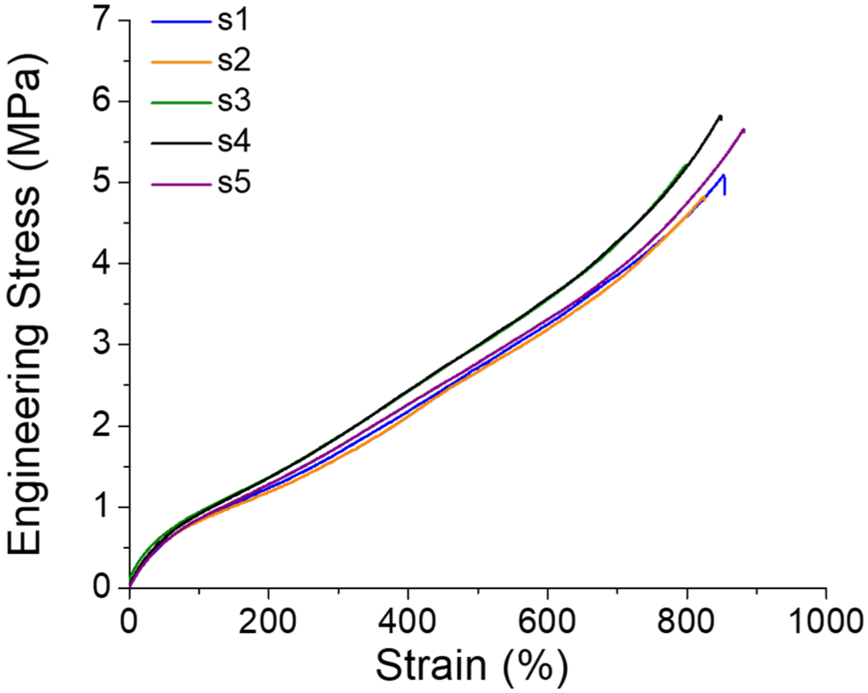

Figure S164: Stress-strain curves of 1000:1:1 50 mol% NBE8 in DCPD:G2:TBP (n = 5).

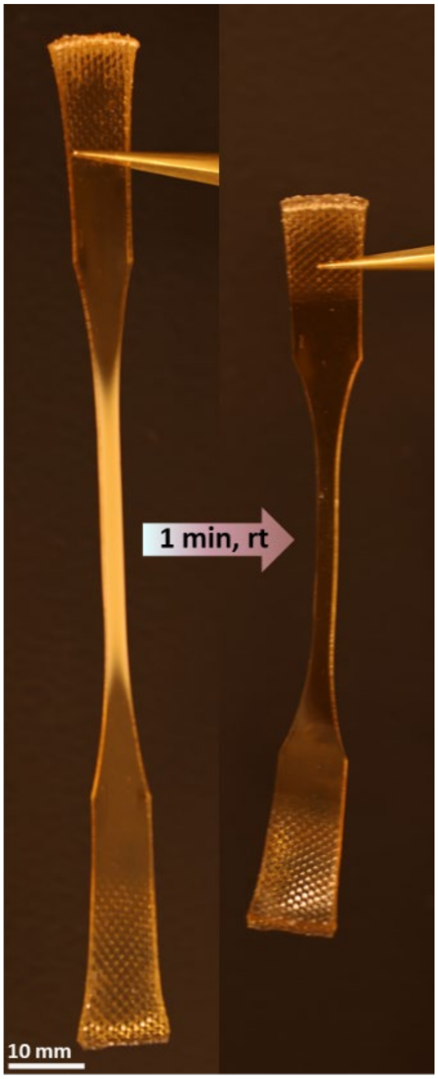

Figure S165: Rapid reversibility of strain-whitening in elongated 1000:1:1 25 mol% NBE16 in DCPD:G2:TBP at ambient temperatures.

Hysteresis Testing:

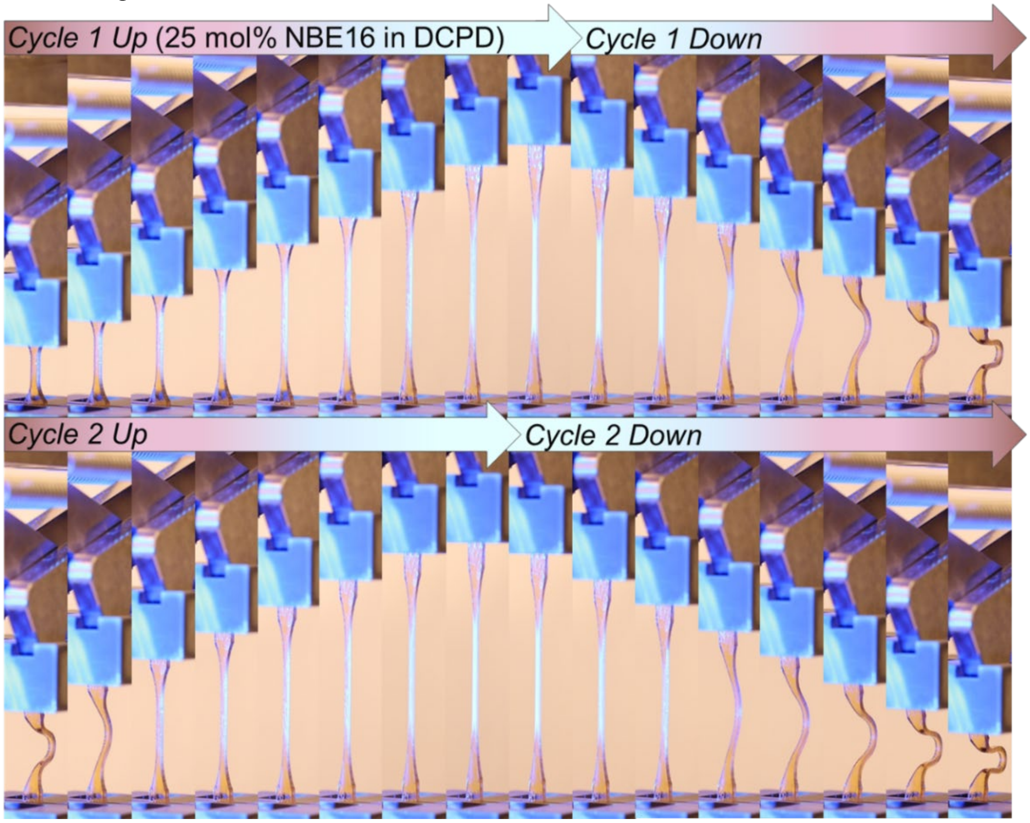

Figure S166: Hysteresis timelapse 25 mol% NBE16 (Cycle 1 and 2) showing reversibility of strain-whitening. After 2-3 cycles the material was increasingly more permanently deformed and no longer demonstrated strain-whitening until Cycle 7 to failure (Figures S168-170).

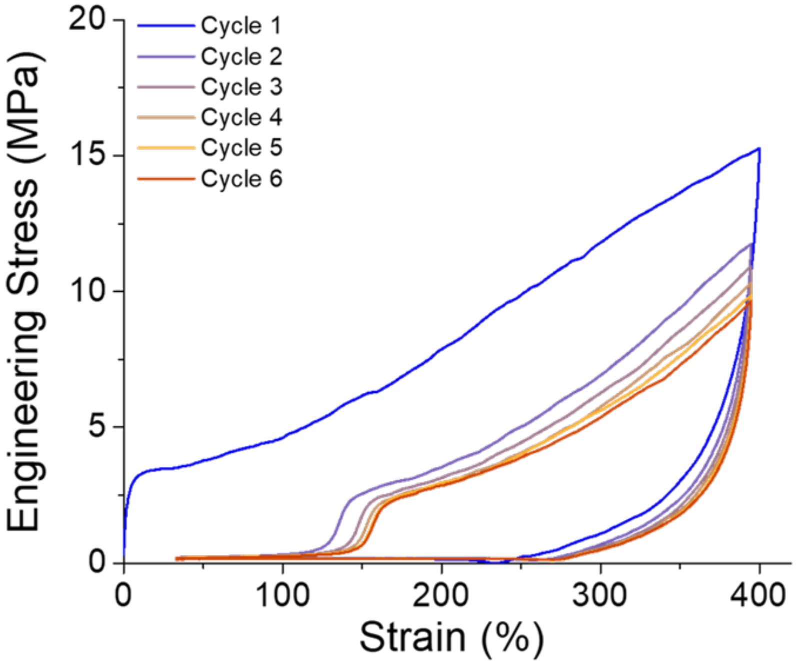

Figure S167: Representative hysteresis stress-strain curves 25 mol% NBE16.

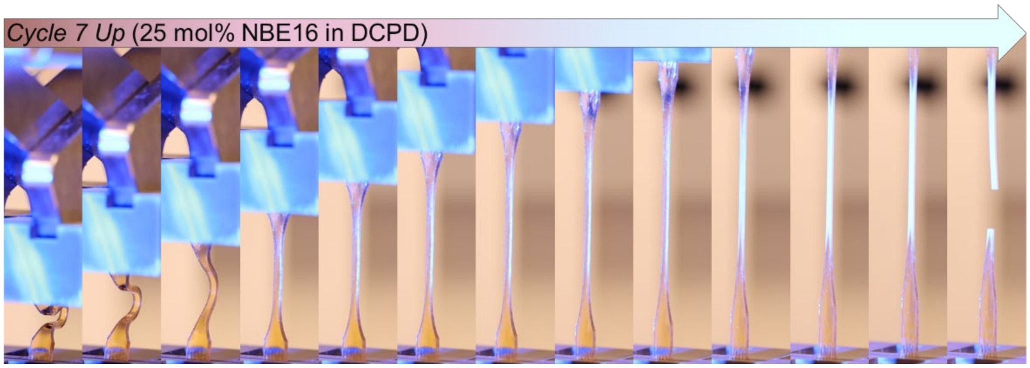

Figure S168: Timelapse of 25 mol% NBE16 hysteresis Cycle 7 which was programmed to pull until failure.

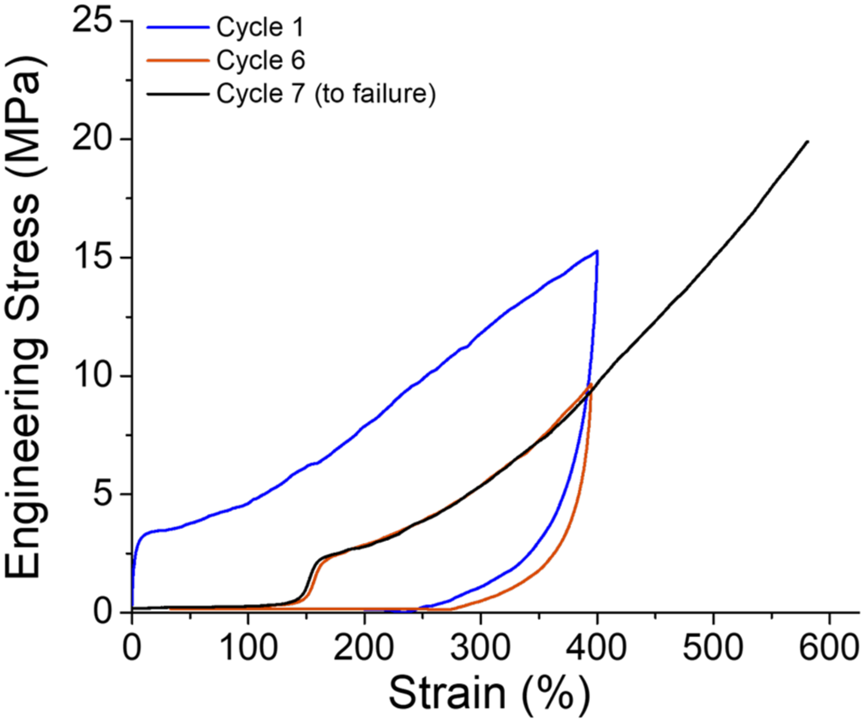

Figure S169: Representative hysteresis stress-strain curve of 25 mol% NBE16 hysteresis Cycle 7 (until failure) overlayed with Cycle 1 and 6.

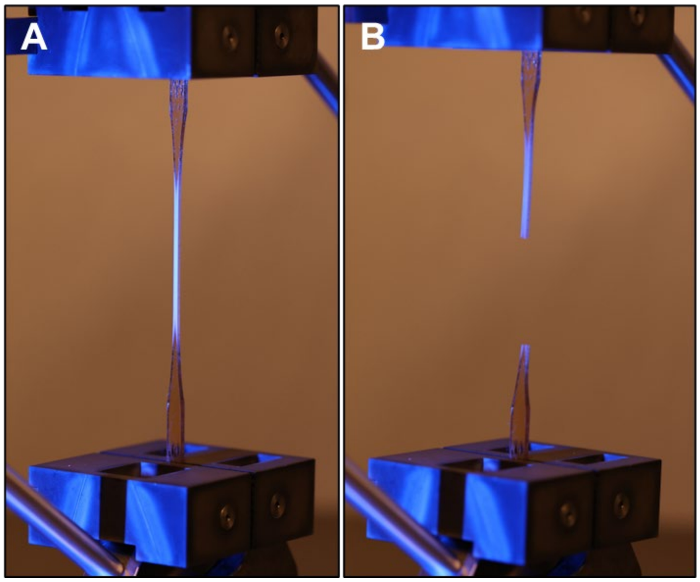

Figure S170: Representative image of 25 mol% NBE16 hysteresis Cycle 7 demonstrating strain-whitening before failure (A) and post-failure (B).

Tensile Testing with Increased Rate:

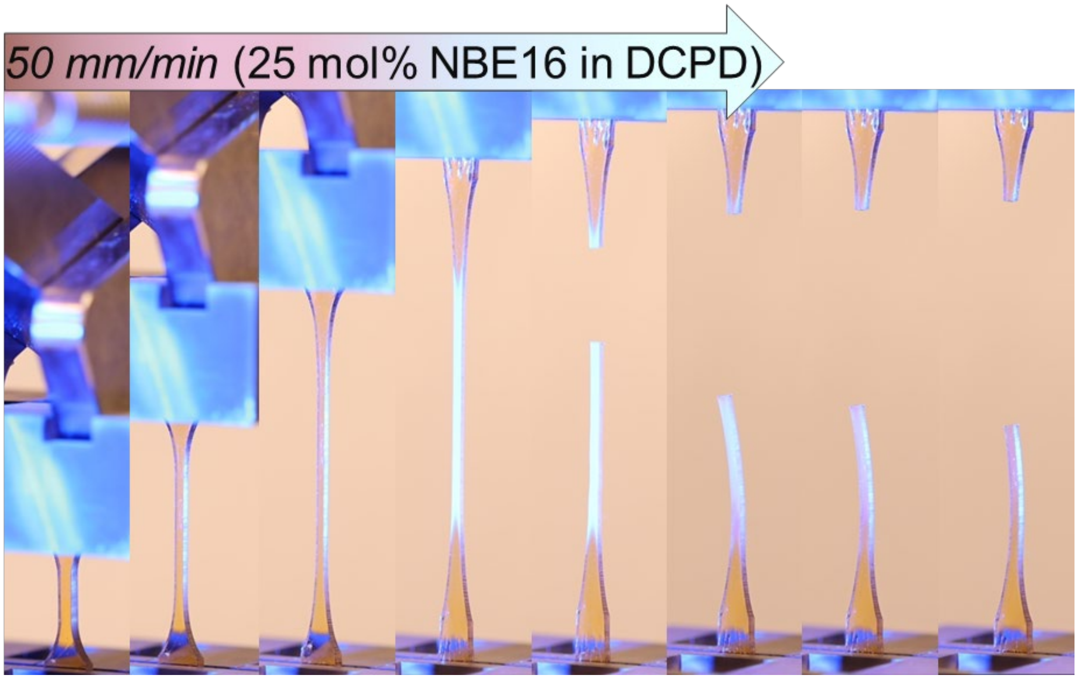

Figure S171: Representative timelapse of 1000:1:1 25 mol% NBE16 in DCPD-H_2:G2:TBP at 50 mm/min followed by rapid reversion of strain-whitening behavior after failure (first four frames are 30 s increments then 10-15 s).

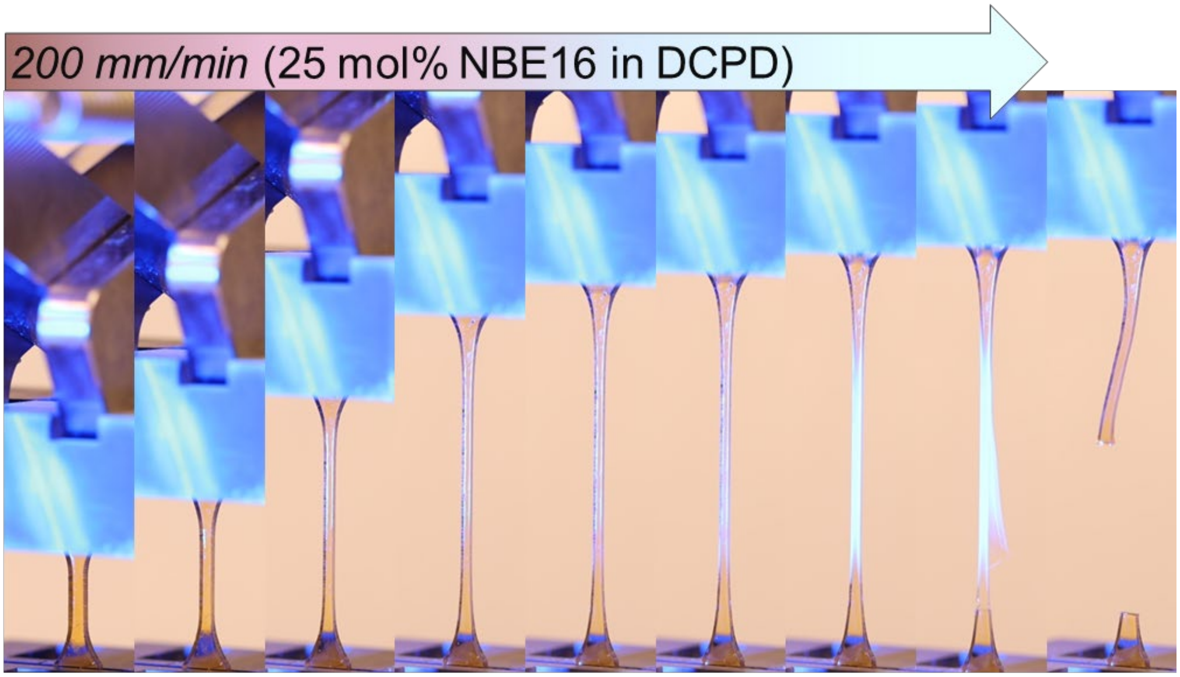

Figure S172: Representative timelapse of 1000:1:1 25 mol% NBE16 in DCPD-H_2:G2:TBP at 200 mm/min followed by rapid reversion of strain-whitening behavior after failure (first four frames are 5 s increments then 1 s increments).

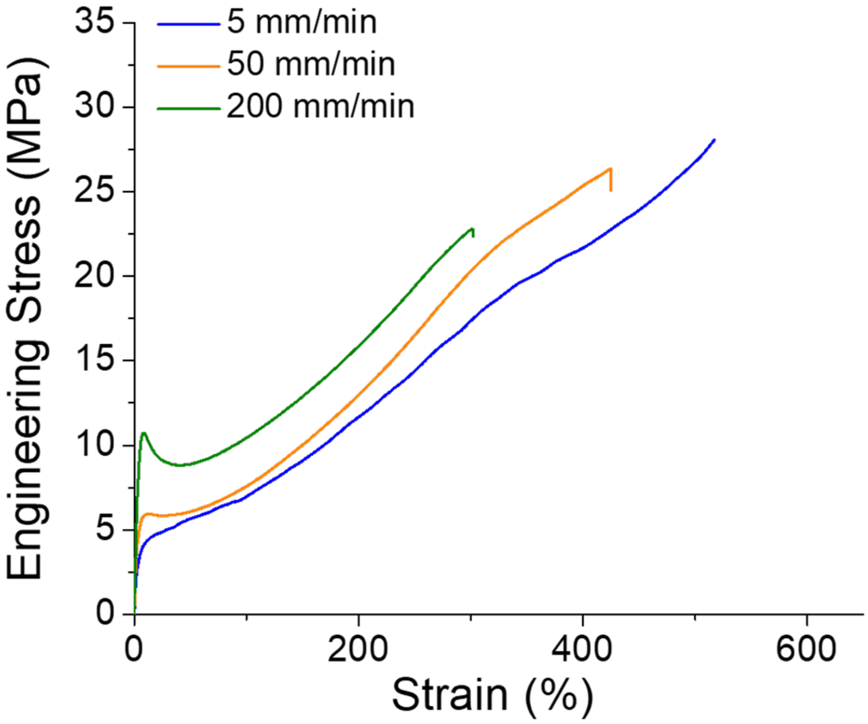

Figure S173: Representative stress-strain curves of 25 mol% NBE16 at 5, 50, and 200 mm/min strain rates.

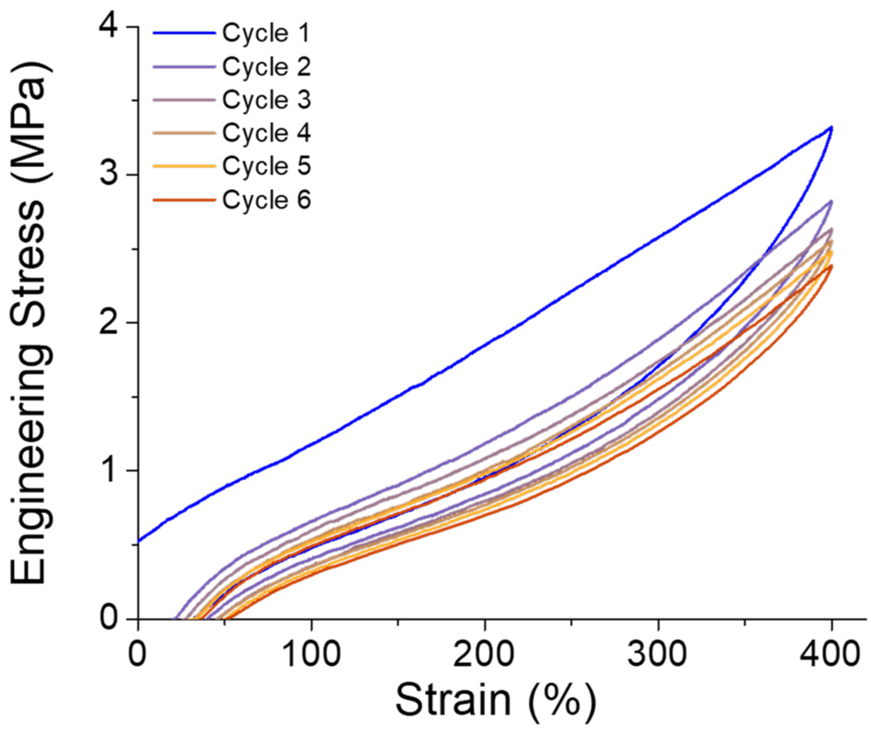

Figure S174: Representative hysteresis stress-strain curves 50 mol% NBE8.

Tensile Testing of Samples Containing DCPD-H_2:

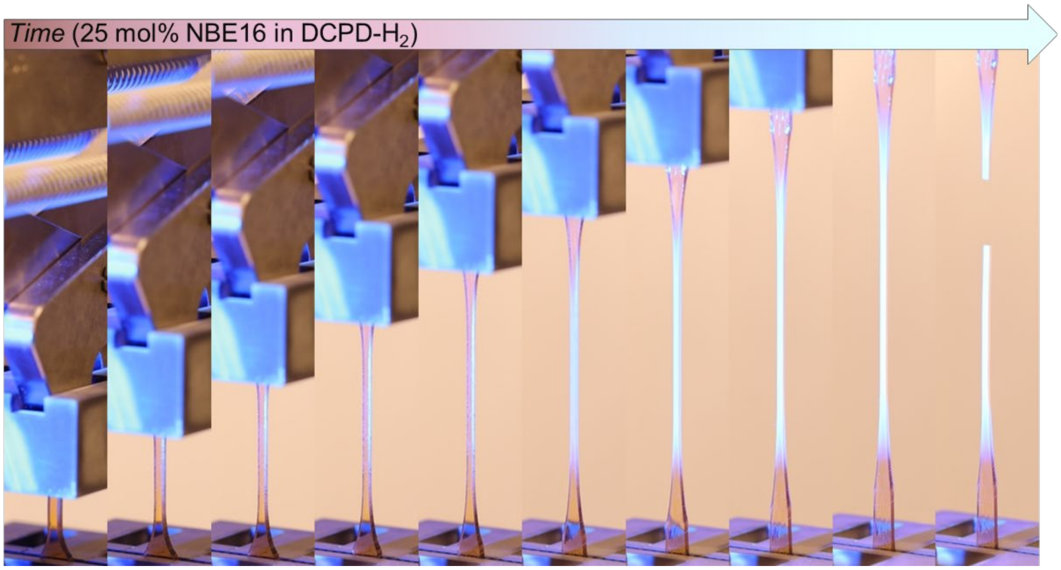

Figure S175: Representative timelapse of 1000:1:1 25 mol% NBE16 in DCPD-H_2:G2:TBP.

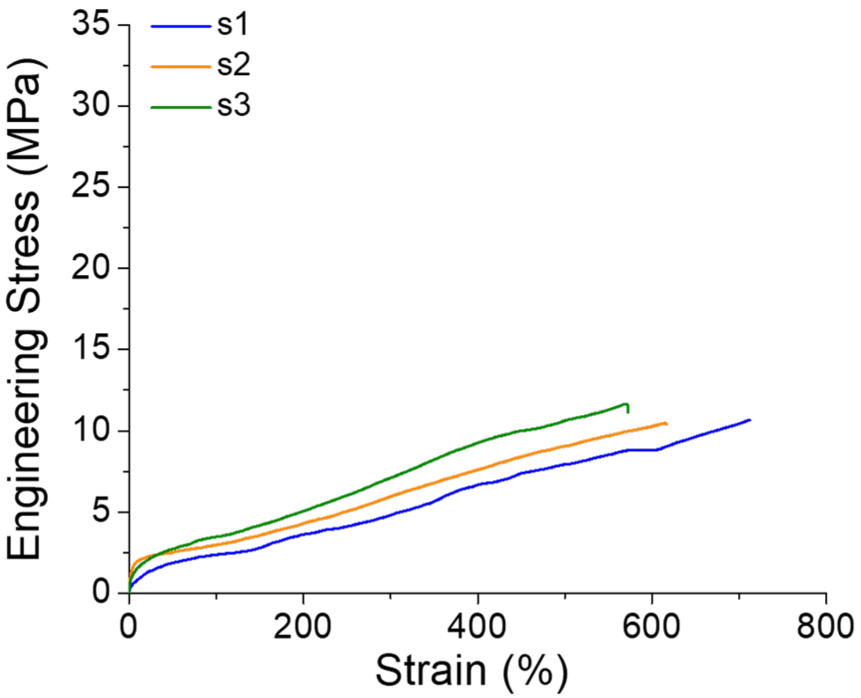

Figure S176: Stress-strain curves of 1000:1:1 25 mol% NBE16 in DCPD-H_2:G2:TBP (n = 3).

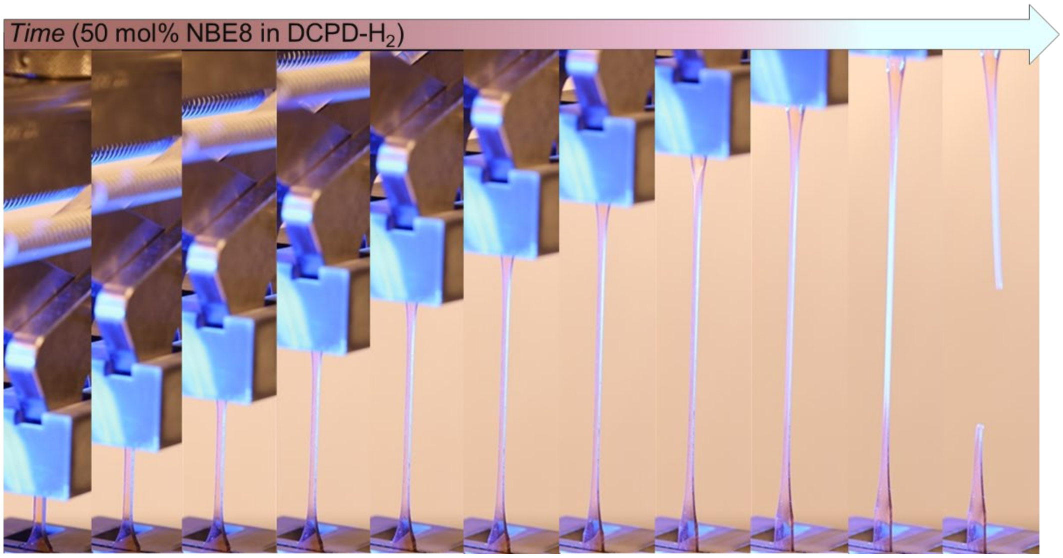

Figure S177: Representative timelapse of 1000:1:1 50 mol% NBE8 in DCPD-H_2:G2:TBP.

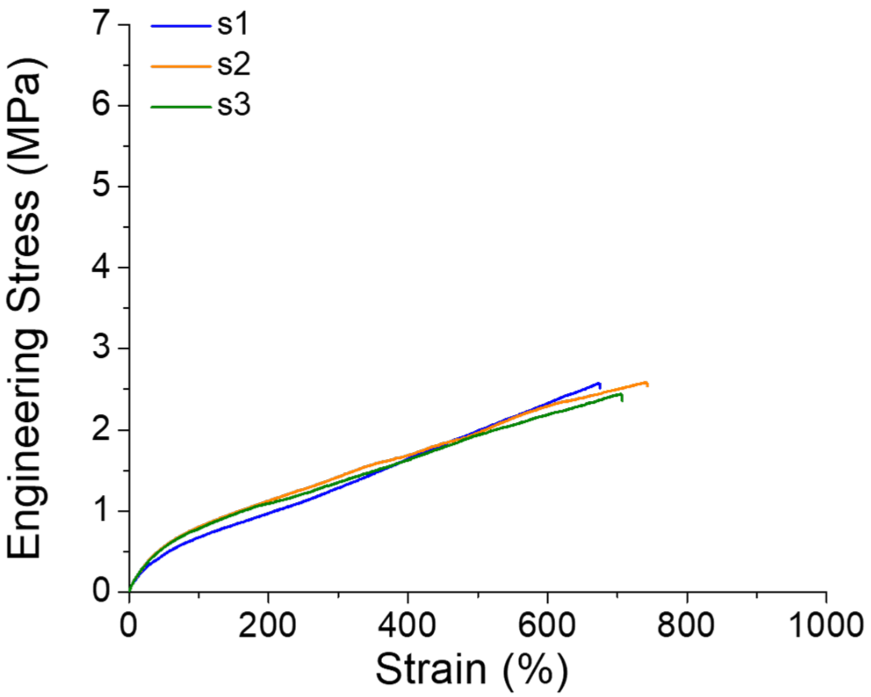

Figure S178: Stress-strain curves of 1000:1:1 50 mol% NBE8 in DCPD-H_2:G2:TBP (n = 3).

Table S14: Tensile data for NBE copolymer networks (n = 5) and select NBE linear copolymers (n = 3) post-FROMP at 1000:1:1 monomer:initiator:inhibitor.

Sample	Elongation at Failure (%)	error	Ultimate Tensile Stress (MPa)	error	Yield Strength (MPa)	error	Young's Modulus (MPa)	error	Stress at 100% Elongation (MPa)	error
50 mol% NBE8 (network)										
1000:1:1	842	28	5.32	0.37	N/D	N/D	6.48	3.25	0.87	0.04
25 mol% NBE8 (network)										
1000:1:1	178	16	46.0	2.5	36.3	1.3	1194	31	33.9	0.96
25 mol% NBE12 (network)										
1000:1:1	244	38	42.2	2.6	25.2	2.2	1003	73	24.0	1.11
25 mol% NBE16 (network)										
1000:1:1	519	40	25.9	1.4	N/D[a]	N/D[a]	284	38	6.82	0.47
50 mol% NBE8 (linear)										
1000:1:1	708	34	2.53	0.08	N/D[a]	N/D[a]	6.08	0.45	0.75	0.06
25 mol% NBE16 (linear)										
1000:1:1	634	72	10.9	0.6	N/D[a]	N/D[a]	111	45	2.94	0.45

[a]N/D indicates no obvious yield point (elastic to plastic deformation transition) was observed.

References:

(1) Alzate-Sanchez, D. M.; Yu, C. H.; Lessard, J. J.; Paul, J. E.; Sottos, N. R.; Moore, J. S. Rapid Controlled Synthesis of Large Polymers by Frontal Ring-Opening Metathesis Polymerization. *Macromolecules* **2023**, *56* (4), 1527-1533. DOI: 10.1021/acs.macromol.2c01892.
(2) Xu, Z.; Wang, K.; Suslick, B. A.; Moore, J. S. Circular Workflow for Thermosets: Activatable Repeat Unit Design for Regenerative Frontal Polymerization. *Journal of the American Chemical Society* **2025**, *147* (10), 8732-8740. DOI: 10.1021/jacs.4c18018.
(3) Stewart, K. A.; Ivannikava, D. A.; Massouh, C. M.; Lessard, J. J. Molecular Weight Control in Frontal Ring-Opening Metathesis Polymerization. *Angewandte Chemie International Edition n/a* (n/a), e202510071. DOI: https://doi.org/10.1002/anie.202510071.
(4) Mondal, S.; Lessard, J. J.; Meena, C. L.; Sanjayan, G. J.; Sumerlin, B. S. Janus Cross-links in Supramolecular Networks. *Journal of the American Chemical Society* **2022**, *144* (2), 845-853. DOI: 10.1021/jacs.1c10606.
(5) Nishimura, Y.; Chung, J.; Muradyan, H.; Guan, Z. Silyl Ether as a Robust and Thermally Stable Dynamic Covalent Motif for Malleable Polymer Design. *J. Am. Chem. Soc.* **2017**, *139* (42), 14881-14884. DOI: 10.1021/jacs.7b08826.
(6) Stewart, K. A.; Ivannikava, D. A.; Massouh, C. M.; Lessard, J. J. Molecular Weight Control in Frontal Ring-Opening Metathesis Polymerization. *Angewandte Chemie International Edition* **2025**, *64* (44), e202510071. DOI: https://doi.org/10.1002/anie.202510071.
(7) Lessard, J. J.; Mejia, E. B.; Kim, A. J.; Zhang, Z.; Berkey, M. G.; Medina-Barreto, Z. S.; Ewoldt, R. H.; Sottos, N. R.; Moore, J. S. Unraveling Reactivity Differences: Room-Temperature Ring-Opening Metathesis Polymerization (ROMP) versus Frontal ROMP. *Journal of the American Chemical Society* **2024**, *146* (11), 7216-7221. DOI: 10.1021/jacs.4c01578.
(8) Kumar, A.; Dean, L. M.; Yourdkhani, M.; Guo, A.; BenVau, C.; Sottos, N. R.; Geubelle, P. H. Surface pattern formation induced by oscillatory loading of frontally polymerized gels. *Journal of the Mechanics and Physics of Solids* **2022**, *168*, 105055. DOI: https://doi.org/10.1016/j.jmps.2022.105055.
(9) Dean, L. M.; Wu, Q.; Alshangiti, O.; Moore, J. S.; Sottos, N. R. Rapid Synthesis of Elastomers and Thermosets with Tunable Thermomechanical Properties. *ACS Macro Letters* **2020**, *9* (6), 819-824. DOI: 10.1021/acsmacrolett.0c00233.
